# Hybridization in the Cetacea: widespread occurrence and associated morphological, behavioral, and ecological factors

**DOI:** 10.1002/ece3.1913

**Published:** 2016-01-28

**Authors:** Carla A. Crossman, Eric B. Taylor, Lance G. Barrett‐Lennard

**Affiliations:** ^1^ Marine Mammal Research ProgramCoastal Ocean Research InstituteVancouver Aquarium Marine Science CentreVancouverBritish ColumbiaCanada; ^2^ Department of ZoologyUniversity of British ColumbiaVancouverBritish ColumbiaCanada; ^3^ Biodiversity Research Centre, and Beaty Biodiversity MuseumUniversity of British ColumbiaVancouverBritish ColumbiaCanada

**Keywords:** Cetaceans, hybridization, niche, principal component analysis, species traits

## Abstract

Hybridization has been documented in a many different pairs of cetacean species both in captivity and in the wild. The widespread occurrence of hybridization indicates that postmating barriers to interbreeding are incomplete within the order Cetacea, and therefore raises questions about how species integrity is maintained in the face of interspecific (and often intergeneric) gene flow. We examined hybridization across the order Cetacea (oceanic species included: *N* = 78; species with 44 chromosomes included: *N* = 52) to test for associations between the occurrence of hybridization and similarity across 13 ecological, morphological and behavioral traits in hybridizing vs. non‐hybridizing species pairs. We found that species pairs that share a greater number of traits had a higher propensity to hybridize than pairs of species that did not. This trend was driven by behavioral and morphological traits such as vocalization frequency and body size. Together our findings suggest the importance of divergent selection on morphological and behavioral traits within sympatric species in constraining opportunities for hybridization and preventing the collapse of parental species.

## Introduction

Aggregating in social groups can present both benefits and consequences for animals. Grouping behavior can facilitate prey detection and capture, and defence against predators (Alexander 1974); however, individuals living in groups may have higher incidences of parasitism, disease or inbreeding than individuals living alone (Côté and Poulin 1995; Loehle 1995; Pusey and Wolf 1996). Despite this, group living is exhibited by a wide array of animal taxa including insects, fishes, birds, and mammals (Alexander 1974). The benefits and consequences of these aggregations are not limited to groups of a single species; mixed species assemblages form for many of the same reasons as single species groups, but they introduce another potential consequence – interspecific hybridization.

Hybridization holds risks for the fitness of individual hybrid offspring (and hence the inclusive fitness of their parents). In some cases, hybridization may even limit the distribution or persistence of the parental species. Interspecific matings may be consensual or coercive “practice” matings that increase the chance of success in intraspecific matings and/or they may result from behavioral dominance of one species over another or from various types of social interactions (Vasey 2004). Additionally, they may be more likely to occur when potential mates of the same species are absent or at low abundance, as in the case of rare or depleted species. Interspecific mating has many obstacles to overcome in order to result in a viable hybrid (Orr 1995) and many hybrids that do survive are sterile, with higher instances of sterility in the heterogametic sex (Haldane 1922; Noor 1999). If hybrids suffer no negative fitness consequences or are, in fact, more fit than parental species, backcrosses may result and outcompete individuals of a parental species (Rhymer and Simberloff 1996; Huxel 1999; Mallet 2007).

Hybridization is common in some classes of vertebrates (e.g. birds, Grant and Grant 1992; fishes, Scribner et al. 1977), but is relatively rare in terrestrial mammals (Gray 1954). In contrast, a review of the literature (Tables 1 and 2) shows that almost 20% of species within the order Cetacea are known to hybridize. Cetaceans are a relatively recent radiation; most of the species diversity has arisen in the past 10 million years (McGowen et al. 2009; Slater et al. 1991). The recent radiation, combined with an apparently slow rate of molecular evolution (Hoelzel et al. 1991; Schlötterer et al. 2001), likely accounts for common chromosome number (2n = 44) and karyotic arrangement present in most cetaceans (Árnason and Benirschke 1973; Árnason et al. 1977; Pause et al. 2006). The proportion of oceanic cetaceans with 44 chromosomes in which hybridization is known to occur is 50% (Tables 1 and 2).

**Table 1:** Documented cases of cetacean hybridization in captivity

Paternal species	Maternal species	Source
*Sotalia guianensis*	*Tursiops truncatus*	Caballero and Baker (2010)
*Tursiops truncatus*	*Steno bredanensis*	Dohl et al. (1974); Shallenberger and King (1986)
*Grampus griseus*	*Tursiops truncatus*	Shimura et al. (2005); Miyazaki et al. (1992)
*Lagenorhynchus obliquidens*	*Tursiops truncatus*	Miyazaki et al. (1992)
*Tursiops truncates*	*Globicephala macrorhynchus*	Antrim and Cornell (1981)
*Delphinus capensis*	*Tursiops truncatus*	Zornetzer and Duffield (2003)
*Pseudorca crassidens*	*Tursiops truncatus*	Nishiwaki and Tobayama (1982)

**Table 2:** Documented cases of cetacean hybridization in the wild

Species 1	Species 2	Source
*Balaenoptera physalus*	*Balaenoptera musculus*	Spilliaert et al. (2004); Bérubé and Aguilar (1998)
*Delphinus capensis* (Probable)	*Lagenorhynchus obscurus*	Reyes (1996)
*Balaenoptera acutorostrata*	*Balaenoptera bonaerensis* (probable)	Glover et al. (2010)
*Tursiops truncates*	*Stenella frontalis*	Herzing et al. (2003)
*Grampus griseus*	*Tursiops truncatus*	Shimura et al. (2005); Miyazaki et al. (1992); Fraser (1940)
*Tursiops aduncas*	*Tursiops truncatus* (unknown)	Martien et al. (2012)
*Stenella attenuata*	*Stenella longirostiris*	Silva‐Jr et al. (2010)
*Stenella clymene*	*Stenella longirostiris*	Silva‐Jr et al. (2010)
*Lissodelphis peronei*	*Lagenorhynchus obscurus*	Yazdi (2002)
*Phocoena phocoena*	*Phocoenoides dalli*	Baird et al. (1998); Willis et al. (2003); Crossman et al. (2014)
*Pseudorca crassidens*	*Tursiops truncatus*	Nishiwaki and Tobayama (1982)
*Monodon monoceros*	*Delphinaptera leucas*	Heide‐Jørgensen and Reeves (1993)
*Tursiops truncatus*	*Sousa chienensis* (Possible)	Karczmarski et al. (1997)

**Figure 1:**
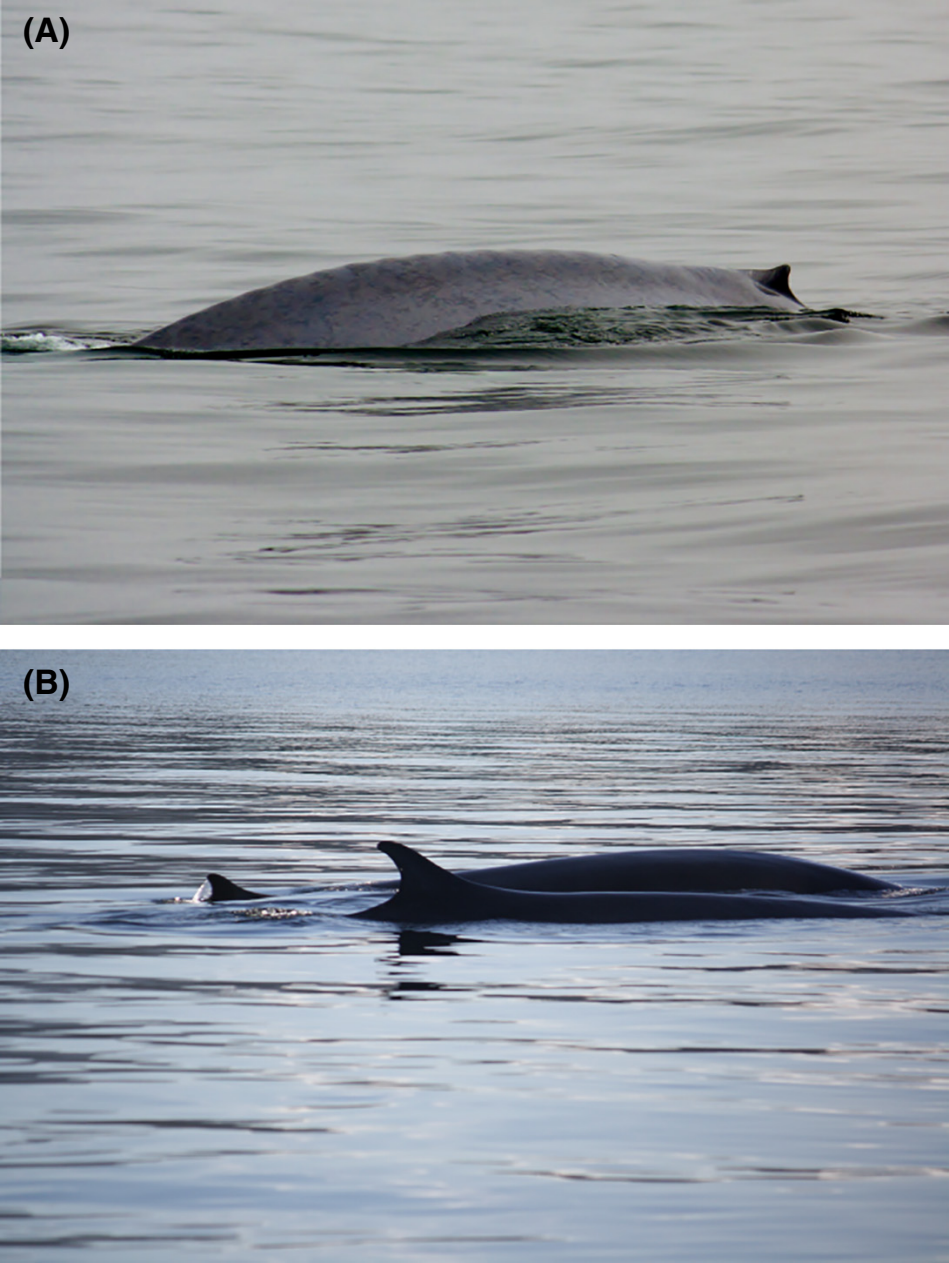
(A) Blue whales (*Balaenoptera musculus*) and (B) Fin whales (*Balaenoptera physalus*) are known to produce fertile hybrid offspring.

Hybrid cetaceans have been documented both in captivity and in the wild (see reviews by Sylvestre and Tasaka 2000 and Schaurich et al. 1991). While interspecific hybridization in captivity does not mean that it necessarily occurs in the wild, it does demonstrate the potential for different species to hybridize in nature. Identification of hybrid cetaceans dates back to the whaling industry in the 1800s; whaling records report captured specimens whose size and coloration were intermediate between those for blue whale (*Balaenoptera musculus*; Fig. 1A) and fin whale (*Balaenoptera physalus*; Fig. 1B) (Spilliaert et al. 2004). Confirmation of potential hybrids is best done using genetic techniques; however, samples are often difficult and expensive to collect from cetaceans and hybrid identifications have often been based on morphological evidence (e.g. Spilliaert et al. 2004; Herzing et al. 2003; Silva‐Jr et al. 2010). The most robust morphological determinations of hybridization have been done on dead specimens, in which more intermediate traits can be measured than in live specimens (e.g. number of vertebrae or teeth). In general, it is likely that many living hybrids seen in the wild are not recognized as such.

At least seven instances of hybridization in captivity have been recorded between pairs of cetacean species (Table 1). At least two of these hybrids were fertile and produced backcrossed offspring (Zornetzer and Duffield 2003; Maines and Kestin 2009), but the fertility of the others is unknown. While hybridization in captivity occurs under artificial conditions, many pairs of species also have natural range overlap, and are known to form mixed species assemblages see Table S1). This suggests that interspecific hybrids among these pairs of species are also possible in the wild.

Unfortunately, the survivorship and/or reproductive success of most wild hybrids is not known, and in many cases the sex of the hybrid is unknown. In both wild and captive species pairs, however, there is evidence of reproductively viable hybrids and successful backcrossing (Spilliaert et al. 2004; Baird et al. 1998; Odell and McClune 1999; Crossman et al. 2014). While fertility of some female hybrids has been confirmed, determining male fertility is challenging and therefore it is difficult to estimate the impact of hybridization at the population level.

While the long‐term impacts of hybridization on cetacean populations are not well understood, the absence of pre‐ and postmating, including postzygotic, barriers to hybridization in the order Cetacea raises several questions. What promotes or enables hybridization in cetaceans? Are there certain behavioral, morphological, or ecological traits that predispose certain species pairs to hybridize? In this study, we tested for associations between the extent to which species share certain traits and the incidence of hybridization across all species pairs of marine cetaceans.

## Materials and Methods

### Data collection

We conducted a literature search to collect data on the ecological, morphological, and behavioral traits of 78 extant species of marine cetaceans (Committee on Taxonomy 2009). Freshwater cetacean species, species with large information gaps and those with recent taxonomic revisions (where information on species traits for the new species could be obscured in information gathered for the original species) were excluded from the study (*N* excluded = 8). The following traits were examined: male body length, female body length, visible sexual dimorphism (size, colour etc.), group size (solitary, medium or social), species' range size (as a proxy measurement for individual range size; small, medium or large), water depth, water temperature, prey species, predator species, parasite species, known associated species, natural range overlap between each species pair and vocalization frequency (low, medium, or high). These were chosen to depict both gross morphological and behavioral characteristics of a species, as well as ecological traits associated with their specific niche (Aldridge and Rautenbach 1987; Gowans and Whitehead 1995; Vanhooydonck et al. 1998; Bearzi 2005). When possible, information was collected from sources incorporating various parts of the species' range (Table S1).

### Similarity index

For each trait, we calculated an index of similarity for each species' pair using similar methods to those presented by Geange et al. (2011). Two species that shared 100% of their traits received a similarity index of ‘1’ and pairs of species with no overlap in any traits had a similarity of ‘0’. Traits that were not described in the scientific literature for a given species were listed as ‘Not Available’ (NA) and were removed from the analysis for comparisons within all pairs for that species. The similarity of traits was calculated in five different ways depending on the type of trait data being assessed.

#### Presence/absence – (species pair range overlap, sexual dimorphism)

A matrix for natural range overlap of species pairs was built by assigning ‘1’ to pairs of species' that overlap, to any extent, in their ranges, and a ‘0’ where they do not (i.e., they are completely allopatric). Sexual dimorphism was examined in a similar fashion; if the two species are both sexually dimorphic or if neither is sexually dimorphic, we assigned them a similarity index of ‘1’. If one species shows dimorphism and the other does not, the pair was assigned a value of ‘0’.

#### Continuous traits – (male body length, female body length, water temperature)

These traits were analyzed as a continuous range of body size of each sex at physical maturity and of preferred water temperature. If the ranges of trait values for the two species did not overlap, we assigned the pair a similarity of ‘0’. If the trait value of the two species overlapped, we calculated the amount they overlapped and divided this value by the size of the smaller range in trait values of the two species (Eq. (1)). This resulted in a percentage of overlap of the trait relative to the more specialized species with the smaller range in trait values.

(1) $$\frac{\text{(Smaller of}\;{\text{Max}}_{1}\text{or}{\text{Max}}_{2})-\text{(Larger of}\;{\text{Min}}_{1}\text{or}{\text{Min}}_{2})}{\text{Smaller of}\;{\text{(Max}}_{1}-{\text{Min}}_{1}\text{or}{\text{Max}}_{2}-{\text{Min}}_{2})}$$

#### Continuous traits as categorical data – (group size, species' range size, water depth, vocalization frequency)

We took the average value of each trait for each species and grouped them into categories: group size (solitary: 1–5 individuals, medium: 5–50 individuals; social: 50+ individuals), species' range size (small: <10^6^ km^2^; medium: 10^6^–10^7^ km^2^; large: >10^7^ km^2^), water depth (shallow: 0–200 m; medium: 200–1000 m; deep: >1000 m), and vocalization frequency (low: 0–5 kHz; medium: 5–10 kHz; high: >10 kHz). If two species occupy the same category (i.e. both solitary), we assigned the pair an index of ‘1’. If two species occupy categories at either end of the spectrum (i.e. one species with small range size and one with large range), the pair was assigned ‘0’. If one of the species occupies an intermediate value and the other does not (i.e. one species from medium water depth and one from deep water), we assigned the pair a similarity index of ‘0.5’.

#### Categorical traits – (prey species, predator species, parasite species, known associate species)

We examined prey, predators and parasites of cetaceans at the family level to help account for global variation in species' distribution. For these traits, we calculated the number of shared families or species of prey, predators, and/or parasites, and divided that by the total number of families or species encountered by both cetacean species (Eq. (2)). We used this value as the similarity between species pairs for these categorical traits.

(2) $$\frac{\text{Number of shared prey, predator, parasite families or known associate species}}{\text{(No. of families or species uniquely interacting with species 1 + no. of families or species uniquely interacting with species 2)}}$$

#### Total similarity index

The total similarity index was calculated by taking both an un‐weighted and a weighted average of the similarities of each trait. The un‐weighted index was simply the mean of all similarity indices for each trait for each species pair (Eq. (3)).

(3) $$\frac{\sum \text{Individual trait similarity indices}}{\text{Number of traits}}$$

A weighted average was calculated by conducting a survey of professional opinion regarding the relative importance each trait might have on the predisposition of species to hybridize (Supp. S2). These weightings were averaged across survey participants and each trait received a weight that represented a proportion of the predisposition to hybridize. We applied each the weightings to their respective traits and summed these to achieve a weighted index of similarity again from ‘0’ to ‘1’ (Eq. (4)).

(4) $$\sum_{i=1}^{\mathrm{Number}\;\;\mathrm{of}\;\;\mathrm{traits}}{\text{weight}}_{i}\;\;\text{trait similarity}\;{\text{index}}_{i}$$

### Hybridization and similarity index

In order to assess whether species that have been known to hybridize are more or less similar in the traits described above than species pairs that do not hybridize, we conducted a Mantel test in R v.2.12.2 (R Project for Statistical Computing) using the Kendall method from the package VEGAN (Oksanen et al. 2011). We compared the matrix of trait similarity and a matrix of all possible species pairs where ‘0’ represents a nonhybridizing pair, and ‘1’ indicates a known hybridization event. We omitted the diagonal from the analyses to avoid a bias as it represents the mating between the same species and will always has a similarity of ‘1’. We also conducted a nonparametric Wilcoxon sum rank test in R to test whether hybridizing pairs of species have higher indices of similarity than nonhybridizing pairs. Similarity indices for the 18 hybridizing pairs of species were tested against 18 nonhybridizing pairs sampled at random using 10,000 iterations to minimize the effect of unbalanced sample sizes.

In order to determine which traits might be driving trends in either similarity or dissimilarity, we conducted a principal components analysis (PCA) in R using the covariance matrix. We included all 13 species traits as factors and used the *prcomp()* function in R for increased numerical accuracy and because the inputted trait matrix was not composed of raw values, but instead of a calculated index. We present all principal components that account for at least 10% of the variance.

Assuming that a different number of chromosomes or chromosomal arrangement will prevent or strongly deter hybridization (Dobzhansky 1935), we repeated the analyses using only cetaceans with the same chromosomal number. Therefore, those 26 species (beaked whales, right whales and sperm whales) with 42 chromosomes (Árnason and Benirschke 1973; Árnason et al. 1977; Pause et al. 2006) were removed from the analyses. The Mantel test, Wilcoxon sum rank test and the PCA with all comparisons were performed twice for both all species comparisons and for species with 44 chromosomes: once with the raw similarity index, and again with the similarity index weighed according to expert opinion.

Our data for the PCA were pairwise comparisons where individual species were represented several times and therefore violate an assumption of the PCA that all observations are independent. We included these results to allow for comparison of the relative importance of each trait, but also conducted our analyses after removing pairwise comparisons. This analysis consisted of subsampling random pairs of species, so that each species was represented in a single comparison, and we used the similarity index of these randomly chosen pairs to conduct a second PCA. We replicated this PCA 10,000 times using different combinations of independent species pairs. We averaged the absolute value of the eigenvectors for each trait in each of the first four principal components to account for variations across the 10,000 iterations and to obtain a single eigenvector for each trait.

## Results

### Un‐weighted analysis

**Figure 2:**
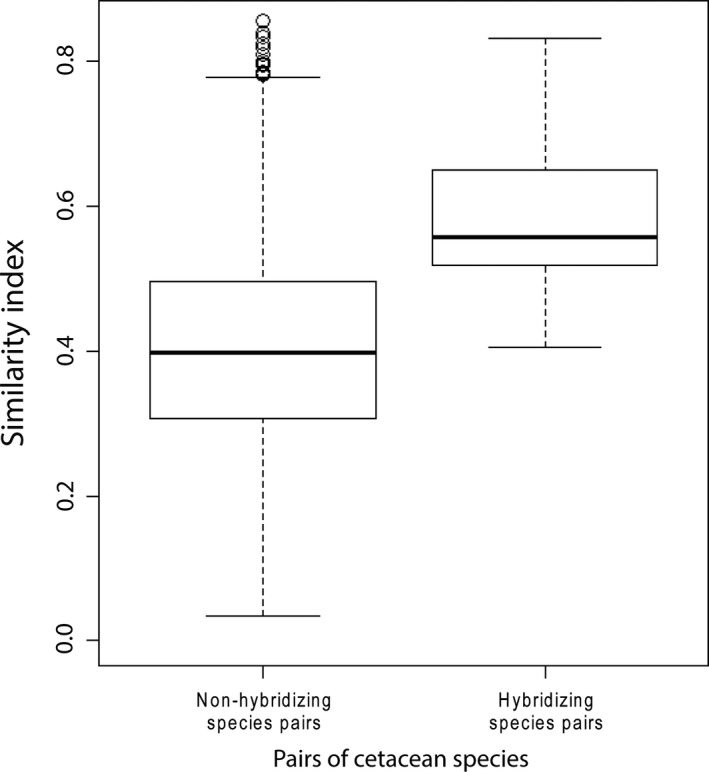
Similarity index of nonhybridizing species pairs (*n* = 6048) and hybridizing species pairs (*n* = 36) for all cetacean species comparisons.

Pairs of species that are known to hybridize were more similar in ecological and morphological traits than species pairs that do not (Mantel test: *r *=* *0.076, *P *=* *0.001, Fig. 2). The subsampled Wilcoxon sum rank test revealed a similar pattern across 10,000 iterations (averaged *P*‐value = 0.002).

A principal components analysis accounted for 68.8% of the variation in the traits examined across the first four PC axes (Table 3). The first principal component, which accounted for almost 25% of the variation in traits, was driven strongly by similarities in the extent of sexual dimorphism between the species, with species range size, body length of both sexes and vocalization frequencies also playing strong roles having the greatest (+ or −) eigenvectors (Table 3). In the next three principal components (PC2 – PC4), similarities in water temperature and natural range overlap were the more important features with body length again contributing to the similarity. Average group size was also a strong contributor to PC2.

**Table 3:** Eigenvectors of the first four principal components of variation in similarity of traits for oceanic cetacean species with 42 and 44 chromosomes (*N* = 78). Variables that are more important for each principal component have larger values (+ or −)

Trait (All)	PC1	PC2	PC3	PC4
Male body length	−0.283	−0.316	0.282	−0.397
Female body length	−0.282	−0.302	0.354	−0.364
Sexual dimorphism	−0.628	0.432	−0.200	−0.044
Range size	−0.432	0.298	−0.122	−0.022
Water depth	−0.187	−0.007	−0.145	0.150
Water temperature	−0.162	−0.448	−0.454	0.443
Prey species	−0.136	−0.085	0.019	−0.033
Predator species	−0.122	−0.122	−0.159	−0.036
Parasite species	−0.155	−0.082	0.036	−0.023
Average group size	−0.190	−0.263	0.072	0.322
Known associate species	−0.126	−0.093	−0.010	−0.021
Natural range overlap	0.017	−0.386	−0.579	−0.460
Vocalization frequency	−0.241	−0.278	0.383	0.412
Proportion of variation accounted for	24.38%	19.78%	14.61%	10.07%

**Figure 3:**
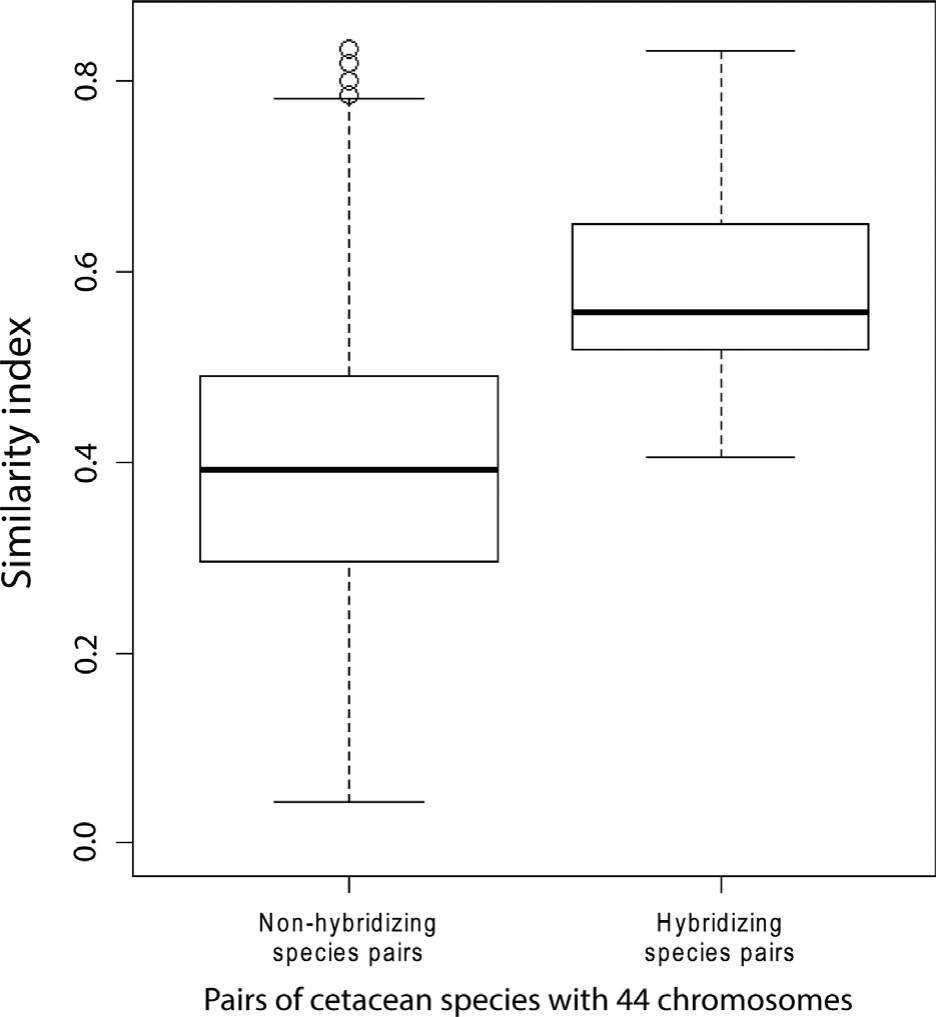
Similarity index of nonhybridizing species pairs (*n* = 2668) and hybridizing species pairs (*n* = 36) for cetacean species with 44 chromosomes.

The results were consistent for the subset of species with the same chromosomal number (2n = 44) (Mantel test: *r *=* *0.116, *P *=* *0.001, Fig. 3; Wilcoxon sum rank test 10,000 iterations mean *P*‐value = 0.005). The principal component analysis also suggested that the same traits seem to be contributing to the variation in similar proportions in each of the first few principal components (Table 4).

**Table 4:** Eigenvectors of the first four principal components of variation in similarity of traits for oceanic cetacean species with 44 chromosomes (*N* = 52). Variables that are more important for each principal component have larger values (+ or −)

Trait (2n = 44)	PC1	PC2	PC3	PC4
Male body length	−0.345	−0.290	0.197	0.455
Female body length	−0.353	−0.276	0.279	0.418
Sexual dimorphism	−0.533	0.595	−0.126	0.019
Range size	−0.353	0.399	−0.073	0.016
Water depth	−0.165	0.068	−0.114	−0.132
Water temperature	−0.208	−0.322	−0.531	−0.348
Prey species	−0.153	−0.050	−0.019	0.031
Predator species	−0.134	−0.060	−0.215	0.051
Parasite species	−0.175	−0.045	0.003	−0.016
Average group size	−0.244	−0.224	−0.021	−0.320
Known associate species	−0.141	−0.064	−0.053	0.032
Natural range overlap	−0.036	−0.251	−0.631	0.357
Vocalization frequency	−0.353	−0.305	0.345	−0.495
Proportion of variation accounted for	25.14%	19.40%	15.17%	10.58%

The 10,000 replicated PCAs with sub‐sampled pairs of species yielded similar results to those comparisons done across all species; the traits with the strongest influence in the first principal component were sexual dimorphism, range size, water temperate, natural range overlap, and vocalization frequency. The same traits showed large eigenvectors along PC2, and body size of both males and females had consistently strong eigenvector across the first four principal components (Table S3). In analyses of species with 44 chromosomes, sexual dimorphism, water depth, and range size were traits with the strongest influence on PC1, with overlap and vocalization frequency also contributing to the variance (Table S4). The relative influence of each of the traits was similar to their importance from the PCA with all pairs of species.

### Weighted analysis

The survey of expert opinion elicited 41 responses, and we calculated the average weighting for each trait (Table 5). The new weightings had little influence on the results and the ability to hybridize was still significantly correlated with similarity in morphological and ecological traits (Mantel test – all species: *r = *0.077, *P = *0.001, Figure S5; 44 chromosomes: *r = *0.116, *P = *0.001; Wilcoxon Sum Rank Test 10,000 iterations mean *P* value – all species: *P *=* *0.002, 44 chromosomes: *P *=* *0.002). The PCA accounted for 75.0% of the variation in the traits examined across the first four PC axes (Table S6), and for all species comparisons indicated slight differences in which traits may be driving this pattern (Table S6). The first PC accounted for 26% of the variation and was driven largely by natural range overlap, water temperature, sexual dimorphism, and range size. In the next three PCs (PC2‐PC4), many of the driving traits were the same as in the un‐weighted analysis, with more influence from natural range overlap. In PC2, the variable “known associated species” was also a large contributing factor. The analysis of the subset of species with 44 chromosomes showed very similar patterns regarding the influence of each trait (Table S7).

**Table 5:** Results of the survey (*N* = 41) to calculate weighted traits for assessing relative influence of each trait would have on the ability of pairs of cetacean species to hybridize

Trait	Range of weightings	Average
Male body length	0–0.250	0.085
Female body length	0–0.250	0.080
Sexual dimorphism	0–0.400	0.088
Range size	0–0.273	0.067
Water depth	0–0.200	0.086
Water temperature	0–0.136	0.086
Prey species	0–0.116	0.055
Predator species	0–0.105	0.032
Parasite species	0–0.136	0.035
Average group size	0–0.182	0.059
Known associate species	0–0.500	0.112
Natural range overlap	0–0.450	0.120
Vocalization frequency	0–0.210	0.095

## Discussion

Hybridization in the wild has three requirements: heterospecific mates must be genetically and physiological compatible; be behaviorally predisposed to mate; and, have overlapping distributions. This study sheds light on both reproductive compatibility and behavioral predisposition. Our literature review shows that compared to terrestrial mammals, intermating compatibility is extremely high among cetacean species, with many well‐defined intergeneric and intrageneric pairs of species able to produce viable hybrid offspring and, in at least some cases, viable backcrosses. The results of our analyses suggest that pairs of cetacean species that have been shown to hybridize are more similar in their morphological and behavioral traits, than their nonhybridizing counterparts. The absolute values of the correlation (*r*‐values) between similarity in pairs of species that do or do not hybridize are constrained to low values because there are so many more pairs of species that are not known to not hybridize (*n* = 6048) than pairs of species that do hybridize (*n* = 36). The positive relationship between morphological and behavioral similarity and propensity of cetacean species to hybridize appeared to be driven largely by similarities in the extent of sexual dimorphism, body length, geographic range size (as a proxy measurement for individual home range size), and vocalization frequency. Similarities in body size and state of sexual dimorphism among hybridizing pairs of species may reflect the importance of those characteristics for successful completion of the mating act. The effect of vocalization frequency that we found suggests a role for acoustic communication in courtship or some other behavior associated with mating. The observation that similarity in the size of the entire species' range (and by extension the travel patterns of individuals) is positively associated with the propensity to hybridize, may also influence the likelihood of groups of conspecific individuals travelling together and subsequently mating. In other words, the fact that sexual dimorphism, body length, geographic range size, and vocalization frequency are associated with hybridization implies that they play significant roles in mate choice in cetaceans in general. While the role of these factors in mate choice has not been studied in cetaceans, mate preference in other species (such as birds, terrestrial mammals and fishes) is often based in part on vocal behavior (e.g. zebra finches: Miller 1979; silvereye: Robertson 1996; greater spear‐nosed bats: Wenrick Boughman and Wilkinson 2002) and/or physical appearance (e.g. Darwin's finches: Ratcliffe and Grant 1983; damselflies: Gorb 1998; fish: Rowland 1999).

Similar studies looking at correlates with hybridization have been conducted in other species. Randler (2006) cited level of parental care and population sizes as key drivers of hybridization in birds. Alternatively, Jahner et al. (2011) found climactic variables (e.g. seasonal temperatures) can influence levels of hybridization in *Colias* butterflies. Several studies in fishes (Hubbs 1955; Scribner et al. 1977; Taylor 1995) have suggested that demographic and life history traits such as relative abundance, body size at maturity, timing of reproduction, and behavior can strongly influence the propensity to hybridize. Furthermore, habitat disturbance has long been a factor considered to promote interspecific hybridization in fishes (Hubbs 1955). Our study is, however, the first study to our knowledge that takes a meta‐analysis approach to examine correlates of hybridization in any marine mammal.

### Phylogenetic relatedness

Most pairs of hybridizing species of Cetacea that we examined were not closely related to one another. While all known pairs of hybridizing species belonged to the same family, only four of the 18 pairs of hybridizing species represented sister species, only two of the 18 pairs belonged to the same genus and of the 14 pairs within the Delphinidae family, only eight pairs belonged to the same subfamily. A similar study of birds showed no influence of phylogenetic relatedness on the tendency to hybridize (Randler 2006).

### Maintaining species barriers

The apparent propensity and ability of cetaceans to hybridize raises obvious questions about how species barriers are maintained in the order—especially when virtually all the hybridizing species discussed here have overlapping ranges in the wild. It is beyond the scope of this paper to explore this question in detail, but our findings lend support to the fact that (1) hybrids suffer a fitness disadvantage relative to their pure‐strain counterparts; (2) the relatively rare occurrence of hybrids is a result of premating isolating mechanisms; and (3) the reproductive isolation of many cetacean species is incomplete–small amounts of gene‐flow occur between them, and likely has done so for much of their history. Besides the obvious implications of this latter assertion for the accuracy of molecular analyses for estimating times of divergence, this continual low‐level gene flow may well prevent in some cases the development of complete postmating isolating mechanisms through genetic drift or other means.

### Maintenance of hybrids

In the case of cetaceans, the presence of widespread hybridization could reflect persistent hybrid zones or ‘tension zones’ between many different pairs of species. Tension zones occur in areas where hybrids still may exhibit relatively low fitness; however, these costs are balanced at some level by recurrent dispersal by parental species into areas where inter‐specific matings can then occur (Barton and Hewitt 1985). In tension zones, hybrid fitness is not linked to specific environmental conditions, but geographic features can influence dispersal and, therefore, the frequency of hybridization (Barton and Hewitt 1985). Given that the marine habitats of most cetaceans have few absolute geographic barriers, and that marine cetaceans have extensive dispersal potential, hybrid zones, *per se*, might be difficult to identify and have ever‐changing boundaries (e.g. Palumbi 1992, 1994; Norris 2000). While large scale geographic barriers may not exist, it is important to note that heterogeneity in the ocean in the form of ecotones and restricted dispersal (e.g. freshwater‐marine transitions) could play a role in the maintaining the separation of some species (Kark and van Rensburg 2007). Without known levels of hybridization over time, however, it is impossible to ascertain whether or not hybridization is decreasing or remaining stable either within specific pairings of species or overall within the Cetacea. Information obtained from implementing long‐term genetic monitoring programs could enable documentation of hybridization events and their trends through time.

### Potential benefits of interspecific mating

In cetaceans, mating behavior is not just limited to single adult male‐female pairs during a single, fixed breeding season. Mating behavior is witnessed year‐round (e.g., Shane et al. 1985) and is often seen between individuals of different age classes (e.g., calves, Herzing 1997), same sex pairs (e.g., male‐male copulations, Mann 2006), and even interspecific pairs (e.g., Stenella and Tursiops; Herzing et al. 2003). So why would cetaceans devote time and energy to these activities that have no direct reproductive potential? There are, however, several potential benefits to these interspecific pairing that may help to explain their occurrence.

First, it has been hypothesized that cetaceans exhibit mating‐like behavior as a form of social play (Brown and Norris 1956; Herzing and Johnson 1997). Social play is expressed through sexual behavior in other mammalian taxa such as primates (Vasey 2004). This social play could be solely for “entertainment”, or it could be used to establish a dominance hierarchy between individuals (Vasey 2004). Established dominance roles can be important for daily interactions between individuals in a larger group. Alternatively, these ongoing mating attempts could serve a learning role that enhances eventual probability of successful matings, especially for males (Mann 2006). A male that is able to practice mating, even with another species, with no negative consequences (e.g., from outbreeding depression) may have a higher chance of reproductive success during the breeding season compared to males with no such previous experience, and may therefore realize an increase in the probability and number of offspring that season. While to our knowledge no direct evidence of this exists, males are frequently seen mating with animals of different age/sex classes (Herzing 1997; Mann 2006) even outside of the season when females are in estrous (Shane et al. 1985).

## Conclusions

The results of our study suggest that pairs of species known to hybridize are more similar in some of their ecological, morphological, and behavioral traits than those which have not. This pattern appears to be driven by traits that could contribute to species recognition via visual and acoustic means (e.g. sexual dimorphism, body length, vocalization frequency) and suggests that a poor ability to discriminate between species may lead to increased cases of hybridization. The detection of potential cetacean hybrid zones and their structure will require long term genetic monitoring programs to examine the fate of hybrids and of parental species. In addition, our analyses reported hybridization as a binary variable, i.e., it has or has not been reported at least once. More detailed genetic monitoring will be necessary to quantify the actual extent (e.g., proportion of hybrids across population samples – see Crossman et al. 2014) and rates of naturally occurring cetacean hybridization. Finally, it should be noted the number of species pairs known to hybridize is a minimum estimate of the true number.

## Conflict of Interest

None declared.

## Supporting information


**Table S1**. Values for key morphological, ecological and behavioural traits in 78 species obtained from literature and literature reviews.
**Table S2**. Survey Template for Professional Opinion of Strength of Driving Factors
**Table S3**. Eigenvectors of the first four principal components of variation in similarity of traits for all cetacean species comparisons by taking the absolute value of the eigenvectors averaged across 10,000 subsampled principal component analyses where each species was only represented once.
**Table S4**. Eigenvectors of the first four principal components of variation in similarity of traits for cetacean species with 44 chromosomes by taking the absolute value of the eigenvectors averaged across 10,000 subsampled principal component analyses where each species was only represented once.
**Figure S5**. Weighted similarity index of non‐hybridizing species pairs (n = 6048) and hybridizing species pairs (*n* = 36) for all species comparisons.
**Table S6**. Eigenvectors of the first four principal components of variation in the weighted similarity of traits for all cetacean species comparisons (*N* = 78).
**Table S7**. Eigenvectors of the first four principal components of variation in the weighted similarity of traits for cetacean species comparisons with 44 chromosomes (*N* = 52).
