# Supplementary material for: Hybridization in the Cetacea: widespread occurrence and associated morphological, behavioral, and ecological factors
Source: Ecol Evol. 2016 Jan 28;6(5):1293–303. doi: 10.1002/ece3.1913 (PMC4775523; doi:10.1002/ece3.1913)
Supplement: Supplementary file 1 — Table S1. Values for key morphological, ecological and behavioural traits in 78 species obtained from literature and literature reviews. Table S2. Survey Template for Professional Opinion of Strength of Driving Factors Table S3. Eigenvectors of the first four principal components of variation in similarity of traits for all cetacean species comparisons by taking the absolute value of the eigenvectors averaged across 10,000 subsampled principal component analyses where each species was only represented once. Table S4. Eigenvectors of the first four principal components of variation in similarity of traits for cetacean species with 44 chromosomes by taking the absolute value of the eigenvectors averaged across 10,000 subsampled principal component analyses where each species was only represented once. Figure S5. Weighted similarity index of non‐hybridizing species pairs (n = 6048) and hybridizing species pairs (n = 36) for all species comparisons. Table S6. Eigenvectors of the first four principal components of variation in the weighted similarity of traits for all cetacean species comparisons (N = 78). Table S7. Eigenvectors of the first four principal components of variation in the weighted similarity of traits for cetacean species comparisons with 44 chromosomes (N = 52). [file ECE3-6-1293-s001.docx]

## Appendix

Table S1 Values for key morphological, ecological and behavioural traits in 78 species obtained from literature and literature reviews.

| Species | Length  MaleMin | Length MalMax | Length FemMin | Length FemMax | Sexually Dimorphic | Species' Range Size | Water Depth | Average Group Size | Prey |
| --- | --- | --- | --- | --- | --- | --- | --- | --- | --- |
| \| **Andrew's Beaked Whale (*Mesoplodon bowdoini*)** \| \| --- \| | 3.9 | 4.4 | 3.94 | 4.87 | No | Medium | NA | NA | NA |
| \| **Arnoux's Beaked Whale**  **(*Berardius arnuxii*)** \| \| --- \| | 8 | 9.34 | 8.84 | 9.33 | No | Medium | Medium | Medium | NA |
| Baird's Beaked Whale (*Berardius bairdii*) | 9.5 | 11.9 | 10 | 12.8 | Yes | Medium | Deep | Medium | Moridae, Gonatidae, Macrouridae, Cradchiidae |
| \| **Blainville's Beaked Whale**  **(*Mesoplodon densirostris*)** \| \| --- \| | 3.49 | 4.15 | 3.89 | 3.98 | Yes | Large | Deep | Solitary | Cepolidae, Melamphaidae, Myctophidae |
| \| **Cuvier's Beaked Whale**  **(*Ziphius cavirostris*)** \| \| --- \| | 5.5 | 6.93 | 5.27 | 7.54 | Yes | Large | Deep | Solitary | Histioteuthidae, Gonatidae, Cranchiidae, Ommastrephidae, Vampyroteuthidae, Bolitaenidae, Stauroteuthidae |
| \| **Gervais' Beaked Whale  (*Mesoplodon europaeus*)** \| \| --- \| | 3.54 | 4.56 | NA | NA | Yes | Medium | Deep | Solitary | Stomiidae, Octopoteuthidae, Lophogastridae |
| \| **Ginkgo-Toothed Beaked Whale (*Mesoplodon ginkgodens*)** \| \| --- \| | NA | NA | NA | NA | Yes | Large | NA | NA | NA |
| \| **Gray's Beaked Whale**  **(*Mesoplodon grayi*)** \| \| --- \| | NA | NA | NA | NA | Yes | Medium | Deep | Solitary | Merluccidae, Phosichthyidae, Myctophidae |
| \| **Hector's Beaked Whale**  **(*Mesoplodon hectori*)** \| \| --- \| | 3.65 | 4.34 | NA | NA | Yes | Medium | NA | Solitary | Octopoteuthidae |
| \| **Hubb's Beaked Whale**  **(*Mesoplodon carlhubbsi*)** \| \| --- \| | 4.96 | 5.3 | 4.9 | 5.32 | Yes | Medium | NA | NA | Gonatidae, Mastigoteuthidae, Melamphaidae, Onychoteuthidae, Histioteuthidae, Myctophidae, Stomiidae, Octopoteuthidae |
| Pygmy Beaked whale (*Mesoplodon peruvianus*) | 3.26 | 3.72 | NA | NA | Yes | Medium | NA | Solitary | Myctophidae, Nemipteridae |
| \| **Longman's Beaked Whale**  **(*Mesoplodon pacificus*)** \| \| --- \| | NA | NA | NA | NA | Yes | Large | Deep | Solitary | Cranchiidae, Onychoteuthidae, Chiroteuthidae, Histioteuthidae |
| \| **Northern Bottlenose Whale**  **(*Hyperoodon ampullatus*)** \| \| --- \| | 7.3 | 9.8 | 6 | 8.7 | Yes | Medium | Deep | Solitary | Gonatidae, Myopsidae, Oegposidae, Clupeidae, Gadidae |
| \| **Southern Bottlenose Whale**  **(*Hyperoodon planifrons*)** \| \| --- \| | NA | 6.94 | 5.7 | 7.45 | Yes | Medium | Deep | Solitary | Cranchiidae, Onychoteuthidae, Enoploteuthidae, Neoteuidae, Psychroteuthidae, Gonatidae |
| \| **Sowerby's Beaked Whale**  **(*Mesoplodon bidens*)** \| \| --- \| | 4.09 | 5.5 | 4.1 | 5.1 | No | Medium | Shallow | Solitary | Gadidae, Merluccidae |
| \| **Straptoothed Whale**  **(*Mesoplodon layardii*)** \| \| --- \| | 4.87 | 5.5 | 5 | 6.25 | Yes | Medium | NA | Solitary | Vampyroteuthidae, Chiroteuthidae, Cranchiidae, Cycloteuthidae, Gonatidae, Histioteuthidae, Mastigoteuthidae, Octopoteuthidae, Ommastrephidae, Onchoteuthidae |
| \| **Stejneger's Beaked Whale  (*Mesoplodon stejnegeri*)** \| \| --- \| | 3.89 | 5.3 | 4.34 | 5.24 | No | Medium | Deep | Solitary | Gonatidae, Cranchiidae |
| \| **Tasman Beaked Whale  (*Tasmacetus shepherdi*)** \| \| --- \| | 5.96 | 7.35 | NA | NA | No | Medium | Deep | Solitary | Merlucciidae, Serranidae, Bythitidae |
| \| **True's Beaked Whale  (Mesoplodon mirus)** \| \| --- \| | 4.6 | 5.3 | 4.87 | 5.18 | Yes | Medium | NA | NA | Cranchiidae, Loliginidae |
| \| **Atlantic Humpbacked Dolphin  (*Sousa teuszii*)** \| \| --- \| | 2 | 2.48 | NA | 2.35 | Yes | Small | Shallow | Medium | Haemulidae, Clupeidae, Mugilidae |
| \| **Atlantic Spotted Dolphin  (*Stenella plagiodon/frontalis*)** \| \| --- \| | 1.66 | 2.26 | 1.67 | 2.29 | Yes | Medium | Shallow | Medium | Gadidae, Clupeidae, Carangidae, Sciaenidae, Congridae, Trichiyridae, Triglidae |
| \| **Atlantic White-Sided Dolphin (*Lagenorhynchus acutus*)** \| \| --- \| | 2.44 | 2.75 | 1.94 | 2.43 | Yes | Medium | Shallow | Medium | Loliginidae, Ammodytidae, Osmeridae, Scombridae |
| \| **Black Dolphin**  **(*Cephalorhynchus eutropia*)** \| \| --- \| | 1.24 | 1.65 | 1.23 | 1.61 | Yes | Small | Shallow | Medium | Munididae, Loliginidae |
| \| **Bottlenose Dolphin  (Tursiops truncatus)** \| \| --- \| | 2.02 | 3.81 | 1.9 | 3.67 | Yes | Large | Medium | Medium | Engraulidae, Apongidae, Trichiuridae, Synodontidae, Scaridae, Haemulidae, Merluccidae, Serranidae, Clupeidae, Gadidae, Sparidae, Ophiidae, Congridae, Cepolidae, Caraengidae, Octopodidae, Loliginidae, Ommastrephidae, Sepiolidae, Sepiidae, Alphaeidae, Penaeideae, Grapsidae, Ophichthidae, Gerreidae, Mugilidae, Congiopodidae, Elopidae, Batrachoididae, Sciaenidae |
| \| **Clymene Dolphin  (*Stenella clymene*)** \| \| --- \| | 1.76 | 1.97 | 1.71 | 1.83 | Yes | Medium | Deep | Social | Myctophidae |
| \| **Commerson's Dolphin**  **(*Cephalorhynchus commersonii*)** \| \| --- \| | 1.3 | 1.67 | 1.39 | 1.74 | Yes | Small | Shallow | Solitary | Merlucciidae, Atherinopsidae, Lithodidae, Loliginidae, Rhodomelaceae, Laminariaceae, Sphacelariaceae , Ceramiaceae, Sertulariidae, Nereidae, Mysidae, Euphausiidae, Sphaeromatidae, Diastylidae, Ophiomixidae, Styelidae, Clupeidae, Gnathiidae, Sphaeromatidae, Cirolanidae, Pyuridae |
| Short Beaked Common Dolphin (*Delphinus delphis*) | 1.71 | 2.6 | 1.67 | 2.44 | Yes | Medium | Medium | Medium | Argentinidae, Bathylagidae, Batrachoididae, Melamphaidae, Scomberesocidae, Myctophidae, Sciaenidae, Engraulidae, Merlucciidae, Ophidiidae, Scombridae, Stromateidae, Loliginidae, Onychoteuthidae |
| \| **Dusky Dolphin  (*Lagenorhynchus obscurus*)** \| \| --- \| | 1.67 | 2.11 | 1.67 | 2.05 | No | Medium | Shallow | Medium | Engraulidae |
| \| **False Killer Whale  (*Pseudorca crassidens*)** \| \| --- \| | 3.96 | 6.1 | 3.4 | 5.06 | Yes | Large | Deep | Medium | Lycoteuthidae, Ommastrephidae, Scombridae, Coryphaenidae, Sciaenidae, Lateolabracidae, Salmonidae, Ariidae, Gadidae, Gonatidae, Cranchiidae |
| \| **Fraser's Dolphin  (*Lagenodelphis hosei*)** \| \| --- \| | 2.31 | 2.7 | 2.06 | 2.64 | Yes | Large | Deep | Social | Onychoteuthidae, Ophichthyidae, Scomberoscocidae, Octopoteuthidae, Diretmidae, Gempylidae, Acropomatidae, Macrouridae, Bregmacerotidae, Melamphidae, Myctophidae, Neoscopelidae, Paralepididae, Scopelarhidae, Sparidae, Trichiuridae, Nomeidae, Argentinidae, Bathylagidae, Gonostomatidae, Sternoptychidae, Stomiidae, Enoploteuthidae, Lycoteuthidae, Histioteuthidae, Ctenopteridae, Brachioteuthidae, Ommastrephidae, Thysanoteuthidae, Chiroteuthidae, Mastigoteuthidae, Cranchiidae, Oplophoridae, Sergestidae |
| \| **Indo-Pacific Humpbacked  Dolphin (*Sousa chinensis*)** \| \| --- \| | 2 | 3.2 | 2.16 | 2.44 | Yes | Medium | Shallow | Medium | Mugilidae, Engraulidae, Pristigasteridae, Haemulidae, Sparidae, Callichthyidae, Sciaenidae, Trichiuridae |
| \| **Heaviside's Dolphin  (*Cephalorhynchus heavisidii*)** \| \| --- \| | 1.51 | 1.74 | 1.59 | 1.66 | No | Small | Shallow | Solitary | Merlucciidae, Ophidiidae, Gobiinae, Loliginidae |
| \| **Hector's Dolphin  (*Cephalorhynchus hectori*)** \| \| --- \| | 1.17 | 1.38 | 1.28 | 1.53 | Yes | Small | Shallow | Solitary | Mugilidae, Uranoscopidae, Moridae, Ommastrephidae, Arripidae |
| \| **Hourglass Dolphin  (*Lagenorhynchus cruciger*)** \| \| --- \| | 1.63 | 1.87 | 1.66 | 1.83 | No | Small | Deep | Medium | Mycophidae |
| \| **Irrawaddy Dolphin  (*Orcaella brevirostris*)** \| \| --- \| | 2.2 | 2.35 | 2 | 2.32 | Yes | Small | Shallow | Solitary | Cyprinidae, Teraponidae, Apogonidae, Chirocentridae, Pangasiidae, Cuttlefish, Engraulidae, Clupeidae, Synodontidae, Hemirhamphidae, Psettodidae, Leigignathidae, Nemipteridae, Pomadasyidae, Sillaginidae, Platycephalidae |
| \| **Killer Whale  (*Orcinus orca*)** \| \| --- \| | 5.2 | 9.75 | 4.57 | 8.53 | Yes | Large | Medium | Medium | Eschrichtiidae, Ohyseteridae, Balaenopteridae, Balaenidae, Phocidae, Phocoenidae, Delphinidae, Monodontidae, Ziphiidae, Dugongidae, Otariidae, Odobenidae, Mustelidae, Salmonidae, Gadidae, Alces alces, Dasyatidae, Myliobatidae, Lamnidae, Sebastidae, Anoplopomatidae, Pleuronectidae, Centrolophidae, Clupidae, Torpedinidae, Triakidae, Carcharhinidae, Cetorhinidae, Cervidae |
| Long Beaked  Common Dolphin (*Delphinus capensis*) | 2 | 2.4 | 1.9 | 2.2 | Yes | Small | Shallow | Social | Engraulidae, Myctophidae, Phosichthyidae, Atherinopsidae, Meriuccidae, Clupeidae, Centrolophidae, Carangidae, Scombridae, Triglidae, Scomberesocidae, Sphyraenidae, Normanichthyidae, Batrachoididae, Ophichthidae, Galatheidae |
| Long-Finned Pilot Whale (*Globicephala melaena*) | 5 | 6.1 | 4.05 | 4.72 | Yes | Large | Medium | Medium | Octopodidae, Gonatidae, Ommastreohidae, Mastigoteuthidae, Loligoinidae, Argentinidae, Gadidae, Pleuronectidae, Macrouridae, Zoarcidae, Ammoditidae, Trichiuridae, Pandalidae, Galatheidae, Chiroteuthidae, Brachioteuthidae, Sepiolidae, Cranchiidaae, Histioteuthidae |
| \| **Melon-Headed Whale  (*Peponocephala electra*)** \| \| --- \| | 2.05 | 2.64 | 2.11 | 2.57 | Yes | Medium | Deep | Social | Ommastrephidae, Histioteuthidae, Loliginidae, Onycoteuthidae, Chiroteuthidae, Mastigoteuthidae, Cranchiidae, Enoploteruthidae, Myctophidae, Paralepididae, Scopelarchidae |
| \| **Northern Right Whale  Dolphin (*Lissodelphis borealis*)** \| \| --- \| | 2.11 | 3.1 | 1.95 | 2.3 | Yes | Medium | Deep | Social | Gonatidae, Onychoteuthidae, Loligindae, Enoploteuthidae, Histioteuthidae, Centrolophidae, Melamphaidae, Scomberesocidae, Merlucciidae, Myctophidae, Bathylagidae, Paralepididae |
| \| **Pacific White-Sided Dolphin  (*Lagenorhynchus obliquidens*)** \| \| --- \| | 1.7 | 2.5 | 1.7 | 2.36 | Yes | Medium | Medium | Medium | Engraulidae, Merlucciidae, Loliginidae |
| \| **Peale's Dolphin  (*Lagenorhynchus australis*)** \| \| --- \| | 1.6 | 2.18 | 1.63 | 2.1 | No | Small | Shallow | Solitary | Myxinidae, Ommastrephidae, Clupeidae, Moridae, Merlucciidae, Octopodidae, Loliginidae |
| \| **Pygmy Killer Whale  (*Feresa attenuata*)** \| \| --- \| | 2.07 | 2.59 | 2.07 | 2.45 | No | Large | Medium | Medium | Delphinidae |
| \| **Risso's Dolphin  (*Grampus griseus*)** \| \| --- \| | 2.53 | 3.6 | 2.4 | 3 | No | Small | Medium | Medium | Sepiidae, Brachioteuthidae, Cranchiidae, Mastigoteuthidae, Onychoteuthidae, Histioteuthidae, Ommastrephidae, Argonautidae, octopodidae, Ascidiacea, Pyrosomidae, Salpidae, Enoploteuthidae, Chiroteuthidae, Loliginidae, Sepiolidae |
| \| **Rough-Toothed Dolphin  (*Steno bredanensis*)** \| \| --- \| | 2.09 | 2.65 | 2.12 | 2.55 | Yes | Large | Medium | Medium | Tremoctopodidae, Onychoteuthidae, Coryphaenidae, Trichiuridae, Atherinopsidae, Scomberesocidae, Belonidae, Atherinopsidae, Centriscidae, Coryphaenidae, Ommastrephidae, Atherinidae, Loliginidae |
| \| **Short-Finned Pilot Whale  (*Globicephala macrorhynchus*)** \| \| --- \| | 4.24 | 4.91 | 3.34 | 3.92 | Yes | Medium | Medium | Social | Octopodidae, Enoploteuthidae, Histioteuthidae, Loliginidae, Mastigoteuthidae, Chiroteuthidae, Cranciidae, Brachioteuthidae, Lepidoteuthidae, Ommastrephidae, Melamphaidae |
| \| **Southern Right Whale Dolphin (*Lissodelphis peronii*)** \| \| --- \| | NA | NA | NA | NA | Yes | Medium | Medium | Social | Ommastrephidae, Mastigoteuthidae, Cranchiidae, Gonatidae, Bathylagidae, Photichthyidae, Myctophidae, Merlucciidae, Engraulididae |
| \| **Spinner Dolphin  (*Stenella longirostris*)** \| \| --- \| | 1.36 | 2.35 | 1.29 | 2.04 | Yes | Large | Deep | Social | Mastigotruthidae, Congridae, Stomiidae, Paralepididae, Bathylagidae, Ophichthyidae, Diretmidae, Melamphidae, Bregmacerotidae, Centrolophidae, Macrouridae, Neoscopelidae, Acropomatidae, Sparidae, Gempylidae, Exocoetidae, Gonostomatidae, Myctophidae, Scopelarchidae, Trichiuridae, Nomeidae, Argentinidae, Oplophoridae, Penaeidae, Sergestidae, Enoploteuthidae, Octopoterthidae, Onychoteuthidae, histioteuthidae, Brachioteuthidae, Ommastrephidae, Chiroteuthidae, Cranchiidae |
| Pan tropical Spotted Dolphin (*Stenella attenuata*) | 1.66 | 2.57 | 1.63 | 1.63 | Yes | Large | Deep | Social | NA |
| \| **Striped Dolphin  (*Stenella coeruleoalba*)** \| \| --- \| | 2.15 | 2.56 | 1.85 | 2.36 | Yes | Large | Deep | Medium | Ateleopodidae, Myctophidae, Microstomatidae, Melamphaeidae, Bathylagidae, Gempylidae, Brachioteuthidae, Cranchiidae, Cycloteuthidae, Enoploteuthidae, Grimalditeuthidae, Histioteuthidae, Mastigoteuthidae, Octopoteuthidae, Ommastrephidae, Onychoteuthidae, Pholidoteuthidae, Tremoctopodidae, Sternoptychidae, Nomeidae, Paralepididae, Phosichthyidae, Scopelarchidae |
| \| **Guiana dolphin  (*Sotalia guianensis*)** \| \| --- \| | 1.31 | 1.87 | 1.38 | 2.06 | No | Small | Shallow | Solitary | Clupeidae, Scianidae, Batrachoididae, Trichiuridae, Loliginidae |
| \| **White-Beaked Dolphin  (*Lagenorhynchus albirostris*)** \| \| --- \| | 2.51 | 3.1 | 1.74 | 2.78 | Yes | Medium | Shallow | Medium | Gadidae, Clupeidae, Osmeridae |
| \| **Beluga  (*Delphinapterus leucas*)** \| \| --- \| | 3.5 | 4.7 | 3.1 | 3.9 | Yes | Medium | Medium | Solitary | Osmeridae, Clupeidae, Ammodytidae, Cyclopteridae, Characidae, Osmeridae, Salmonidae, Gadidae, Illicinae |
| \| **Narwhal  (*Monodon monoceros*)** \| \| --- \| | 4.1 | 4.7 | 3.4 | 4.15 | Yes | Small | Deep | Solitary | Gadidae, Pleuronectidae, Salmonidae, Clupeidae |
| \| **Franciscana  (*Pontoporia blainvillei*)** \| \| --- \| | 1.21 | 1.58 | 1.37 | 1.74 | Yes | Small | Shallow | Medium | Gobiidae, Penaeidae, Ophidiiae, Cynoglossidae, Trichiuridae, Carangidae, Phycidae, Antherinidae, Poatmidae, Engraulidae, Sciaenidae, Loliginidae, Batrachoidiae, Gadidae, Stromatidae, Congridae, Stromateidae, Nomeidae |
| \| **Burmeister's Porpoise  (*Phocoena spinipinnis*)** \| \| --- \| | 1.51 | 1.75 | 1.53 | 1.85 | Yes | Small | Shallow | Solitary | Merlucciidae, Engraulidae, Loliginidae, Euphausiidae, Centrolophidae, Carangidae, Clupeidae, Congridae, Centropomidae, Atherinopsidae, Sciaenidae, Gadidae, Sparidae |
| Cochito [Vaquita]  (*Phocoena sinus*) | 1.27 | 1.44 | 1.35 | 1.48 | Yes | Small | Shallow | Solitary | Sciaenidae, Haemulidae |
| \| **Dall's Porpoise  (*Phocoenoides dalli*)** \| \| --- \| | 1.75 | 1.8 | 1.74 | 1.77 | Yes | Medium | Medium | Medium | Bolitaenidae, Enoploteuthidae, Gonatidae, Paralepididae, Opisthoproctidae, Scombridae, Clupeidae, Onychoteuthidae, Loliginidae, Carangidae, Osmeridae, Merlucciidae, Scomberesocidae, Engraulidae, Sebastidae, Myctophidae, Anomalopidae, Cranchiidae, Salmonidae, Paralichthyidae, Sepiolidae, Ommastrephidae, Octopoteuthidae, Histioteuthidae, Scopelarchidae, Pleuronectidae, Ophidiidae, Stromateidae, Gadidae, Lotidae, Macrouridae, Ammodytidae, Anoplopomatidae, Melamphaidae, Hexagrammidae |
| \| **Finless Porpoise  (*Neophocaena phocaenoides*)** \| \| --- \| | 1.32 | 2.27 | 1.32 | 2.06 | Yes | Medium | Shallow | Solitary | Loliginidae, Apogonidae, Leiognathidae, Sepiidae, Engraulidae |
| \| **Harbour Porpoise (*Phocoena phocoena*)** \| \| --- \| | 1.23 | 1.6 | 1.38 | 1.7 | Yes | Medium | Shallow | Solitary | Octopodidae, Clupeidae, Gadidae, Gobiidae, Anguillidae, Gonostomatidae, Marlucciidae, Sparidae, Zoarcidae, Ammodytidae, Pleuronectidae, Sebastidae, Illicinae, Sternoptychidae, Merlucciidae, Stromateidae, Scombridae, Sepiolidae, Myxinidae, Loliginidae |
| \| **Spectacled Porpoise (*Phocoena dioptrica*)** \| \| --- \| | 1.89 | 2.24 | 1.74 | 2.04 | Yes | Small | Deep | Solitary | Engrauliidae |
| \| **Gray whale (*Eschrichtius robustus*)** \| \| --- \| | 11.1 | 14.6 | 11.7 | 15 | Yes | Small | Shallow | Solitary | Pachychilidae, Pinnotheridae, Galatheidae, Nephropidae, Atylidae, Ampeliscidae |
| \| **Blue Whale  (*Balaenoptera musculus*)** \| \| --- \| | 20 | 25 | 21 | 33.6 | Yes | Large | Medium | Solitary | Euphausiidae, Temoridae |
| \| **Bryde's Whale**  **(*Balaenoptera edeni*)** \| \| --- \| | 12 | 14.2 | 13.7 | 15.5 | Yes | Medium | Medium | Solitary | Clupeidae, Engraulidae, Euphausiidae, Carangidae |
| \| **Fin Whale**  **(*Balaenoptera physalus*)** \| \| --- \| | 17.7 | 25 | 18.3 | 27 | Yes | Large | Medium | Medium | Ommastrephidae, Euphausiidae, Osmeridae, Calanidae, Clupeidae, Gadidae |
| Antarctic Minke Whale (*Balaenoptera bonaerensis*) | 7.3 | 8.6 | 7.9 | 9 | Yes | Medium | Deep | Solitary | Channichthyidae, Myctophidae, Paralepididae, Nototheniidae, Euphausiidae, Hyperiidae |
| Common Minke Whale (*Balaenoptera acutorostrata*) | 6.7 | 8.2 | 7.2 | 8.8 | Yes | Medium | Shallow | Solitary | Euphausiidae, Gadidae, Salmonidae, Ammodytidae, Clupeidae, Osmeridae, Scombridae, Anarhichadidae, Squalidae, Merlucciidae |
| \| **Sei Whale**  **(*Balaenoptera borealis*)** \| \| --- \| | 12.8 | 15.9 | 13.3 | 16.1 | Yes | Large | Deep | Solitary | Clausocalanidae, Hyperiidae, Temoridae, Euphausiidae, Eucalanidae, Metridinidae, Calanidae, Engraulidae, Clupeidae, Scomberesocidae, Myctophidae |
| \| **Humpback Whale**  **(*Megeptera novaeangliae*)** \| \| --- \| | 12 | 14.8 | 13.9 | 15.5 | Yes | Large | Deep | Solitary | Mysidae, Euphausiidae, Pandalidae, Clupeidae, Osmeridae, Gadidae |
| \| **Bowhead Whale**  **(*Balaena mysticetus*)** \| \| --- \| | 11.6 | 15.5 | 14 | 18 | Yes | Small | Medium | Solitary | Euphausiidae, Calanidae |
| North Pacific Right Whale (*Eubalaena japonica*) | 15 | 17.1 | 15.5 | 18.3 | Yes | Medium | Shallow | Solitary | Euphausiidae, Calanidae |
| North Atlantic Right Whale (*Eubalaena glacialis*) | 11 | 12.9 | 11 | 18 | Yes | Small | Shallow | Solitary | Euphausiidae, Calanidae |
| \| **Southern Right Whale  (*Eubalaena australis*)** \| \| --- \| | 11.3 | 15.2 | 12.3 | 16.5 | Yes | Small | Shallow | Solitary | Euphausiidae, Calanidae |
| \| **Pygmy Right Whale  (*Caperea marginata*)** \| \| --- \| | 5.47 | 6.09 | 6 | 6.45 | Yes | Small | NA | Solitary | Paracalanidae, Centropagidae, Calanidae, Acartiidae, Clausocalanidae, Onceidae, Oithonidae, Hyperiidae |
| Indo-pacific bottlenose dolphin (*Tursiops aduncus*) | 2.09 | 2.43 | 2.01 | 2.38 | No | Medium | Shallow | Medium | Apogonidae, Leiognathidae, Lethrinidae, Cynnoglossidae, Congridae, Clupeidae, Carangidae, Argentinidae, Chlorophthalmidae, Citharoidea, Dactylopteridae, Gerreidae, Gempylidae, Gobiidae, Haemulidae, Holocentridae, Mugiloididae, Monacanthidae, Lutjanidae, Muraenesidae, Muraenidae, Myctophidae, Nemipteridae, Opichthidae, Ophidiidae, Platycephalidae, Pomacanthidae, Pomacentridae, Scorpaenidae, Serranidae, sparidae, Sternoptychidae, Synaphobranchidae, Trichiuridae, Synodontidae |
| Sperm Whale  (*Physeter macrocephalus*) | 15.2 | 183 | 10.4 | 12.5 | Yes | Large | Deep | Solitary | Ceratiidae, Gadidae, Macrouridae, Trachipteridae, Icosteidae, Scorpaenidae, Anoplomatidae, Hexagrammida, Nototheniidae, Gnathophausia, Majidae, Cancridae, Architeuthis, Ommastrephidae, Onychoteuthidae, Gonatidae, Pholidoteuthidae, Octopoteuthidae, Histipteuthidae, Cranchiidae, Vampyroteuthidae, Octopodidae |
| Pygmy Sperm Whale  (*Kogia breviceps*) | 2.7 | 3.3 | 2.66 | 3.3 | No | Medium | Deep | Solitary | Cranchiidae, Enoploteuthidae, Histioteuthidae, Lycoteuthidae, Ommastrephidae, Myctophidae, Gadidae, Gempylidae |
| Dwarf Sperm Whale  (*Kogia sima*) | 2.19 | 2.34 | 2.1 | 2.34 | No | Medium | Deep | Solitary | Ommastrephidae, Cranchiidae, Onychoteuthidae, Lycoteuthidae, Enoploteuthidae, Octopoteuthidae, Chiroteuthidae, Vampyroteuthidae, Gonatidae, Gonostomatidae, Macrouridae, Sternoptychidae, Congridae, Argentinidae, Loligiginidae, Sepiidae, Histioteuthidae, Octopodidae, Moridae, Myctophidae, Penaeidae, Acanthephyridae, Aristeidae, Argentinidae, Microstomatidae |

| Species | Predators | Min Temp (°C) | Max Temp (°C) | Vocalization Frequency | Parasites | Species Known to Associate with | References (and sources cited within) |
| --- | --- | --- | --- | --- | --- | --- | --- |
| \| **Andrew's Beaked Whale (*Mesoplodon bowdoini*)** \| \| --- \| | Dalatiidae | 13.0 | 19 | Medium | NA | NA | 1, 3, 12, 202, 339 |
| \| **Arnoux's Beaked Whale**  **(*Berardius arnuxii*)** \| \| --- \| | Dalatiidae | NA | NA | Medium | NA | Killer Whale (Orcinus orca), Hourglass Dolphin (Lagenorhynchus cruciger), Peale's Dolphin (Lagenorhynchus australis) | 2, 9, 12, 46, 101, 200, 204, 258, 340 |
| Baird's Beaked Whale (*Berardius bairdii*) | Dalatiidae, Delphinidae | 15.0 | 29 | Medium | Cyamidae, Lepadidae, Anisakidae, Tetrameridae, Monocotylidae, Phyllobothriidae, Diphyllobothriidae, Brachycladiidae, Cestodes, Trematodes, Nematodes | Northern Right Whale Dolphin (Lissodelphis borealis) | 7, 8, 9, 12, 101, 200, 340 |
| \| **Blainville's Beaked Whale**  **(*Mesoplodon* *densirostris*)** \| \| --- \| | Dalatiidae | 25.0 | 29 | Medium | Anisakidae, Tetrabothriidae, Lepadidae | NA | 4, 9, 11, 12, 69, 207, 258, 319, 339 |
| \| **Cuvier's Beaked Whale**  **(*Ziphius cavirostris*)** \| \| --- \| | Dalatiidae, Lamnidae, Delphinidae | 25.0 | 29 | High | Phyllobothriidae, Tetrameridae | NA | 9, 10, 11, 12, 79, 103, 131, 201, 207, 258, 304, 319, 338 |
| \| **Gervais' Beaked Whale  (*Mesoplodon europaeus*)** \| \| --- \| | Dalatiidae | NA | NA | Medium | Lepadidae, Phyllobothriidae, Cyamidae | NA | 9, 12, 69, 71, 258, 314, 339 |
| \| **Ginkgo-Toothed Beaked Whale (*Mesoplodon* *ginkgodens*)** \| \| --- \| | Dalatiidae | NA | NA | Medium | NA | NA | 9, 12, 69, 258, 339 |
| \| **Gray's Beaked Whale**  **(*Mesoplodon* *grayi*)** \| \| --- \| | Dalatiidae | NA | NA | Medium | NA | NA | 9, 12, 46, 69, 71, 258, 339 |
| \| **Hector's Beaked Whale**  **(*Mesoplodon* *hectori*)** \| \| --- \| | Dalatiidae | NA | NA | Medium | Lepadidae, Phyllobothriidae | NA | 6, 9, 12, 46, 69, 258, 339 |
| \| **Hubb's Beaked Whale**  **(*Mesoplodon* *carlhubbsi*)** \| \| --- \| | Dalatiidae | NA | NA | Medium | Pennellidae | NA | 1, 5, 12, 69, 339 |
| Pygmy Beaked whale (*Mesoplodon* *peruvianus*) | NA | 18.2 | 19.3 | Medium | Anisakidae, Campulidae | NA | 12, 68, 69, 71, 312, 313, 339 |
| \| **Longman's Beaked Whale**  **(*Mesoplodon* *pacificus*)** \| \| --- \| | Dalatiidae | 27.0 | 30 | Medium | NA | Short-Finned Pilot Whale (Globicephala macrorhynchus), Bottlenose Dolphin (Tursiops truncatus), Spinner Dolphin (Stenella longirostris) | 12, 34, 69, 80, 309, 339 |
| \| **Northern Bottlenose Whale**  **(*Hyperoodon* *ampullatus*)** \| \| --- \| | Delphinidae | -1.3 | -0.9 | Medium | Tetrabothriidae, Anisakidae, Polymorphidae | Killer Whale (Orcinus orca) | 9, 12, 46, 49, 103, 126, 300, 304, 336, 339 |
| \| **Southern Bottlenose Whale**  **(*Hyperoodon planifrons*)** \| \| --- \| | Dalatiidae | NA | NA | Medium | Tetrameridae | Killer Whale (Orcinus orca), Peale's Dolphin (Lagenorhynchus australis), Hourglass Dolphin (Lagenorhynchus cruciger) | 9, 12, 46, 70, 126, 258, 336, 339 |
| \| **Sowerby's Beaked Whale**  **(*Mesoplodon* *bidens*)** \| \| --- \| | Dalatiidae | NA | NA | Medium | Phyllobothriidae, Tetrabothriidae, Lepadidae, Tetrameridae, Anisakidae, Polymorphidae | NA | 9, 12, 46, 69, 71, 103, 173, 258, 339 |
| \| **Straptoothed Whale**  **(*Mesoplodon* *layardii*)** \| \| --- \| | Dalatiidae | 10.0 | 16 | Medium | Lepadidae | NA | 9, 12, 46, 67, 69, 81, 258, 339 |
| \| **Stejneger's Beaked Whale  (*Mesoplodon* *stejnegeri*)** \| \| --- \| | Dalatiidae, Lamnidae | NA | NA | Medium | Tetrameridae, Tetrabothriidae | NA | 9, 12, 69, 71, 201, 258, 310, 319, 339 |
| \| **Tasman Beaked Whale  (*Tasmacetus shepherdi*)** \| \| --- \| | NA | 13.0 | 19 | NA | NA | NA | 9, 12, 102, 123, 202, 308 |
| \| **True's Beaked Whale  (*Mesoplodon mirus*)** \| \| --- \| | Dalatiidae | 13.0 | 19 | Medium | Coronulidae, Pennellidae, Anisakidae | NA | 9, 12, 69, 71, 174, 202, 258, 315, 339 |
| \| **Atlantic Humpbacked Dolphin  (*Sousa teuszii*)** \| \| --- \| | NA | 18.8 | 24.5 | High | Cyamidae | Bottlenose Dolphin (Tursiops truncatus) | 25, 37, 137, 187, 262, 285, 306, 307, 340 |
| \| **Atlantic Spotted Dolphin  (*Stenella plagiodon/frontalis*)** \| \| --- \| | NA | 19.0 | 27 | High | Lepadidae, Coronulidae, Cyamidae, Echeneidae, Campulidae, Heterophyidae, Brauninidae, Pseudaliidae, Anisakidae | Rough-Toothed Dolphin (Steno bredanensis), Bottlenose Dolphin (Tursiops truncatus), Risso's Dolphin (Grampus griseus), Pan tropical Spotted Dolphin (Stenella attenuata) | 13, 16, 38, 46, 94, 212, 217, 340, 341, 345 |
| \| **Atlantic White-Sided Dolphin (*Lagenorhynchus acutus*)** \| \| --- \| | NA | 4.0 | 10 | High | Heterophyidae, Brachycladiidae, Tetrabothriidae, Phyllobothriidae, Tetrabothriidae, Pseudaliidae , Tetrameridae, Pseudaliidae, Polymorphidae, Pseudaliidae, Heterophyidae | Killer Whale (Orcinus orca), Bottlenose Dolphin (Tursiops truncatus), Short Beaked Common Dolphin (Delphinus delphis), Long-Finned Pilot Whale (Globicephala melas), White-Beaked Dolphin (Lagenorhynchus albirostris), Fin Whale (Balaenoptera physalus), Humpback Whale (Megeptera novaeangliae) | 17, 21, 26, 27, 49, 51, 50, 51, 55, 340, 344, 346 |
| \| **Black Dolphin**  **(*Cephalorhynchus eutropia*)** \| \| --- \| | NA | 11.0 | 14.6 | Low | Anisakidae, Brauninidae, Campulidae, Polymorphidae | Peale's Dolphin (Lagenorhynchus australis), Commerson's Dolphin (Cephalorhynchus commersonii) | 17, 46, 60, 111, 170, 188, 348 |
| \| **Bottlenose Dolphin  (*Tursiops truncatus*)** \| \| --- \| | Dalatiidae, Carcharhinidae, Lamnidae, Hexanchidae | 15.0 | 29 | High | Brauninidae, Nasitrematidae, Heterophyidae, Anisakidae, Brachycladiidae, Phyllobothriidae, Campulidae, Tetrameridae, Diphyllobothriidae, Pseudaliidae, Polymorphidae | Guiana dolphin (Sotalia guianensis), Atlantic Spotted Dolphin (Stenella plagiodon/frontalis), Melon-Headed Whale (Peponocephala electra), Short-Finned Pilot Whale (Globicephala macrorhynchus), Atlantic Humpbacked Dolphin (Sousa teuszii), Longman's Beaked Whale (Mesoplodon pacificus), Atlantic White-Sided Dolphin (Lagenorhynchus acutus), Humpback Whale (Megeptera novaeangliae), Common Minke Whale (Balaenoptera acutorostrata), Harbour Porpoise (Phocoena phocoena), Burmeister's Porpoise (Phocoena spinipinnis), Pan tropical Spotted Dolphin (Stenella attenuata), Southern Right Whale Dolphin (Lissodelphis peronii), Risso's Dolphin (Grampus griseus), Hourglass Dolphin (Lagenorhynchus cruciger), Killer Whale (Orcinus orca), Northern Right Whale Dolphin (Lissodelphis borealis), Pacific White-Sided Dolphin (Lagenorhynchus obliquidens), Rough-Toothed Dolphin (Steno bredanensis), Gray whale (Eschrichtius robustus), White-Beaked Dolphin (Lagenorhynchus albirostris), False Killer Whale (Pseudorca crassidens), Indo-Pacific Humpbacked Dolphin (Sousa chinensis), Sperm Whale (Physeter macrocephalus), Blue Whale (Balaenoptera musculus) | 13, 15, 17, 154, 158, 159, 160, 186, 187, 207, 246, 265, 266, 319, 322, 326, 340, 341 |
| \| **Clymene Dolphin  (*Stenella clymene*)** \| \| --- \| | Dalatiidae | 20.2 | 28.5 | High | Coronulidae, Cyamidae, Pseudaliidae, Phyllobothriidae, Nasitrematidae | Pan tropical Spotted Dolphin (Stenella attenuata), Short Beaked Common Dolphin (Delphinus delphis), Melon-Headed Whale (Peponocephala electra) | 13, 16, 61, 62, 130, 169, 172, 212, 217, 340, 341, 345 |
| \| **Commerson's Dolphin**  **(*Cephalorhynchus commersonii*)** \| \| --- \| | NA | 1.0 | 16 | Low | Brachycladiidae, Anisakidae, Brauninidae, Heterophyidae, Tetrabothriidae | Peale's Dolphin (Lagenorhynchus australis), Black Dolphin (Cephalorhynchus eutropia), Burmeister's Porpoise (Phocoena spinipinnis) | 17, 46, 108, 109, 110, 185, 200, 348 |
| Short Beaked Common Dolphin (*Delphinus delphis*) | Dalatiidae, Carcharhinidae, Lamnidae, Delphinidae, Sphyrnidae | 7.0 | 23 | High | Anisakidae, Pseudaliidae, Tetrameridae, Phyllobothriidae, Campulidae, Heterophyidae | Striped Dolphin (Stenella coeruleoalba), Risso's Dolphin (Grampus griseus), Clymene Dolphin (Stenella clymene), Atlantic White-Sided Dolphin (Lagenorhynchus acutus), Northern Right Whale Dolphin (Lissodelphis borealis), Pacific White-Sided Dolphin (Lagenorhynchus obliquidens), Southern Right Whale Dolphin (Lissodelphis peronii), Gray whale (Eschrichtius robustus), Dusky Dolphin (Lagenorhynchus obscurus), Risso's Dolphin (Grampus griseus), Long-Finned Pilot Whale (Globicephala melas), Spinner Dolphin (Stenella longirostris) | 15, 17, 23, 24, 27, 94, 95, 124, 143, 149, 201, 202, 205, 246, 258, 318, 319, 340, 341 |
| \| **Dusky Dolphin  (*Lagenorhynchus obscurus*)** \| \| --- \| | Hexanchidae, Delphinidae, Lamnidae | 10.4 | 19 | High | Tetrabothriidae, Phyllobothriidae, Anisakidae, Trematodes, Pseudaliidae, Brauninidae | Southern Right Whale Dolphin (Lissodelphis peronii), Short Beaked Common Dolphin (Delphinus delphis), Long-Finned Pilot Whale (Globicephala melas), Killer Whale (Orcinus orca), Risso's Dolphin (Grampus griseus), Burmeister's Porpoise (Phocoena spinipinnis), Heaviside's Dolphin (Cephalorhynchus heavisidii) | 17, 46, 49, 50, 51, 55, 82, 201, 202, 205, 304, 319, 340, 344, 346 |
| \| **False Killer Whale  (*Pseudorca crassidens*)** \| \| --- \| | Dalatiidae, Delphinidae | 9.0 | 31 | Medium | Anisakidae, Pseudaliidae, Polymorphidae, Brachycladiidae, Nasitrematidae, Cyamidae, Coronulidae | Killer Whale (Orcinus orca), Indo-pacific bottlenose dolphin(Tursiops aduncus), Rough-Toothed Dolphin (Steno bredanensis), Fraser's Dolphin (Lagenodelphis hosei), Risso's Dolphin (Grampus griseus), Bottlenose Dolphin (Tursiops truncatus), Melon-Headed Whale (Peponocephala electra), Short-Finned Pilot Whale (Globicephala macrorhynchus) | 16, 41, 42, 84, 207, 225, 258, 319, 340 |
| \| **Fraser's Dolphin  (*Lagenodelphis hosei*)** \| \| --- \| | Dalatiidae | 25.0 | 29 | High | Tetrabothriidae, Phyllobothriidae, Tetrabothriidae, Brachycladiidae, Anisakidae, Pseudaliidae, Polymorphidae | Sperm Whale (Physeter macrocephalus), False Killer Whale (Pseudorca crassidens), Striped Dolphin (Stenella coeruleoalba), Pan tropical Spotted Dolphin (Stenella attenuata), Spinner Dolphin (Stenella longirostris), Short-Finned Pilot Whale (Globicephala macrorhynchus), Melon-Headed Whale (Peponocephala electra), Risso's Dolphin (Grampus griseus) | 16, 17, 96, 122, 132, 133, 134, 207, 340, 344 |
| \| **Indo-Pacific Humpbacked  Dolphin (*Sousa chinensis*)** \| \| --- \| | Lamnidae, Delphinidae | 23.9 | 29.6 | High | Anisakidae, Pseudaliidae, Cyamidae | Bottlenose Dolphin (Tursiops truncatus), Killer Whale (Orcinus orca), Southern Right Whale (Eubalaena australis), Long Beaked common Dolphin (Delphinus capensis) | 37, 187, 189, 262, 273, 306, 319, 340 |
| \| **Heaviside's Dolphin  (*Cephalorhynchus heavisidii*)** \| \| --- \| | Lamnidae, Hexanchidae | 9.0 | 19 | Low | Cyamidae, Coronulidae | Dusky Dolphin (Lagenorhynchus obscurus) | 17, 31, 200, 203 |
| \| **Hector's Dolphin  (*Cephalorhynchus hectori*)** \| \| --- \| | Hexanchidae, Carcharhinidae | 6.3 | 22 | Low | Cyamidae, Strigeidae, Brauninidae, Halocercinae, Pseudaliidae , Anisakidae, Acariidae, Polymorphidae, Campulidae, Phyllobothriidae | NA | 17, 97, 112, 129, 179, 200, 201, 319, 203 |
| \| **Hourglass Dolphin  (*Lagenorhynchus cruciger*)** \| \| --- \| | NA | -0.3 | 13.4 | High | Anisakidae | Fin Whale (Balaenoptera physalus), Antarctic Minke Whale (Balaenoptera bonaerensis), Common Minke Whale (Balaenoptera acutorostrata), Sei Whale (Balaenoptera borealis), Bottlenose Dolphin (Tursiops truncatus), Southern Right Whale Dolphin (Lissodelphis peronii), Long-Finned Pilot Whale (Globicephala melas), Arnoux's Beaked Whale (Berardius arnuxii), Southern Bottlenose Whale (Hyperoodon planifrons), Killer Whale (Orcinus orca) | 46, 49, 50, 51, 55, 58, 136, 205, 275, 340, 344, 346 |
| \| **Irrawaddy Dolphin  (*Orcaella brevirostris*)** \| \| --- \| | Carcharhinidae | 20.0 | 35 | Low | Trematode , Schistosomatidae, Nematode | Finless Porpoise (Neophocaena phocaenoides), Spinner Dolphin (Stenella longirostris) | 17, 40, 47, 139, 180, 253, 321, 340 |
| \| **Killer Whale  (*Orcinus orca*)** \| \| --- \| | Dalatiidae | 1.7 | 26 | Medium | Fasciolidae, Tetrabothriidae, Phyllobothriidae, Anisakidae | Southern Right Whale (Eubalaena australis), Sei Whale (Balaenoptera borealis), Fin Whale (Balaenoptera physalus), Bryde's Whale (Balaenoptera edeni), Blue Whale (Balaenoptera musculus), Gray whale (Eschrichtius robustus), Harbour Porpoise (Phocoena phocoena), Dall's Porpoise (Phocoenoides dalli), Narwhal (Monodon monoceros), Beluga (Delphinapterus leucas), White-Beaked Dolphin (Lagenorhynchus albirostris), Risso's Dolphin (Grampus griseus), Peale's Dolphin (Lagenorhynchus australis), Pacific White-Sided Dolphin (Lagenorhynchus obliquidens), Arnoux's Beaked Whale (Berardius arnuxii), Northern Bottlenose Whale (Hyperoodon ampullatus), Southern Bottlenose Whale (Hyperoodon planifrons), Atlantic White-Sided Dolphin (Lagenorhynchus acutus), Bottlenose Dolphin (Tursiops truncatus), Dusky Dolphin (Lagenorhynchus obscurus), False Killer Whale (Pseudorca crassidens), Indo-Pacific Humpbacked Dolphin, Long-Finned Pilot Whale (Globicephala melas), Humpback Whale (Megeptera novaeangliae), Hourglass Dolphin (Lagenorhynchus cruciger), Short-Finned Pilot Whale (Globicephala macrorhynchus) | 16, 17, 103, 149, 204, 205, 207, 225, 258, 292, 293, 294, 303, 304, 305, 306, 340 |
| Long Beaked  Common Dolphin (*Delphinus capensis*) | Dalatiidae | NA | NA | High | Anisakidae | Pacific White-Sided Dolphin (Lagenorhynchus obliquidens), Indo-Pacific Humpbacked Dolphin (Sousa chinensis), Bryde's Whale (Balaenoptera edeni) | 94, 95, 124, 156, 173, 184, 197, 258, 340, 341 |
| Long-Finned Pilot Whale (*Globicephala melaena*) | Delphinidae | 0.6 | 22 | High | Brachycladiidae, Phyllobothriidae, Diphyllobothriidae, Tetrabothriidae, Anisakidae, Pseudaliidae, Polymorphidae | Southern Right Whale Dolphin (Lissodelphis peronii), Humpback Whale (Megeptera novaeangliae), Killer Whale (Orcinus orca), Atlantic White-Sided Dolphin (Lagenorhynchus acutus), Hourglass Dolphin (Lagenorhynchus cruciger), Peale's Dolphin (Lagenorhynchus australis), White-Beaked Dolphin (Lagenorhynchus albirostris), Pygmy Right Whale (Caperea marginata), Dusky Dolphin (Lagenorhynchus obscurus), Short Beaked Common Dolphin (Delphinus delphis) | 15, 17, 24, 46, 85, 103, 115, 202, 205, 304, 343 |
| \| **Melon-Headed Whale  (*Peponocephala electra*)** \| \| --- \| | Dalatiidae | 25.0 | 29 | High | Nasitrematidae, Phyllobothriidae, Pseudaliidae, Anisakidae | Bottlenose Dolphin (Tursiops truncatus), Spinner Dolphin (Stenella longirostris), Pygmy Killer Whale (Feresa attenuata), Fraser's Dolphin (Lagenodelphis hosei), Humpback Whale (Megeptera novaeangliae), Short-Finned Pilot Whale (Globicephala macrorhynchus), Rough-Toothed Dolphin (Steno bredanensis), Clymene Dolphin (Stenella clymene), Pan tropical Spotted Dolphin (Stenella attenuata), False Killer Whale (Pseudorca crassidens), Sperm Whale (Physeter macrocephalus) | 16, 49, 63, 64, 65, 66, 103, 207, 258, 346 |
| \| **Northern Right Whale  Dolphin (*Lissodelphis borealis*)** \| \| --- \| | NA | 8.0 | 19 | High | Nasitrematidae, Tetrameridae, Anisakidae, Phyllobothriidae, Coronulidae, Pennellidae | Pacific White-Sided Dolphin (Lagenorhynchus obliquidens), Short Beaked Common Dolphin (Delphinus delphis), Bottlenose Dolphin (Tursiops truncatus), Risso's Dolphin (Grampus griseus), Short-Finned Pilot Whale (Globicephala macrorhynchus), Dall's Porpoise (Phocoenoides dalli), Baird's Beaked Whale (Berardius bairdii), Sperm Whale (Physeter macrocephalus), Fin Whale (Balaenoptera physalus), Gray whale (Eschrichtius robustus), Humpback Whale (Megeptera novaeangliae), Sei Whale (Balaenoptera borealis) | 36, 46, 178, 311, 347 |
| \| **Pacific White-Sided Dolphin  (*Lagenorhynchus obliquidens*)** \| \| --- \| | Delphinidae, Lamnidae | 12.0 | 13 | High | Nasitrematidae, Brachycladiidae, Phyllobothriidae, Tetrabothriidae, Anisakidae, Tetrameridae | Northern Right Whale Dolphin (Lissodelphis borealis), Killer Whale (Orcinus orca), Risso's Dolphin (Grampus griseus), Striped Dolphin (Stenella coeruleoalba), Short Beaked Common Dolphin (Delphinus delphis), Long Beaked common Dolphin (Delphinus capensis), Short-Finned Pilot Whale (Globicephala macrorhynchus), Bottlenose Dolphin (Tursiops truncatus), Sperm Whale (Physeter macrocephalus), Gray whale (Eschrichtius robustus), Blue Whale (Balaenoptera musculus), Fin Whale (Balaenoptera physalus), Sei Whale (Balaenoptera borealis), Humpback Whale (Megeptera novaeangliae), Harbour Porpoise (Phocoena phocoena), Dall's Porpoise (Phocoenoides dalli), Southern Right Whale Dolphin (Lissodelphis peronii) | 17, 46, 50, 51, 52, 53, 54, 55, 210, 317, 319, 340, 344, 346 |
| \| **Peale's Dolphin  (*Lagenorhynchus australis*)** \| \| --- \| | NA | 6.0 | 9 | High | Tetrabothriidae | Long-Finned Pilot Whale (Globicephala melas), Arnoux's Beaked Whale (Berardius arnuxii), Killer Whale (Orcinus orca), Southern Right Whale Dolphin (Lissodelphis peronii), Southern Bottlenose Whale (Hyperoodon planifrons), Black Dolphin (Cephalorhynchus eutropia), Antarctic Minke Whale (Balaenoptera bonaerensis), Fin Whale (Balaenoptera physalus), Sei Whale (Balaenoptera borealis), Risso's Dolphin (Grampus griseus), Commerson's Dolphin (Cephalorhynchus commersonii), Southern Right Whale (Eubalaena australis) | 50, 51, 55, 56, 57, 182, 183, 340, 344, 346 |
| \| **Pygmy Killer Whale  (*Feresa attenuata*)** \| \| --- \| | Dalatiidae | 25.0 | 29 | NA | Lepadidae, Anisakidae, Pseudaliidae, Tetrabothriidae, Nasitrematidae | Rough-Toothed Dolphin (Steno bredanensis), Melon-Headed Whale (Peponocephala electra), Risso's Dolphin (Grampus griseus) | 16, 48, 207, 210, 258, 316, 319 |
| \| **Risso's Dolphin  (*Grampus griseus*)** \| \| --- \| | Dalatiidae, Lamnidae | 7.5 | 35 | Medium | Phyllobothriidae, Tetrabothriidae, Tetrameridae, Pseudaliidae, Anisakidae, Nasitrematidae, Cyamidae, Coronulidae | Bottlenose Dolphin (Tursiops truncatus), Fin Whale (Balaenoptera physalus), Sperm Whale (Physeter macrocephalus), Short-Finned Pilot Whale (Globicephala macrorhynchus), Short Beaked Common Dolphin (Delphinus delphis), Northern Right Whale Dolphin (Lissodelphis borealis), Dall's Porpoise (Phocoenoides dalli), Striped Dolphin (Stenella coeruleoalba), Killer Whale (Orcinus orca), Pan tropical Spotted Dolphin (Stenella attenuata), False Killer Whale (Pseudorca crassidens), Pygmy Killer Whale (Feresa attenuata), Short Beaked Common Dolphin (Delphinus delphis), Fraser's Dolphin (Lagenodelphis hosei), Pacific White-Sided Dolphin (Lagenorhynchus obliquidens), Dusky Dolphin (Lagenorhynchus obscurus), Atlantic Spotted Dolphin (Stenella plagiodon/frontalis), Peale's Dolphin (Lagenorhynchus australis), Gray whale (Eschrichtius robustus), Rough-Toothed Dolphin (Steno bredanensis) | 13, 16, 44, 59, 83, 200, 205, 210, 258, 316, 319 |
| \| **Rough-Toothed Dolphin  (*Steno bredanensis*)** \| \| --- \| | Dalatiidae, Delphinidae | 25.0 | 29 | Medium | Tetrabothriidae, Polymorphidae, Anisakidae, Cyamidae | False Killer Whale (Pseudorca crassidens), Bottlenose Dolphin (Tursiops truncatus), Pygmy Killer Whale (Feresa attenuata), Atlantic Spotted Dolphin (Stenella plagiodon/frontalis), Melon-Headed Whale (Peponocephala electra), Humpback Whale (Megeptera novaeangliae), Spinner Dolphin (Stenella longirostris), Pan tropical Spotted Dolphin (Stenella attenuata), Common Minke Whale (Balaenoptera acutorostrata), Short-Finned Pilot Whale (Globicephala macrorhynchus), Bottlenose Dolphin (Tursiops truncatus), Bryde's Whale (Balaenoptera edeni), Blue Whale (Balaenoptera musculus) | 13, 16, 17, 90, 122, 201, 207, 262, 340 |
| \| **Short-Finned Pilot Whale  (*Globicephala macrorhynchus*)** \| \| --- \| | Dalatiidae | 25.0 | 29 | High | Brachycladiidae, Campulidae, Nasitrematidae, Tetrabothriidae, Phyllobothriidae, Anisakidae, Pseudaliidae | Bottlenose Dolphin (Tursiops truncatus), Fraser's Dolphin (Lagenodelphis hosei), Melon-Headed Whale (Peponocephala electra), Northern Right Whale Dolphin (Lissodelphis borealis), Pacific White-Sided Dolphin (Lagenorhynchus obliquidens), Risso's Dolphin (Grampus griseus), Gray whale (Eschrichtius robustus), Longman's Beaked Whale (Mesoplodon pacificus), Pan tropical Spotted Dolphin (Stenella attenuata), False Killer Whale (Pseudorca crassidens), Rough-Toothed Dolphin (Steno bredanensis), Killer Whale (Orcinus orca), Sperm Whale (Physeter macrocephalus) | 13, 17, 85, 103, 116, 117, 118, 195, 207, 258, 343 |
| \| **Southern Right Whale Dolphin (*Lissodelphis peronii*)** \| \| --- \| | Somniosidae, Nototheniidae | 1.0 | 20 | High | Nasitrematidae, Pseudaliidae, Anisakidae, Tetrabothriidae, Opisthorchiidae, Phyllobothriidae | Dusky Dolphin (Lagenorhynchus obscurus), Bottlenose Dolphin (Tursiops truncatus), Long-Finned Pilot Whale (Globicephala melas), Fin Whale (Balaenoptera physalus), Hourglass Dolphin (Lagenorhynchus cruciger), Peale's Dolphin (Lagenorhynchus australis), Pacific White-Sided Dolphin (Lagenorhynchus obliquidens), Short Beaked Common Dolphin (Delphinus delphis) | 35, 36, 46, 181, 201, 202, 205, 319, 347 |
| \| **Spinner Dolphin  (*Stenella longirostris*)** \| \| --- \| | Dalatiidae, Lamnidae, Carcharhinidae, Tetraodontidae, Delphinidae | 22.0 | 27.5 | High | Echeneidae, Lepadidae, Anisakidae, Pseudaliidae, Spiruridae, Brachycladiidae, Nasitrematidae, Tetrabothriidae, Phyllobothriidae, Polymorphidae | Bottlenose Dolphin (Tursiops truncatus), Pan tropical Spotted Dolphin (Stenella attenuata), Rough-Toothed Dolphin (Steno bredanensis), Longman's Beaked Whale (Mesoplodon pacificus), Fraser's Dolphin (Lagenodelphis hosei), Melon-Headed Whale (Peponocephala electra), Fraser's Dolphin (Lagenodelphis hosei), Irrawaddy Dolphin (Orcaella brevirostris), Indo-pacific bottlenose dolphin(Tursiops aduncus), Short Beaked Common Dolphin (Delphinus delphis), Bryde's Whale (Balaenoptera edeni) | 13, 16, 17, 92, 120, 121, 149, 207, 212, 213, 317, 319, 323, 340, 341, 345 |
| Pan tropical Spotted Dolphin (*Stenella attenuata*) | Dalatiidae, Delphinidae, Caleocerdo cuvier | 25.3 | 28 | High | Phyllobothriidae, Tetrabothriidae, Brauninidae, Nasitrematidae, Anisakidae, Tetrameridae | Atlantic Spotted Dolphin (Stenella plagiodon/frontalis), Spinner Dolphin (Stenella longirostris), Clymene Dolphin (Stenella clymene), Bottlenose Dolphin (Tursiops truncatus), Fraser's Dolphin (Lagenodelphis hosei), Risso's Dolphin (Grampus griseus), Rough-Toothed Dolphin (Steno bredanensis), Melon-Headed Whale (Peponocephala electra), Short-Finned Pilot Whale (Globicephala macrorhynchus), Bryde's Whale (Balaenoptera edeni) | 13, 16, 17, 38, 39, 46, 103, 207, 209, 212, 217, 258, 319, 323, 340, 341, 345 |
| \| **Striped Dolphin  (*Stenella coeruleoalba*)** \| \| --- \| | Dalatiidae | 20.0 | 30 | High | Cyamidae , Pennellidae, Lepadidae, Coronulidae, Tetrabothriidae, Phyllobothriidae, Nasitrematidae, Brachycladiidae, Heterophyidae, Campulidae, Brachycladiidae, Anisakidae, Anguillicolidae, Tetrameridae, Pseudaliidae, Polymorphidae | Short Beaked Common Dolphin (Delphinus delphis), Fraser's Dolphin (Lagenodelphis hosei), Pacific White-Sided Dolphin (Lagenorhynchus obliquidens), Risso's Dolphin (Grampus griseus), Gray whale (Eschrichtius robustus), Fin Whale (Balaenoptera physalus) | 13, 16, 17, 89, 93, 150, 151, 205, 217, 258, 340, 341, 345 |
| \| **Guiana dolphin  (*Sotalia guianensis*)** \| \| --- \| | Dalatiidae, Delphinidae, Carcharhinidae | 15.0 | 31 | High | Nasitrematidae, Pseudaliidae, Anisakidae | Bottlenose Dolphin (Tursiops truncatus) | 17, 22, 46, 91, 201, 214, 252, 258, 340, 341 |
| \| **White-Beaked Dolphin  (*Lagenorhynchus albirostris*)** \| \| --- \| | Delphinidae | 8.1 | 17.2 | High | Anisakidae, Pseudaliidae, Cyamidae | Harbour Porpoise (Phocoena phocoena), Killer Whale (Orcinus orca), Long-Finned Pilot Whale (Globicephala melas), Atlantic White-Sided Dolphin (Lagenorhynchus acutus), Fin Whale (Balaenoptera physalus), Humpback Whale (Megeptera novaeangliae), Sei Whale (Balaenoptera borealis), Short Beaked Common Dolphin (Delphinus delphis), Bottlenose Dolphin (Tursiops truncatus) | 21, 23, 46, 50, 51, 55, 152, 194, 340, 344, 346 |
| \| **Beluga  (*Delphinapterus leucas*)** \| \| --- \| | Delphinidae, Ursidae, Somniosidae | 0.0 | 16 | Medium | Brachycladiidae, Campulidae, Diphyllobothriidae, Anisakidae, Tetrameridae, Pseudaliidae, Ascarididae, Polymorphidae | Narwhal (Monodon monoceros), Killer Whale (Orcinus orca) | 14, 17, 49, 99, 103, 193, 201, 218, 219, 221, 222, 319, 325, 340 |
| \| **Narwhal  (*Monodon monoceros*)** \| \| --- \| | Odobenidae, Delphinidae, Ursidae, Somniosidae, | -2.0 | 5 | High | Cyamidae, Anisakidae, Ascarididae, Pseudaliidae | Beluga (Delphinapterus leucas), Killer Whale (Orcinus orca) | 17, 46, 100, 145, 201, 221, 254, 253, 319, 320, 323, 340 |
| \| **Franciscana  (*Pontoporia blainvillei*)** \| \| --- \| | Delphinidae, Carcharhinidae, Hexanchidae | 16.0 | 27 | NA | Coronulidae, Cirolanidae, Cymothoidae, Cocconeidaceae, Anisakidae, Polymorphidae | NA | 17, 33, 98, 248, 249, 250, 251, 319, 323 |
| \| **Burmeister's Porpoise  (*Phocoena spinipinnis*)** \| \| --- \| | NA | 4.0 | 19.5 | Low | Campulidae, Heterophyidae, Nasitrematidae, Brauninidae, Anisakidae, Pseudaliidae, Polymorphidae, Cyamidae, Coronulidae | Bottlenose Dolphin (Tursiops truncatus), Dusky Dolphin (Lagenorhynchus obscurus), Commerson's Dolphin (Cephalorhynchus commersonii) | 17, 46, 86, 114, 284, 289 |
| Cochito [Vaquita]  (*Phocoena sinus*) | Lamnidae, Carcharhinidae, Alopiidae, Hexanchidae, | 17.0 | 32 | Low | Tetrameridae, Campulidae, Coronulidae | NA | 17, 46, 113, 175, 244, 245, 246, 289 |
| \| **Dall's Porpoise  (*Phocoenoides dalli*)** \| \| --- \| | Delphinidae, Lamnidae | 12.4 | 24 | NA | Pseudaliidae, Nasitrematidae, Brachycladiidae, Anisakidae, Tetrameridae, Phyllobothriidae, Polymorphidae, Cyamidae | Harbour Porpoise (Phocoena phocoena), Killer Whale (Orcinus orca), Fin Whale (Balaenoptera physalus), Blue Whale (Balaenoptera musculus), Gray whale (Eschrichtius robustus), Humpback Whale (Megeptera novaeangliae), Northern Right Whale Dolphin (Lissodelphis borealis), Pacific White-Sided Dolphin (Lagenorhynchus obliquidens), Risso's Dolphin (Grampus griseus), Sei Whale (Balaenoptera borealis) | 17, 46, 87, 88, 210, 232, 235, 236, 319 |
| \| **Finless Porpoise  (*Neophocaena phocaenoides*)** \| \| --- \| | Dalatiidae, Lamnidae | 5.7 | 25.6 | NA | Brachycladiidae, Nasitrematidae, Tetrameridae, Pseudaliidae, Diphyllobothriidae | Irrawaddy Dolphin (Orcaella brevirostris) | 17, 28, 29, 30, 87, 240, 241, 242, 258 |
| \| **Harbour Porpoise (*Phocoena phocoena*)** \| \| --- \| | Dalatiidae, Delphinidae, Lamnidae, Somniosidae, | 6.0 | 17 | Low | Brachycladiidae, Campulidae, Opisthorchiidae, Heterophyidae, Diphyllobothriidae, Anisakidae, Pseudaliidae, Ascarididae, Polymorphidae | Dall's Porpoise (Phocoenoides dalli), Bottlenose Dolphin (Tursiops truncatus), Killer Whale (Orcinus orca), Pacific White-Sided Dolphin (Lagenorhynchus obliquidens), White-Beaked Dolphin (Lagenorhynchus albirostris), Fin Whale (Balaenoptera physalus), Common Minke Whale (Balaenoptera acutorostrata), Humpback Whale (Megeptera novaeangliae) | 17, 21, 75, 76, 77, 103, 176, 177, 224, 227, 232, 233, 234, 258, 289, 319 |
| \| **Spectacled Porpoise (*Phocoena dioptrica*)** \| \| --- \| | NA | 5.5 | 9.5 | Low | NA | NA | 17, 32, 238, 289 |
| \| **Gray whale (*Eschrichtius robustus*)** \| \| --- \| | Delphinidae | 0.0 | 25 | Low | Notocotylidae, Tetrabothriidae, Pseudophyllidae, Polymorphidae | Killer Whale (Orcinus orca), Bottlenose Dolphin (Tursiops truncatus), Striped Dolphin (Stenella coeruleoalba), Short Beaked Common Dolphin (Delphinus delphis), Dall's Porpoise (Phocoenoides dalli), Short-Finned Pilot Whale (Globicephala macrorhynchus), Northern Right Whale Dolphin (Lissodelphis borealis), Pacific White-Sided Dolphin (Lagenorhynchus obliquidens), Risso's Dolphin (Grampus griseus) | 14, 19, 49, 103, 135, 222, 253, 340 |
| \| **Blue Whale  (*Balaenoptera musculus*)** \| \| --- \| | Dalatiidae, Delphinidae | 14.1 | 21.6 | Low | Notocotylidae, Tetrabothriidae, Anisakidae, Tetrameridae, Ascarididae, Polymorphidae | Killer Whale (Orcinus orca), Fin Whale (Balaenoptera physalus), Pacific White-Sided Dolphin (Lagenorhynchus obliquidens), Dall's Porpoise (Phocoenoides dalli), Bottlenose Dolphin (Tursiops truncatus), Rough-Toothed Dolphin (Steno bredanensis) | 16, 18, 49, 125, 141, 161, 164, 166, 258, 300, 304, 340 |
| \| **Bryde's Whale**  **(*Balaenoptera edeni*)** \| \| --- \| | Dalatiidae, Delphinidae | 18.5 | 19 | Low | Polymorphidae | Killer Whale (Orcinus orca), Long Beaked common Dolphin (Delphinus capensis), Rough-Toothed Dolphin (Steno bredanensis), Indo-pacific bottlenose dolphin (Tursiops aduncus), Spinner Dolphin (Stenella longirostris), Pan tropical Spotted Dolphin (Stenella attenuata) | 16, 18, 49, 128, 141, 164, 166, 174, 196, 246, 247, 258, 291, 292, 302, 340 |
| \| **Fin Whale**  **(*Balaenoptera physalus*)** \| \| --- \| | Dalatiidae, Delphinidae | 16.0 | 23 | Low | Brachycladiidae, Notocotylidae, Diphyllobothriidae, Phyllobothriidae, Tetrabothriidae, Anisakidae, Tetrameridae, Polymorphidae | Killer Whale (Orcinus orca), Blue Whale (Balaenoptera musculus), Atlantic White-Sided Dolphin (Lagenorhynchus acutus), Hourglass Dolphin (Lagenorhynchus cruciger), Northern Right Whale Dolphin (Lissodelphis borealis), Pacific White-Sided Dolphin (Lagenorhynchus obliquidens), Peale's Dolphin (Lagenorhynchus australis), Risso's Dolphin (Grampus griseus), Southern Right Whale Dolphin (Lissodelphis peronii), White-Beaked Dolphin (Lagenorhynchus albirostris), Dall's Porpoise (Phocoenoides dalli), Harbour Porpoise (Phocoena phocoena), Striped Dolphin (Stenella coeruleoalba) | 18, 21, 49, 103, 141, 164, 166, 167, 246, 258, 300, 304, 340 |
| Antarctic Minke Whale (*Balaenoptera bonaerensis*) | Dalatiidae, Delphinidae | -1.9 | 21.8 | Low | Pennellidae, Cyamidae, Anisakidae | Hourglass Dolphin (Lagenorhynchus cruciger), Peale's Dolphin (Lagenorhynchus australis), Pygmy Right Whale (Caperea marginata) | 46, 78, 140, 141, 142, 164, 166, 189, 204, 205, 206, 258, 282, 301, 340 |
| Common Minke Whale (*Balaenoptera acutorostrata*) | Dalatiidae, Delphinidae | 6.1 | 20.1 | Low | Pennellidae, Brachycladiidae, Diphyllobothriidae, Tetrabothriidae, Anisakidae, Cyamidae | Humpback Whale (Megeptera novaeangliae), Rough-Toothed Dolphin (Steno bredanensis), Bottlenose Dolphin (Tursiops truncatus), Hourglass Dolphin (Lagenorhynchus cruciger), Harbour Porpoise (Phocoena phocoena) | 21, 46, 140, 141, 144, 164, 166, 258, 300, 340 |
| \| **Sei Whale**  **(*Balaenoptera borealis*)** \| \| --- \| | Dalatiidae, Delphinidae | 5.0 | 18.8 | Low | Notocotylidae, Diphyllobothriidae, Tetrabothriidae, Polymorphidae, Tetrameridae, Anisakidae | Killer Whale (Orcinus orca), Hourglass Dolphin (Lagenorhynchus cruciger), Northern Right Whale Dolphin (Lissodelphis borealis), Pacific White-Sided Dolphin (Lagenorhynchus obliquidens), Peale's Dolphin (Lagenorhynchus australis), White-Beaked Dolphin (Lagenorhynchus albirostris), Pygmy Right Whale (Caperea marginata), North Atlantic Right whale (Eubalaena glacialis), Humpback Whale (Megeptera novaeangliae), Dall's Porpoise (Phocoenoides dalli) | 18, 49, 103, 140, 141, 164, 166, 206, 258, 282, 283, 301, 304, 340 |
| \| **Humpback Whale**  **(*Megeptera novaeangliae*)** \| \| --- \| | Dalatiidae, Delphinidae | 13.0 | 19 | Low | Diphyllobothriidae, Anisakidae, Brachycladiidae, Tetrameridae, Polymorphidae | Bottlenose Dolphin (Tursiops truncatus), Killer Whale (Orcinus orca), Common Minke Whale (Balaenoptera acutorostrata), Rough-Toothed Dolphin (Steno bredanensis), Dall's Porpoise (Phocoenoides dalli), Atlantic White-Sided Dolphin (Lagenorhynchus acutus), Long-Finned Pilot Whale (Globicephala melas), Melon-Headed Whale (Peponocephala electra), Northern Right Whale Dolphin (Lissodelphis borealis), Pacific White-Sided Dolphin (Lagenorhynchus obliquidens), White-Beaked Dolphin (Lagenorhynchus albirostris), Harbour Porpoise (Phocoena phocoena), North Atlantic Right whale (Eubalaena glacialis), Sei Whale (Balaenoptera borealis), North Pacific Right Whale (Eubalaena japonica) | 17, 18, 21, 49, 103, 138, 162, 202, 204, 205, 258, 261, 300, 340 |
| \| **Bowhead Whale**  **(*Balaena mysticetus*)** \| \| --- \| | Delphinidae | -1.6 | 2 | Low | Brachycladiidae, Notocotylidae, Phyllobothriidae, Polymorphidae, Tetrameridae | NA | 14, 18, 46, 49, 103, 163, 222, 280, 281, 340 |
| North Pacific Right Whale (*Eubalaena japonica*) | NA | 3.0 | 17 | Medium | Cyamidae, Tetrabothriidae, Polymorphidae | Humpback Whale (Megeptera novaeangliae) | 18, 46, 49, 104, 146, 147, 278, 279, 340 |
| North Atlantic Right Whale (*Eubalaena glacialis*) | NA | 2.2 | 21.8 | Medium | Cyamidae | Humpback Whale (Megeptera novaeangliae), Sei Whale (Balaenoptera borealis) | 18, 46, 49, 105, 146, 192, 198, 340 |
| \| **Southern Right Whale  (*Eubalaena australis*)** \| \| --- \| | Delphinidae | 13.0 | 19 | Medium | Cyamidae, Hydrophilidae, Tetrabothriidae, Polymorphidae | Killer Whale (Orcinus orca), Peale's Dolphin (Lagenorhynchus australis), Indo-Pacific Humpbacked Dolphin (Sousa chinensis) | 46, 49, 104, 106, 107, 146, 147, 190, 191, 202, 304, 340 |
| \| **Pygmy Right Whale  (*Caperea marginata*)** \| \| --- \| | Dalatiidae | 5.0 | 20 | Low | NA | Long-Finned Pilot Whale (Globicephala melas), Sei Whale (Balaenoptera borealis), Antarctic Minke Whale (Balaenoptera bonaerensis) | 46, 72, 73, 258, 340 |
| Indo-pacific bottlenose dolphin (*Tursiops aduncus*) | Dalatiidae | 13.4 | 24.1 | High | Anisakidae, Cyamidae, Pseudaliidae, Tetrameridae, Phyllobothriidae, Coronulidae | False Killer Whale (Pseudorca crassidens), Bryde's Whale (Balaenoptera edeni), Spinner Dolphin (Stenella longirostris) | 17, 74, 153, 155, 180, 190, 258, 276, 277, 287, 340, 341 |
| Sperm Whale  (*Physeter macrocephalus*) | Delphinidae, Dalatiidae, Somniosidae | 12.0 | 30.3 | Medium | Brachycladiidae, Tentaculariidae, Diphyllobothriidae, Tetrabothriidae, Anisakiidae, Phyllobothriidae, Tetrameridae, Polymorphidae, Pennellidae, Coronulidae, Lepadidae, Cyamidae, Echeneidae | Pacific White-Sided Dolphin (Lagenorhynchus obliquidens), Northern Right Whale Dolphin (Lissodelphis borealis), Risso's Dolphin (Grampus griseus), Melon-Headed Whale (Peponocephala electra), Fraser's Dolphin (Lagenodelphis hosei), Short-Finned Pilot Whale (Globicephala macrorhynchus), Southern Right Whale Dolphin (Lissodelphis peronii) | 16, 81, 327, 332, 333, 334, 339 |
| Pygmy Sperm Whale  (*Kogia breviceps*) | Delphinidae, Lamnidae | 26.9 | 30.9 | Medium |  | NA | 289, 304, 319, 328, 330, 331 |
| Dwarf Sperm Whale  (*Kogia sima*) | Delphinidae, Lamnidae | 26.0 | 26.4 | Medium | Phyllobothriidae, Tetraphyllidea incertae sedis, Anisakidae, Tetrameridae, Pseudaliidae, Polymorphidae, Pennellidae | NA | 16, 120, 289, 328, 329, 330, 331, 332, 335 |
| References for Species Associations (and sources cited within) | 19, 25, 32, 36, 37, 42, 43, 45, 46, 48, 50, 51, 52, 55, 58, 59, 65, 73, 80, 82, 86, 87, 90, 92, 104, 122, 132, 161, 162, 168, 169, 170, 186, 205, 208, 210, 211, 213, 215, 216, 220, 225, 226, 228, 229, 230, 231, 255, 256, 260, 263, 264, 267, 268, 269, 270, 272, 274, 286, 288, 291, 295, 296, 297, 298, 299, 304, 312, 327, 330 | | | | | | |

References for Table S1

1. Baker, A. N. 2001. Status, relationships, and distribution of *Mesoplodon bowdoini* Andrews, 1908 (Cetacea: Ziphidae). *Mar. Mamm. Sci.* **17**: 473–493.
2. Hobson, R. P., & Martin, A. R. 1996. Behaviour and dive times of Arnoux’s beaked whales, *Berardius arnuxii*, at narrow leads in fast ice. *Can. J. Zool.* **74**: 388–393.
3. Laporta, P., Praderi, R., Little, V., & Le Bas, A. 2005. An Andrew’s beaked whale *Mesoplodon bowdoini* (Cetacea, Ziphiidae) stranded on the Atlantic Coast of Uruguay. *LAJAM* **4**: 101–111.
4. Besharse, J. C. 1971. Maturity and sexual dimorphism in the skull, mandible, and teeth of the Beaked Whale, *Mesoplodon densirostris*. *J. Mammal.* **52**: 297–315.
5. Mead, J. G., Walker, W. A., & Houck, W. J. 1982. Biological observations on *Mesoplodon carlhubbsi* (Ceatacea: Ziphiidae). *Smithson. Contrib. Zool.* **344**: 1–25.
6. Mead, J. G., & Baker, A. N. 1987. Notes on the rares beaked whale, *Mesoplodon hectori*. *J. Roy. Soc. New Zeal.* **17**: 303–312.
7. Walker, W. A., Mead, J. G., & Brownell Jr., R. L. 2002. Diets of Baird’s Beaked Whales, *Berardius bairdii*, in the Southern Sea of Okhotsk and off the Pacific coast of Honshu, Japan. *Mar. Mamm. Sci.* **18**: 902–919.
8. Reeves, R. R., & Mitchell, E. 1993. Status of Baird’s Beaked Whales, *Berardius bairdii*. *Can. Field Nat.* **107**: 509–523.
9. Mead, J. G. 1984. Survey of reproductive data for the Beaked Whales (Ziphiidae). *Report of the International Whaling Commission*: 91–96.
10. Santos, M. B., Pierce, G. J., Herman, J., López, A., Guerra, A., Mente, E., & Clarke, M. R. 2001. Feeding ecology of Cuvier’s beaked whale (*Ziphius cavirostris*): a review with new information on the diet of this species. *J. Mar. Bio. Assoc.UK* **81**: 687–694.
11. McSweeney, D. J., Baird, R. W., & Mahaffy, S. D. 2007. Site fidelity, associations, and movements of Cuvier’s (*Ziphius cavirostris*) and Blainville's (*Mesoplodon densirostris*) Beaked Whales off the Island of Hawai'i. *Mar. Mamm. Sci.* **23**: 666–687.
12. Macleod, C. D., Perrin, W. F., Pitman, R., Barlow, J., Ballance, L., D’Amico, A., *et al.* 2006. Known and inferred distributions of beaked whale species (Cetacea: Ziphiidae). *J. Cetacean Res. Manage.* **7**: 271–286.
13. Davis, R. W., Fargion, G. S., May, N., Leming, T. D., Baumgartner, M., Evans, *et al*. 1998. Physical habitat of cetaceans along the continental slope in the north-central and western Gulf of Mexico. *Mar. Mamm. Sci.* **14**: 490–507.
14. Moore, S. E., Demaster, D. P., & Dayton, P. K. 2000. Cetacean habitat selection in the Alaskan arctic during summer and autumn. *Arctic* **53**: 432–447.
15. López, A., Pierce, G. J., Valeiras, X., Santos, M. B., & Guerra, A. 2004. Distribution patterns of small cetaceans in Galician waters. *J. Mar. Bio. Assoc.UK* **84**: 283–294.
16. Ballance, L. T., & Pitman, R. L. 1998. Cetaceans of the western tropical Indian Ocean: distribution, relative abundance, and comparisons with cetacean communities of two other tropical ecosystems. *Mar. Mamm. Sci.* **14**: 429–459.
17. Hoelzel, A. R. (Ed.). 2002. *Marine mammal biology: an evolutionary approach*. Malden, MA: Blackwell Publishing.
18. Jefferson, T. A., Webber, M. A., & Pitman, R. L. 2008. *Marine Mammals of the World: a comprehensive guide to their identification*. San Diego, CA: Elsevier.
19. Wolman, A. A. 1985. Gray Whale *Eschrichtius robustus* (Lilljeborg, 1861). In S. H. Ridgway & R. Harrison (Eds.), *Handbook of Marine Mammals: Volume 3 The sirenians and baleen whales*. Toronto, ON: Academic Press Inc. (pp. 67–90).
20. Mullin, K. D., & Fulling, G. L. 2004. Abundance of cetaceans in the oceanic northern Gulf of Mexico, 1996-2001. *Mar. Mamm. Sci.* **20**: 787–807.
21. Simard, P., Lawlor, J. L., & Gowans, S. 2006. Temporal variability of cetaceans near Halifax, Nova Scotia. *Can. Field Nat.* **120**: 93–99.
22. Azevedo, A. F., Viana, S. C., Oliveira, A. M., & Van Sluys, M. 2005. Group characteristics of marine tucuxis (*Sotalia fluviatilis*) (Cetacea: Delphinidae) in Guanabara Bay, south-eastern Brazil. *J. Mar. Biol. Assoc. UK* **85**: 209–212.
23. MacLeod, C. D., Weir, C. R., Santos, M. B., & Dunn, T. E. 2008. Temperature-based summer habitat partitioning between white-beaked and common dolphins around the United Kingdom and Republic of Ireland. *J. Mar. Biol. Assoc. UK* **88**: 1193 – 1198.
24. Cañadas, A., Sagarminaga, R., & García-Tiscar, S. 2003. Cetacean distribution related with depth and slope in the Mediterranean waters off southern Spain. *Deep-Sea Res. (1 Oceanogr. Res. Pap.)* **49**: 2053–2073.
25. Van Waerebeek, K., Barnett, L., Camara, A., Cham, A., Diallo, M., Djiba, *et al.* 2004. Distribution, status, and biology of the Atlantic Humpback Dolphin, *Sousa teuszii* (Kükenthal, 1892). *Aquat. Mamm.* **30**: 56–83.
26. Weinrich, M. T., Belt, C. R., & Morin, D. 2001. Behavior and ecology of the Atlantic White-sided dolphin (*Lagenorhynchus acutus*) in coastal New England waters. *Mar. Mamm. Sci.* **17**: 231–248.
27. Selzer, L. A., & Payne, P. M. 1988. The distribution of White-sided (*Lagenorhychus acutus*) and Common Dolphins (*Delphinus delphis*) vs. environmental features of the continental shelf of the Northeastern United States. *Mar. Mamm. Sci.* **4**: 141–153.
28. Jefferson, T. A., Robertson, K. M., & Wang, J. Y. 2002. Growth and reproduction of the finless porpoise in southern China. *Raff. Bull. Zool.* **10**: 105–113.
29. Barros, N. B., Jefferson, T. A., & Parsons, E. C. M. 2002. Food habits of Finless Porpoises (*Neophocaena phocaenoides*) in Hong Kong waters. *Raff. Bull. Zool.* **10**: 115–123.
30. Shirakihara, K., Shirakihara, M., & Yamamoto, Y. 2007. Distribution and abundance of finless porpoise in the Inland Sea of Japan. *Mar. Biol.* **150**: 1025–1032.
31. Best, P. B., & Abernethy, R. B. 1994. Heaviside’s Dolphin *Cephalorhynchus heavisidii* (Gray, 1828). In S. H. Ridgway & R. Harrison (Eds.), *Handbook of Marine Mammals: Volume 5 The first book of dolphins*. San Diego, CA: Academic Press Inc. (pp. 289–310).
32. Brownell Jr., R. L., & Clapham, P. J. 1999. Spectacled Porpoise *Phocoena dioptrica* Lahille, 1912. In S. H. Ridgway & R. Harrison (Eds.), *Handbook of Marine Mammals: Volume 6 The second book of dolphins and the porpoises*. Toronto, ON: Academic Press Inc. (pp. 379–391).
33. Crespo, E. A., & González, R. 1998. Group size and distributional range of the Franciscana, *Pontoporia blainvillei*. *Mar. Mamm. Sci.* **14**: 845–849.
34. Dalebout, M. L., Ross, G. J. B., Baker, C. S., Anderson, R. C., Best, P. B., Cockcroft, V. G., Hinsz, H. L., Peddemors, V., & Pitman, R. L. 2003. Appearance, distribution and genetic distinctiveness of Longman’s Beaked Whale, *Indopacetus pacificus*. *Mar. Mamm. Sci.* **19**: 421–461.
35. Newcomer, M. W., Jefferson, T. A., & Brownell Jr., R. L. 1996. *Lissodelphis peroni*. *Mamm. Species* **531**: 1–5.
36. Jefferson, T. A., Newcomer, M. W., Leatherwood, S., & Waerebeek, K. Van. 1994. Right Whale Dolphins *Lissodelphis borealis* (Peale, 1848) and *Lissodelphis peronii* (Lacépède, 1804). In S. H. Ridgway & R. Harrison (Eds.), *Handbook of Marine Mammals: Volume 5 The first book of dolphins*. Toronto, ON: Academic Press Inc. (pp. 335–362).
37. Jefferson, T. A., & Karczmarski, L. 2001*. Sousa chinensis*. *Mamm. Species*: 1–9.
38. Perrin, W. F., Mitchell, E. D., Mead, J. G., Caldwell, D. K., Caldwell, M. C., van Bree, P. J. H. *et al.* 1987. Revision of the Spotted Dolphins, *Stenella spp*. *Mar. Mamm. Sci.* **3**: 99–170.
39. Perrin, W. F., & Hohn, A. A. 1994. Pantropical Spotted Dolphin *Stenella attenuata*. In S. H. Ridgway & R. Harrison (Eds.), *Handbook of Marine Mammals: Volume 5 The first book of dolphins*. San Diego, CA: Academic Press Inc. (pp. 71–98).
40. Stacey, P. J., & Leatherwood, S. 1997. The Irrawaddy Dolphin, *Orcaella brevirostris*: A summary of current knowledge and recommendations for conservation action. *Asian Mar. Biol.* **14**: 195–214.
41. Baird, R. W. 2002. False Killer Whale, *Pseudorca crassidens*. In W. F. Perrin, B. Würsig, & J. G. M. Thewissen (Eds.), *Encyclopedia of Marine Mammals*. San Francisco, CA: Academic Press Inc. (pp. 405–406).
42. Stacey, P. J., Leatherwood, S., & Baird, R. W. 1994. *Pseudorca crassidens*. *Mamm. Species*: 1–6.
43. Pereira, J. N. D. S. G. 2008. Field notes on Risso’s Dolphin (*Grampus griseus*) distribution, social ecology, behaviour, and occurrence in the Azores. *Aquat. Mamm.* **34**: 426–435.
44. Chen, I., Watson, A., & Chou, L.-S. 2011. Insights from life history traits of Risso’s dolphins (*Grampus griseus*) in Taiwanese waters: Shorter body length characterizes northwest Pacific population. *Mar. Mamm. Sci.* **27**: 43–64.
45. Baird, R. W., & Stacey, P. J. 1991. Status of Risso’s Dolphin, *Grampus griseus*, in Canada. *Can. Field Nat.* **105**: 233–242.
46. Klinowska, M. 1991. *Dolphins, Porpoises and Whales of the World: The IUCN Red Data Book*. Gland, Switzerland and Cambridge, U.K.: IUCN.
47. Marsh, H., Lloze, R., Heinsohn, G. E., & Kasuya, T. 1989. Irrawaddy Dolphin *Orcaella brevirostris* (Gray, 1866). In S. H. Ridgway & R. Harrison (Eds.), *Handbook of Marine Mammals: Volume 4 River dolphins and the larger toothed whales*. San Diego, CA: Academic Press Inc. (pp. 101–118).
48. Ross, G. J. B., & Leatherwood, S. 1994. Pygmy Killer Whale *Feresa Attenuata* Gray 1874. In S. H. Ridgway & R. Harrison (Eds.), *Handbook of Marine Mammals: Volume 5 The first book of dolphins*. San Diego, CA: Academic Press Inc. (pp. 387–404).
49. Gaskin, D. E. 1982. *The Ecology of Whales and Dolphins*. Exeter, NH: Heinemann.
50. Reeves, R. R., Smeenk, C., Kinze, C. C., Brownell Jr., R. L., & Lien, J. 1999. White-beaked Dolphin *Lagenorhynchus albirostris* Gray, 1846. In S. H. Ridgway & R. Harrison (Eds.), *Handbook of Marine Mammals: Volume 6 The second book of dolphins and the porpoises*. Toronto, ON: Academic Press Inc. (pp. 1–30).
51. Reeves, R. R., Smeenk, C., Brownell Jr., R. L., & Kinze, C. C. 1999. Atlantic White-sided Dolphin. In S. H. Ridgway & R. Harrison (Eds.), *Handbook of Marine Mammals: Volume 6 The second book of dolphins and the porpoises*. Toronto, ON: Academic Press Inc. (pp. 31–56).
52. Brownell Jr., R. L., Walker, W. A., & Forney, K. A. 1999. Pacific White-sided Dolphin *Lagenorhynchus obliquidens* Gill, 1865. In S. H. Ridgway & R. Harrison (Eds.), *Handbook of Marine Mammals: Volume 6 The second book of dolphins and the porpoises*. Toronto, ON: Academic Press Inc. (pp. 57–84).
53. Dahlheim, M. E., & Towell, R. G. 1994. Occurrence and distribution of Pacific White-sided Dolphins (*Lagenorhynchus Obliquidens*) in southeastern Alaska, with notes on an attack by Killer Whales (*Orcinus Orca*). *Mar. Mamm. Sci.* **10**: 458–464.
54. Morton, A. B. 2000. Occurence, photo-identification and prey of Pacific White-sided Dolphins (*Lagenorhynchus obliquidens*) in the Broughton Archipelago, Canada 1984-1998. *Mar. Mamm. Sci.* **16**: 80–93.
55. Brownell Jr., R. L., Crespo, E. A., & Donahue, M. A. 1999. Peale’s Dolphin *Lagenorhynchus australis* (Peale, 1848). In S. H. Ridgway & R. Harrison (Eds.), *Handbook of Marine Mammals: Volume 6 The second book of dolphins and the porpoises*. Toronto, ON: Academic Press Inc. (pp. 105–120).
56. Viddi, F. A., & Lescrauwaet, A.-K. 2005. Insights on habitat selection and behavioural patterns of Peale’s Dolphins (*Lagenorhynchus australis*) in the Strait of Magellan, Southern Chile. *Aquat. Mamm.* **31**: 176–183.
57. Schiavini, A. C. M., Goodall, R. N. P., Lescrauwaet, A.-K., & Koen Alonso, M. 1997. Food habits of the Peale’s dolphin, *Lagenorhynchus australis*: review and new information. *Report of the International Whaling Commission* **0**: 827–834.
58. Brownell Jr., R. L., & Donahue, M. A. 1999. Hourglass Dolphin *Lagenorhynchus cruciger* (Quoy and Gaimard, 1824). In S. H. Ridgway & R. Harrison (Eds.), *Handbook of Marine Mammals: Volume 6 The second book of dolphins and the porpoises*. Toronto, ON: Academic Press Inc. (pp. 121–136).
59. Kruse, S., Caldwell, D. K., & Caldwell, M. C. 1999. Risso’s Dolphin *Grampus griseus* (G. Cuvier, 1812). In S. H. Ridgway & R. Harrison (Eds.), *Handbook of Marine Mammals: Volume 6 The second book of dolphins and the porpoises*. Toronto, ON: Academic Press Inc. (pp. 183–212).
60. Ribeiro, S., Viddi, F. A., Cordeiro, J. L., & Freitas, T. R. O. 2007. Fine-scale habitat selection of Chilean dolphins (*Cephalorhynchus eutropia*): interactions with aquaculture activities in southern Chiloé Island, Chile. *J. Mar. Biol. Assoc. UK* **87**: 119–128.
61. Jefferson, T. A., Odell, D. K., & Prunier, K. T. 1995. Notes on the biology of the Clymene dolphin (*Stenella clymene*) in the Northern Gulf of Mexico. *Mar. Mamm. Sci.* **11**: 564–573.
62. Fertl, D., Jefferson, T. A., Moreno, I. B., Zerbini, A. N., & Mullin, K. D. 2003. Distribution of the Clymene dolphin *Stenella clymene*. *Mamm. Rev.* **33**: 253–271.
63. Bryden, M. M., Harrison, R. J., & Lear, R. J. 1977. Some aspects of the biology of *Peponocephala electra* (Cetacea: Delphinidae) I. General and reproductive biology. *Aust. J. Mar. Fresh. Res.* **28**: 703–715.
64. Cannon, L. R. G. 1977. Some aspects of the biology of *Peponocephala electra* (Cetacea: Delphinidae) II.* Parasites. *Aust. J. Mar. Fresh. Res.* **28**: 717–722.
65. Watkins, W. A., Daher, M. A., Samuels, A., & Gannon, D. P. 1997. Observations of *Peponocephala electra*, the Melon-headed Whale, in the Southeastern Caribbean. *Caribbean J. Sci.* **33**: 34–40.
66. Brownell Jr., R. L., Ralls, K., Baumann-Pickering, S., & Poole, M. M. 2009. Behavior of melon-headed whales, *Peponocephala electra*, near oceanic islands. *Mar. Mamm. Sci.* **25**: 639–658.
67. Sekiguchi, K., Klages, N. T. W., & Best, P. B. 1996. The diet of strap-toothed whales (*Mesoplodon layardii*). *J. Zool., Lond.* **239**: 453–463.
68. Baker, A. N., & van Helden, A. L. 1999. New records of beaked whales, genus *Mesoplodon*, from New Zealand (Cetacea: Ziphiidae). *J. Roy. Soc. New Zeal.* **29**: 235–244.
69. Mead, J. G. 1989. Beaked Whales of the genus *Mesoplodon*. In S. H. Ridgway & R. Harrison (Eds.), *Handbook of Marine Mammals: Volume 4 River dolphins and the larger toothed whales*. San Diego, CA: Academic Press Inc. (pp. 349–430).
70. Slip, D. J., Moore, G. J., & Green, K. 1995. Stomach contents of a Southern Bottlenose Whale, *Hyperoodon planifrons*, stranded at Heard Island. *Mar. Mamm. Sci.* **11**: 575–584.
71. Macleod, C. D., Santos, M. B., & Pierce, G. J. 2003. Review of data on diets of beaked whales: evidence of niche separation and geographic segregation. *J. Mar. Biol. Assoc. UK* **83**: 651–665.
72. Sekiguchi, K., & Kaczmaruk, B. Z. 1992. On the feeding habits and baleen morphology of the Pygmy Right Whale *Caperea marginata*. *Mar. Mamm. Sci.* **8**: 288–293.
73. Baker, A. N. 1985. Pygmy Right Whale *Caperea marginata* (Gray, 1846). In S. H. Ridgway & R. Harrison (Eds.), *Handbook of Marine Mammals: Volume 3 The sirenians and baleen whales*. Toronto, ON: Academic Press Inc. (pp. 345–354).
74. Möller, L. M., Allen, S. J., & Harcourt, R. G. 2002. Group characteristics, site fidelity and seasonal abundance of Bottlenose Dolphins *Tursiops aduncus* in Jervis Bay and Port Stephens, South-Eastern Australia. *Aust. Mammal.* **24**: 11–21.
75. Börjesson, P., Berggren, P., & Ganning, B. 2003. Diet of Harbor Porpoises in the Kattegat and Skagerrak Seas: accounting for individual variation and sample size. *Mar. Mamm. Sci.* **19**: 38–58.
76. Gannon, D. P., Craddock, J. E., & Read, A. J. 1998. Autumn food habits of Harbor Porpoises, *Phocoena phocoena*, in the Gulf of Maine. *Fish. Bull.* **96**: 428–437.
77. Raum-Suryan, K. L., & Harvey, J. T. 1998. Distribution and abundance of habitat use by Harbour Porpoise, *Phocoena phocoena*, off the northern San Juan Islands, Washington. *Fish. Sci.* **96**: 808–822.
78. Tamura, T., & Konishi, K. 2009. Feeding habits and prey consumption of Antarctic Minke Whale (*Balaenoptera bonaerensis*) in the southern ocean. *J. Northwest Atl. Fish. Sci.* **42**: 13–25.
79. Heyning, J. E. 1989. Cuvier’s Beaked Whale *Ziphius cavirostris* G. Cuvier, 1823. In S. H. Ridgway & R. Harrison (Eds.), *Handbook of Marine Mammals: Volume 4 River dolphins and the larger toothed whales*. Toronto, ON: Academic Press Inc. (pp. 289–308).
80. Anderson, R. C., Clark, R., Madsen, P. T., Johnson, C., Kiszka, J., & Breysse, O. 2006. Observations of Longman’s Beaked Whale (*Indopacetus pacificus*) in the Western Indian Ocean. *Aquat. Mamm.* **32**: 223–231.
81. Gaskin, D. E. 1972. *Whales, Dolphins and Seals*. Toronto, ON: The MacMillan Company of Canada Ltd.
82. Brownell Jr., R. L., & Cipriano, F. 1999. Dusky Dolphin *Lagenorhynchus obscurus* (Gray, 1828). In S. H. Ridgway & R. Harrison (Eds.), *Handbook of Marine Mammals: Volume 6 The second book of dolphins and the porpoises*. Toronto, ON: Academic Press Inc. (pp. 85–104).
83. Blanco, C., Raduán, M. Á., & Raga, J. A. 2006. Diet of Risso’s dolphin (*Grampus griseus*) in the western Mediterranean Sea. *Sci. Mar.* **70**: 407–411.
84. Odell, D. K., & McClune, K. M. 1999. False Killer Whale *Pseudorca crassidens* (Owen, 1846). In S. H. Ridgway & R. Harrison (Eds.), *Handbook of Marine Mammals: Volume 6 The second book of dolphins and the porpoises*. Toronto, ON: Academic Press Inc. (pp. 213–243).
85. Bernard, H. J., & Reilly, S. B. 1999. Pilot Whales *Globicephala* Lesson, 1828. In S. H. Ridgway & R. Harrison (Eds.), *Handbook of Marine Mammals: Volume 6 The second book of dolphins and the porpoises*. Toronto, ON: Academic Press Inc. (pp. 245–279).
86. Brownell Jr., R. L., & Clapham, P. J. 1999. Burmeister’s Porpoise *Phocoena spinipinniis* Burmeister, 1865. In S. H. Ridgway & R. Harrison (Eds.), *Handbook of Marine Mammals: Volume 6 The second book of dolphins and the porpoises*. Toronto, ON: Academic Press Inc. (pp. 393–410).
87. Kasuya, T. 1999. Finless Porpoise *Neophocaena phocaenoides* (G. Cuvier, 1829). In S. H. Ridgway & R. Harrison (Eds.), *Handbook of Marine Mammals: Volume 6 The second book of dolphins and the porpoises*. Toronto, ON: Academic Press Inc. (pp. 411–442).
88. Jefferson, T. A. 1991. Observations on the distribution and behaviour of Dall’s porpoise (*Phocoenoides dalli*) in Monterey Bay, California. *Aquat. Mamm.* **17**: 12–19.
89. Perrin, W. F., Robertson, K. M., & Walker, W. A. 2008. Diet of the Striped Dolphin, Stenella coeruleoalba, in the Eastern tropical Pacific Ocean. *NOAA Technical Memorandum* NOAA-TM-NMFS-SWFSA-418
90. Miyazaki, N., & Perrin, W. F. 1994. Rough-toothed dolphin *Steno bradanensis* (Lesson, 1828). In S. H. Ridgway & R. Harrison (Eds.), *Handbook of Marine Mammals: Volume 5 The first book of dolphins*. San Diego, CA: Academic Press Inc. (pp. 1–21).
91. Silva, V. M. F. da, & Best, R. C. 1994. Tucuxi *Sotalia fluviatilis* (Gervais, 1853). In S. H. Ridgway & R. Harrison (Eds.), *Handbook of Marine Mammals: Volume 5 The first book of dolphins*. San Diego, CA: Academic Press Inc. (pp. 43–69).
92. Perrin, W. F., & Gilpatrick Jr., J. W. 1994. Spinner Dolphin *Stenella longirostris* (Gray, 1828). In S. H. Ridgway & R. Harrison (Eds.), *Handbook of Marine Mammals: Volume 5 The first book of dolphins*. San Diego, CA (pp. 99–128).
93. Perrin, W. F., Wilson, C. E., & Archer II, F. I. 1994. Striped Dolphin *Stenella coeruleoalba* (Meyen, 1833). In S. H. Ridgway & R. Harrison (Eds.), *Handbook of Marine Mammals: Volume 5 The first book of dolphins*. San Diego, CA: Academic Press Inc. (pp. 129–159).
94. Perrin, W. F., Caldwell, D. K., & Caldwell, M. C. 1994. Atlantic Spotted Dolphin *Stenella frontalis* (G. Cuvier, 1829). In S. H. Ridgway & R. Harrison (Eds.), *Handbook of Marine Mammals: Volume 5 The first book of dolphins*. San Diego, CA: Academic Press Inc. (pp. 173–190).
95. Evans, W. E. 1994. Common Dolphin, White-bellied Porpoise *Delphinus delphis* Linnaeus, 1758. In S. H. Ridgway & R. Harrison (Eds.), *Handbook of Marine Mammals: Volume 5 The first book of dolphins*. San Diego, CA: Academic Press Inc. (pp. 191–224).
96. Perrin, W. F., Leatherwood, S., & Collet, A. 1994. Fraser’s Dolphin *Lagenodelphis hosei* Fraser, 1956. In S. H. Ridgway & R. Harrison (Eds.), *Handbook of Marine Mammals: Volume 5 The first book of dolphins*. San Diego, CA: Academic Press Inc. (pp. 225–240).
97. Slooten, E., & Dawson, S. M. 1994. Hector’s Dolphin, *Cephalorhynchus hectori*. In S. H. Ridgway & R. Harrison (Eds.), *Handbook of Marine Mammals: Volume 5 The first book of dolphins*. San Diego, CA: Academic Press Inc. (pp. 311–333).
98. Brownell Jr., R. L. 1989. Franciscana *Pontoporia blainvillei* (Gervais and d’Orbigny, 1844). In S. H. Ridgway & R. Harrison (Eds.), *Handbook of Marine Mammals: Volume 4 River dolphins and the larger toothed whales*. San Diego, CA: Academic Press Inc. (pp. 45–67).
99. Brodie, P. F. 1989. The white whale *Delphinapterus leucas* (Pallas, 1776). In S. H. Ridgway & R. Harrison (Eds.), *Handbook of Marine Mammals: Volume 4 River dolphins and the larger toothed whales*. San Diego, CA: Academic Press Inc. (pp. 119–144).
100. Hay, K. A., & Mansfield, A. W. 1989. Narwhal *Monodon monoceros* Linnaeus, 1758. In S. H. Ridgway & R. Harrison (Eds.), *Handbook of Marine Mammals: Volume 4 River dolphins and the larger toothed whales*. San Diego, CA: Academic Press Inc. (pp. 145–176).
101. Balcomb III, K. C. 1989. Baird’s Beaked Whale *Berardius bairdii* Stejneger, 1883: Arnoux's Beaked Whales *Berardius arnuxii* Duvernoy 1851. In S. H. Ridgway & R. Harrison (Eds.), *Handbook of Marine Mammals: Volume 4 River dolphins and the larger toothed whales*. San Diego, CA: Academic Press Inc. (pp. 261–288).
102. Mead, J. G. 1989. Shepherd’s Beaked Whale *Tasmacetus sheperdi* Oliver, 1937. In S. H. Ridgway & R. Harrison (Eds.), *Handbook of Marine Mammals: Volume 4 River dolphins and the larger toothed whales*. San Diego, CA: Academic Press Inc. (pp. 309–320).
103. Dailey, M. D., & Brownell Jr., R. L. 1972. A checklist of marine mammal parasites. In S. H. Ridgway (Ed.), *Mammals of the Sea*. Springfield, IL: Charles C. Thomas (pp. 528–589).
104. Scarff, J. E. 1986. Historic and present distribution of the right whale (*Eubalaena glacialis*) in the Eastern North Pacific south of 50^O^N and east of 180^O^W. *Rep. Int. Whal. Commn.*: 43–63.
105. Winn, H. E., Price, C. A., & Sorensen, P. W. 1986. The distributional biology of the Right Whale (*Eubalaena glacialis*) in the Western North Atlantic. *Rep. Int. Whal. Commn.*: 129–138.
106. Aguayo L., A., & Torres N., D. 1986. Records of the Southern Right Whale, *Eubalaena australis* (Desmoulins, 1822) from Chile between 1976 and 1982. *Rep. Int. Whal. Commn.*: 159–167.
107. Ohsumi, S., & Kasamatsu, F. 1986. Recent off-shore distribution of the Southern Right Whale in summer. *Rep. Int. Whal. Commn.*: 177–185.
108. Goodall, R. N. P., Galeazzi, A. R., Leatherwood, S., Miller, K. W., Cameron, I. S., Kastelein, R. K., & Sobral, A. P. 1988. Studies of Commerson’s Dolphins, *Cepahlorhynchus commersonii*, off Tiera del Fuego, 1979-1984, with a review of information on the species in the South Atlantic. *Rep. Int. Whal. Commn.*: 3–70.
109. Leatherwood, S., Kastelein, R. A., & Miller, K. W. 1988. Observations of Commerson’s Dolphin and other cetaceans in Southern Chile, January-February 1984. *Rep. Int. Whal. Commn.*: 71–83.
110. Bastida, R., Lichtschein, V., & Goodall, R. N. P. 1988. Food habits of *Cephalorhynchus commersonii* off Tiera del Fuego. *Rep. Int. Whal. Commn.*: 143–160.
111. Goodall, R. N. P., Norris, K. S., Galeazzi, A. R., Oporto, J. A., & Cameron, I. S. 1988. On the Chilean Dolphin, *Cephalorhynchus eutropia* (Gray, 1846). *Rep. Int. Whal. Commn.*: 197–257.
112. Dawson, S. M., & Slooten, E. 1988. Hector’s Dolphin, *Cephalorhynchus hectori*: distribution and abundance. *Rep. Int. Whal. Commn.*: 315–338.
113. Vidal, O. 1995. Population biology and incidental mortality of the Vaquita, *Phocoena sinus*. *Rep. Int. Whal. Commn.*: 247–273.
114. Goodall, R. N. P., Norris, K. S., Harris, G., Oporto, J. A., & Castello, H. P. 1995. Notes on the biology of the Burmeister’s Porpoise, *Phocoena spinipinnis*, off Southern South America. *Rep. Int. Whal. Commn.*: 317–347.
115. Desportes, G., & Mouritsen, R. 1993. Preliminary results on the diet of Long-finned Pilot Whales off the Faroe Islands. *Rep. Int. Whal. Commn.*: 305–324.
116. Kasuya, T., & Tai, S. 1993. Life history of Short-finned Piolet Whale stocks off Japan and a description of the fishery. *Rep. Int. Whal. Commn.*: 439–473.
117. Mintzer, V. J., Gannon, D. P., Barros, N. B., & Read, A. J. 2008. Stomach contents of mass-stranded Short-finned Pilot Whales (Globicephala macrorhynchus) from North Carolina. *Mar. Mamm. Sci.* **24**: 290–302.
118. Taylor, B. L., Baird, R., Dawson, S. M., Ford, J., Mead, J. G., Notarbartolo do Sciara, G., *et al.* 2011. Globicephala macrorhynchus. *IUCN 2011. IUCN Red List of Threatened Speces. Version 2011.2*.
119. Kleinenberg, S. E., Yablokov, A. V, Bel’kovich, B. M., & Tarasevich, M. N. 1969. *Investigation of the species Beluga* (P. O. Theodor, Ed.). Smithsonian Institute.
120. Fitch, J. E., & Brownell Jr., R. L. 1968. Fish otoliths in cetacean stomachs and their importance in interpreting feeding habits. *J. Fish. Res. Board Can.* **25**: 2561–2574.
121. Dolar, M. L. L., Walker, W. A., Kooyman, G. L., & Perrin, W. F. 2003. Comparative feeding ecology of Spinner Dolphins (*Stenella longirostris*) and Fraser’s Dolphins (*Lagenodelphis hosei*) in the Sulu Sea. *Mar. Mamm. Sci.* **19**: 1–19.
122. West, K. L., Mead, J. G., & White, W. 2011. *Steno bredanensis* (Cetacea: Delphinidae). *Mamm. Species* **43**: 177–189.
123. Mead, J. G. 2002. Shepherd’s Beaked Whale, *Tasmacetus shepherdi*. In W. F. Perrin, B. Würsig, & J. G. M. Thewissen (Eds.), *Encyclopedia of Marine Mammals*. San Francisco, CA: Academic Press Inc. (pp. 1078–1081).
124. Perrin, W. F. 2002. Common Dolphins, *Delphinus delphis, D. capensis*, and *D. tropicalis*. In W. F. Perrin, B. Würsig, & J. G. M. Thewissen (Eds.), *Encyclopedia of Marine Mammals*. San Francisco, CA: Academic Press Inc. (pp. 245–248).
125. Sears, R. 2002. Blue Whale, *Balaenoptera musculus*. In W. F. Perrin, B. Würsig, & J. G. M. Thewissen (Eds.), *Encyclopedia of Marine Mammals*. San Francisco, CA: Academic Press Inc. (pp. 112–116).
126. Gowans, S. 2002. Bottlenose Whales, *Hyperoodon ampullatus* and *H. planifrons*. In W. F. Perrin, B. Würsig, & J. G. M. Thewissen (Eds.), *Encyclopedia of Marine Mammals*. San Francisco, CA: Academic Press Inc. (pp. 128–129).
127. Rugh, D. J., & Shelden, K. E. W. 2002. Bowhead Whale, *Balaena mysticetus*. In W. F. Perrin, B. Würsig, & J. G. M. Thewissen (Eds.), *Encyclopedia of Marine Mammals*. San Francisco, CA: Academic Press Inc. (pp. 129–131).
128. Kato, H. 2002. Bryde’s Whales, *Balaenoptera edeni* and *B. brydei*. In W. F. Perrin, B. Würsig, & J. G. M. Thewissen (Eds.), *Encyclopedia of Marine Mammals*. San Francisco, CA: Academic Press Inc. (pp. 171–177).
129. Dawson, S. M. 2002. *Cephalorhynchus* Dolphins, *C. heavisidii, C. eutropia, C. hectori, and C. commersonii*. In W. F. Perrin, B. Würsig, & J. G. M. Thewissen (Eds.), *Encyclopedia of Marine Mammals*. San Francisco, CA: Academic Press Inc. (pp. 200–203).
130. Jefferson, T. A. 2002. Clymene Dolphin, *Stenella clymene*. In W. F. Perrin, B. Würsig, & J. G. M. Thewissen (Eds.), *Encyclopedia of Marine Mammals*. San Francisco, CA: Academic Press Inc. (pp. 234–236).
131. Heyning, J. E. 2002. Cuvier’s Beaked Whale, *Ziphius cavirostris*. In W. F. Perrin, B. Würsig, & J. G. M. Thewissen (Eds.), *Encyclopedia of Marine Mammals*. San Francisco, CA: Academic Press Inc. (pp. 305–307).
132. Jefferson, T. A., & Leatherwood, S. 1994. *Lagenodelphis hosei*. *Mamm. Species* **470**: 1–5.
133. Hammond, P. S., Bearzi, G., Bjøge, A., Forney, K., Karczmarski, L., Kasuya, T., *et al.* 2008. *Lagenodelphis hosei*. *IUCN 2011. IUCN Red List of Threatened Speces. Version 2011.2*.
134. Dolar, M. L. L. 2002. Fraser’s Dolphin, *Lagenodelphis hosei*. In W. F. Perrin, B. Würsig, & J. G. M. Thewissen (Eds.), *Encyclopedia of Marine Mammals*. San Francisco, CA: Academic Press Inc. (pp. 485–487).
135. Jones, M. Lou, & Swartz, S. L. 2002. Gray Whale, *Eschrichtius robustus*. In W. F. Perrin, B. Würsig, & J. G. M. Thewissen (Eds.), *Encyclopedia of Marine Mammals*. San Francisco, CA: Academic Press Inc. (pp. 524–536).
136. Goodall, R. N. P. 2002. Hourglass Dolphin, *Lagenorhynchus cruciger*. In W. F. Perrin, B. Würsig, & J. G. M. Thewissen (Eds.), *Encyclopedia of Marine Mammals*. San Francisco, CA: Academic Press Inc. (pp. 583–585).
137. Ross, G. J. B. 2002. Humpback Dolphins, *Sousa chinensis, S. plumbea*, and *S. teuszi*. In W. F. Perrin, B. Würsig, & J. G. M. Thewissen (Eds.), *Encyclopedia of Marine Mammals*. San Francisco, CA: Academic Press Inc. (pp. 585–589).
138. Clapham, P. J. 2002. Humpback Whale, *Megaptera novaeangliae*. In W. F. Perrin, B. Würsig, & J. G. M. Thewissen (Eds.), *Encyclopedia of Marine Mammals*. San Francisco, CA: Academic Press Inc. (pp. 589–592).
139. Arnold, P. W. 2002. Irrawaddy Dolphin, *Orcaella brevirostris*. In W. F. Perrin, B. Würsig, & J. G. M. Thewissen (Eds.), *Encyclopedia of Marine Mammals*. San Francisco, CA: Academic Press Inc. (pp. 652–654).
140. Perrin, W. F., & Brownell Jr., R. L. 2002. Minke Whales, *Balaenoptera acutorostrata* and *B. bonaerensis*. In W. F. Perrin, B. Würsig, & J. G. M. Thewissen (Eds.), *Encyclopedia of Marine Mammals*. San Francisco, CA: Academic Press Inc. (pp. 750–754).
141. Stewart, B. S., & Leatherwood, S. 1985. Minke Whale *Balaenoptera acutorostrata* Lacépède, 1804. In S. H. Ridgway & R. Harrison (Eds.), *Handbook of Marine Mammals: Volume 3 The sirenians and baleen whales*. Toronto, ON: Academic Press Inc. (pp. 91–136).
142. Dailey, M. D., & Vogelbein, W. K. 1991. Parasite fauna of three species of Antarctic whales with reference to their use as potential stock indicators. *Fish. Bull.* **89**: 355–365.
143. Gibson, D. I., Harris, E. A., Bray, R. A., Jepson, P. D., Kuiken, T., Baker, J. R., *et al.* 1998. A survey of the helminth parasites of cetaceans stranded on the coast of England and Wales during the period 1990 ± 1994. *J. Zool., Lond.* **244**: 563–574.
144. Akihiko, U., & Jun, A. 2000. The ectoparasites and endoparasites in the Minke Whale, *Balaenoptera acutorostrata* from the Western North Pacific Ocean. *J. Jpn. Vet. Med. Assoc.* **53**: 85–88.
145. Heide-Jørgensen, M. P. 2002. Narwhal, *Monodon monoceros*. In W. F. Perrin, B. Würsig, & J. G. M. Thewissen (Eds.), *Encyclopedia of Marine Mammals*. San Francisco, CA: Academic Press Inc. (pp. 783–787).
146. Cummings, W. C. 1985. Right Whales *Eubalaena glacialis* (Müller, 1776) and *Eubalaena australis* (Desmoulins, 1822). In S. H. Ridgway & R. Harrison (Eds.), *Handbook of Marine Mammals: Volume 3 The sirenians and baleen whales*. Toronto, ON: Academic Press Inc. (pp. 275–304).
147. Klumov, S. K. 1962. The Right Whales in the Pacific Ocean. *Trudy Inst. Okeanol.* **58**: 202–297.
148. Baird, R. W. 2002. Risso’s Dolphin, *Grampus griseus*. In W. F. Perrin, B. Würsig, & J. G. M. Thewissen (Eds.), *Encyclopedia of Marine Mammals*. San Francisco, CA: Academic Press Inc. (pp. 1037–1039).
149. Ralls, K., & Mesnick, S. L. 2002. Sexual Dimorphism. In W. F. Perrin, B. Würsig, & J. G. M. Thewissen (Eds.), *Encyclopedia of Marine Mammals*. San Francisco, CA: Academic Press Inc. (pp. 1071–1078).
150. Archer II, F. I. 2002. Striped Dolphin, *Stenella coeruleoalba*. In W. F. Perrin, B. Würsig, & J. G. M. Thewissen (Eds.), *Encyclopedia of Marine Mammals*. San Francisco, CA: Academic Press Inc. (pp. 1201–1203).
151. Hammond, P. S., Bearzi, G., Bjøge, A., Forney, K., Karczmarski, L., Kasuya, T., *et al.* 2008. *Stenella coeruleoalba*. *IUCN 2011. IUCN Red List of Threatened Speces. Version 2011.2*.
152. Kinze, C. C. 2002. White-beaked Dolphin, *Lagenorhynchus albirostris*. In W. F. Perrin, B. Würsig, & J. G. M. Thewissen (Eds.), *Encyclopedia of Marine Mammals*. San Francisco, CA: Academic Press Inc. (pp. 1332–1334).
153. Amir, O. A., Berggren, P., Ndaro, S. G. M., & Jiddawi, N. S. 2005. Feeding ecology of the Indo-Pacific bottlenose dolphin (*Tursiops aduncus*) incidentally caught in the gillnet fisheries off Zanzibar, Tanzania. *Estuar. Coast. Shelf Sci.* **63**: 429–437.
154. Hammond, P. S., Bearzi, G., Bjøge, A., Forney, K., Karczmarski, L., Kasuya, T., *et al.* 2008. *Tursiops truncatus.* *IUCN 2011. IUCN Red List of Threatened Speces. Version 2011.2*.
155. Hammond, P. S., Bearzi, G., Bjøge, A., Forney, K., Karczmarski, L., Kasuya, T., *et al.* 2008. *Tursiops aduncus.* *IUCN 2011. IUCN Red List of Threatened Speces. Version 2011.2*.
156. García-Godos, I., Van Waerebeek, K., Reyes, J. C., Alfaro-Shigueto, J., & Arias-Schreiber, M. 2007. Prey occurrence in the stomach contents of four small cetacean species in Peru. *LAJAM* **6**: 171–183.
157. Ballance, L. T. 1992. Habitat use patterns and ranges of the Bottlenose Dolphin in the Gulf of California, Mexico. *Mar. Mamm. Sci.* **8**: 262–274.
158. Blanco, C., Salomón, O., & Raga, J. A. 2001. Diet of the bottlenose dolphin (*T ursiops truncatus*) in the western Mediterranean Sea. *J. Mar. Biol. Assoc. UK* **81**: 1053–1058.
159. Barros, N. B., & Wells, R. S. 1998. Prey and feeding patterns of resident Bottlenose Dolphins (*Tursiops truncatus*) in Sarasota, Florida. *J. Mammal.* **79**: 1045–1059.
160. Barros, N. B., Parsons, E. C. M., & Jefferson, T. A. 2000. Prey of offshore bottlenose dolphins from the South China Sea. *Aquat. Mamm.* **26**: 2–6.
161. Yochem, P. K., & Leatherwood, S. 1985. Blue Whale *Balaenoptera musculus* (Linnaeus, 1758). In S. H. Ridgway & R. Harrison (Eds.), *Handbook of Marine Mammals: Volume 3 The sirenians and baleen whales*. Toronto, ON: Academic Press Inc. (pp. 193–240).
162. Winn, H. E., & Reichley, N. E. 1985. Humpback Whale *Megaptera novaeangliae* (Borowski, 1781). In S. H. Ridgway & R. Harrison (Eds.), *Handbook of Marine Mammals: Volume 3 The sirenians and baleen whales*. Toronto, ON: Academic Press Inc. (pp. 241–273).
163. Reeves, R. R., & Leatherwood, S. 1985. Bowhead Whale *Balaena mysticetus* Linnaeus, 1758. In S. H. Ridgway & R. Harrison (Eds.), *Handbook of Marine Mammals: Volume 3 The sirenians and baleen whales*. Toronto, ON: Academic Press Inc. (pp. 305–344).
164. Cummings, W. C. 1985. Bryde’s Whale *Balaenoptera edeni* Anderson, 1878. In S. H. Ridgway & R. Harrison (Eds.), *Handbook of Marine Mammals: Volume 3 The sirenians and baleen whales*. Toronto, ON: Academic Press Inc. (pp. 137–154).
165. Gambell, R. 1985. Sei Whale *Balanenoptera borealis* Lesson, 1828. In S. H. Ridgway & R. Harrison (Eds.), *Handbook of Marine Mammals: Volume 3 The sirenians and baleen whales*. Toronto, ON: Academic Press Inc. (pp. 155–170).
166. Gambell, R. 1985. Fin Whale *Balaenoptera physalus* (Linnaeus, 1758). In S. H. Ridgway & R. Harrison (Eds.), *Handbook of Marine Mammals: Volume 3 The sirenians and baleen whales*. Toronto, ON: Academic Press Inc. (pp. 171–192).
167. Aguilar, A. 2002. Fin Whale, *Balaenoptera physalus*. In W. F. Perrin, B. Würsig, & J. G. M. Thewissen (Eds.), *Encyclopedia of Marine Mammals*. San Francisco, CA: Academic Press Inc. (pp. 435–438).
168. Lipsky, J. D. 2002. Right Whale Dolphins *Lissodelphis borealis* and L. peronii. In W. F. Perrin, B. Würsig, & J. G. M. Thewissen (Eds.), *Encyclopedia of Marine Mammals*. San Francisco, CA: Academic Press Inc. (pp. 1030–1033).
169. Perrin, W. F., & Mead, J. G. 1994. Clymene Dolphin *Stenella clymene* (Gray, 1846). In S. H. Ridgway & R. Harrison (Eds.), *Handbook of Marine Mammals: Volume 5 The first book of dolphins*. Toronto, ON: Academic Press Inc. (pp. 161–171).
170. Goodall, R. N. P. 1994. Chilean Dolphin *Cephalorhynchus eutropia* (Gray 1846). In S. H. Ridgway & R. Harrison (Eds.), *Handbook of Marine Mammals: Volume 5 The first book of dolphins*. Toronto, ON: Academic Press Inc. (pp. 269–287).
171. Barros, N. B., Jefferson, T. A., & Parsons, E. C. M. 2004. Feeding habits of Indo-Pacific Humpback Dolphins (*Sousa chinensis*) stranded in Hong Kong. *Aquat. Mamm.* **30**: 179–188.
172. Jefferson, T. A. 1996. Morphology of the clymene dolphin (*Stenella clymene*) in the northern Gulf of Mexico. *Aquat. Mamm.* **22**: 35–43.
173. Mignucci-Giannoni, A. A., Hoberg, E. P., Siegel-Causey, D., & Williams, E. H. 1998. Metazoan parasites and other symbionts of cetaceans in the Caribbean. *J. Parasitol.* **84**: 939–946.
174. Santos, M. C. de O., Siciliano, S., Castro de Vincente, A. F., Alvarenga, F. S., Zampirolli, E., de Souza, S. P., *et al.* 2010. Cetacean records along São Paulo state coast, southeastern Brazil. *Braz. J. Oceano.* **58**: 123–142.
175. Hohn, A. A., Read, A. J., Fernández, S., Vidal, O., & Findley, L. T. 1996. Life history of the vaquita, *Phocoena sinus* (Phocoenidae, Cetacea). *J. Zool., Lond.* **239**: 235–251.
176. Read, A. J. 1990. Estimation of body condition in harbour porpoises, *Phocoena phocoena*. *Can. J. Zool.* **68**: 1962–1966.
177. Lockyer, C., Heide-Jørgensen, M. P., Jensen, J., Kinze, C. C., & Buus Sørensen, T. 2001. Age, length and reproductive parameters of harbour porpoises *Phocoena phocoena (L.)* from West Greenland. *ICES J. Mar. Sci.* **58**: 154–162.
178. Ferrero, R. C., & Walker, W. A. 1993. Growth and reproduction of the northern right whale dolphin, *Lissodelphis borealis*, in the offshore waters of the North Pacific Ocean. *Can. J. Zool.* **71**: 2335–2344.
179. Slooten, E. 1992. Age, growth, and reproduction in Hector’s dolphins. *Can. J. Zool.* **69**: 1689–1700.
180. Chantrapornsyl, S., Adulyanukosol, K., & Kittiwathanawong, K. 1996. Records of cetaceans in Thailand. *Phuket Mar. Biol. Center Res. Bull.* **61**: 39–63.
181. Rose, B., & Payne, A. I. L. 1991. Occurrence and behavior of the Southern Right whale Dolphin *Lissodelphis peronii* off Namibia. *Mar. Mamm. Sci.* **7**: 25–34.
182. Claver, J. A., Iniguez, M. A., Lombardo, D. M., & Lawzewitsch, I. von. 1992. Preliminary pbservations on the ovarian activity and sexual maturity in female Peale’s dolphin (*Lagenorhynchus australis*). *Aquat. Mamm.* **18**: 85–88.
183. Boy, C. C., Dellabianca, N., Goodall, R. N. P., & Schiavini, A. C. M. 2011. Age and growth in Peale’s dolphin (*Lagenorhynchus australis*) in subantarctic waters off southern South America. *Mamm. Biol.* **76**: 634–639.
184. Jefferson, T. A., Fertl, D., Bolaños-Jiménez, J., & Zerbini, A. N. 2009. Distribution of common dolphins (*Delphinus spp*.) in the western Atlantic Ocean: a critical re-examination. *Mar. Biol.* **156**: 1109–1124.
185. Berón-Vera, B., Pedraza, S. N., Raga, J. A., Gil de Pertierra, A., Crespo, E. A., Alonso, M. K., *et al.* 2001. Gastrointestinal helminths of Commerson’s dolphins *Cephalorhynchus commersonii* from central Patagonia and Tierra del Fuego. *Dis. Aquat. Org.* **47**: 201–208.
186. Wells, R. S., & Scott, M. D. 1999. Bottlenose Dolphin *Tursiops truncatus* (Montagu, 1821). In S. H. Ridgway & R. Harrison (Eds.), *Handbook of Marine Mammals: Volume 6 The second book of dolphins and the porpoises*. Toronto, ON: Academic Press Inc. (pp. 137–182).
187. Perrin, W. F., & Reilly, S. B. 1982. Reproductive parameters of dolphins and small whales of the family Deiphinidae. *Rep. Int. Whal. Commn.* **Special Is**: 97–133.
188. Torres, P., Oporto, J. A., Brieva, L. M., & Escare, L. 1992. Gastrointestinal Helminths of the cetaceans *Phocoena spinipinnis* (Burneister, 1865) and *Cephalorhynchus eutropis* (Gray, 1846) from the Southern coast of Chile. *J. Wildl. Dis.* **28**: 313–315.
189. Williams, R., Hedley, S. L., & Hammond, P. S. 2006. Modeling distribution and abundance of Antarctic baleen whales using ships of opportunity. *Ecol. Soc.* **11**: 1
190. Tormosov, D. D., Mikhaliev, Y. A., Best, P. B., Zemsky, V. A., Sekiguchi, K., & Brownell Jr., R. L. 1998. Soviet catches of southern right whales *Eubalaena australis*, 1951 ± 1971. Biological data and conservation implications. *Biol. Conserv*. **86**: 185–197.
191. Braham, H. W., & Rice, D. W. 1984. The Right whale, *Balaena glaacialis*. *Mari. Fish. Rev.* **46**: 38–44.
192. Mead, J. G. 1986. Twentieth-century records of Right Whales (*Eubalaena glacialis*) in the Northwestern Atlantic Ocean. *Rep. Int. Whal. Commn.* **Special Is**: 109–119.
193. Cosens, S. E., & Dueck, L. P. 1991. Group size and activity patterns of belugas (*Dephinapterus leucas*) and narwhals (*Monodon monoceros*) during spring migration in Lancaster Sound. *Can. J. Zool.* **69**: 1630–1635.
194. Canning, S. J., Santos, M. B., Reid, R. J., Evans, P. G. H., Sabin, R. C., Bailey, N., *et al.* 2008. Seasonal distribution of white-beaked dolphins (*Lagenorhynchus albirostris*) in UK waters with new information on diet and habitat use. *J. Mar. Biol. Assoc. UK* **88**: 1159–1166.
195. Kanaji, Y., Okamura, H., & Miyashita, T. 2011. Long-term abundance trends of the northern form of the short-finned pilot whale (*Globicephala macrorhynchus*) along the Pacific coast of Japan. *Mar. Mamm. Sci.* **27**: 477–492.
196. Pinto, R. M., Muniz-Pereira, L. C., Alves, V. C., & Siciliano, S. 2004. First report of a Helminth infection for Bryde’s Whale *Balaenoptera edeni* Andreson, 1878 (Cetacea, Balaenopteridae). *LAJAM*  **3**: 167–170.
197. Ferguson, M. C., Barlow, J., Fiedler, P., Reilly, S. B., & Gerrodette, T. 2006. Spatial models of delphinid (family Delphinidae) encounter rate and group size in the eastern tropical Pacific Ocean. *Ecol. Model.* **193**: 645–662.
198. Ridgway, S. H. 1996. Final report from the Right Whale necropsy team: results, analysis, and recommendations. *Naval Command and Ocean Surveillance Center. Tech. Doc. 2935* San Diego, CA.
199. Reeves, R. R., & Brownell, R. L. 2008. Report of the assessment workshop on Indo-Pacific Bottlenose Dolphins (*Tursiops aduncus*) with Solomon Islands as a case study. Secretariat of the Pacific Regional Environment Programme (SPREP) Training and Education Centre. Apia, Spain.
200. Culik, B. M. 2004. Review of small cetaceans; distribution, behaviour, migration and threats. *Marine Mammal Action Plan/Regional Seas Reports and Studies no. 177.* Bonn, Germany.
201. Beneditto, A. P. M. Di, & Siciliano, S. 2007. Stomach contents of the marine tucuxi dolphin (*Sotalia guianensis*) from Rio de Janeiro, south-eastern Brazil. *J. Mar. Biol. Assoc. UK* **87**: 253–254.
202. Best, P. B., Glass, J. P., Ryan, P. G., & Dalebout, M. L. 2009. Cetacean records from Tristan da Cunha, South Atlantic. *J. Mar. Biol. Assoc. UK* **89**: 1023–1032.
203. A, D., & DeBuffrenil, V. 1989. Acoustic signals of the Commerson’s Dolphin, *Cephalorhynchus commersonii*, in the Kerguelen Islands. *J. Mammal.* **70**: 449–452.
204. Lauriano, G., Fortuna, C. M., & Vacchi, M. 2010. Occurrence of killer whales (*Orcinus orca*) and other cetaceans in Terra Nova Bay, Ross Sea, Antarctica. *Antarct. Sci.* **23**: 139–143.
205. Miyazaki, N., & Hidehira, K. 1988. Sitting records of small cetaceans in the southern hemisphere. *Bull. Natn. Sci. Mus., Tokyo, Ser. A* **14**: 47–65.
206. Mizroch, S. A., & Rice, D. W. 2006. Have North Pacific killer whales switched prey species in response to depletion of the great whale populations? *Mar. Ecol. Prog. Ser.* **310**: 235–246.
207. Gannier, A. 2009. Comparison of odontocete populations of the Marquesas and Society Islands (French Polynesia). *Journal of the Marine Biological Association of the United Kingdon* **89**: 931–941.
208. Herzing, D. L., Moewe, K., & Brunnick, B. J. 2003. Interspecies interactions between Atlantic spotted dolphins, Stenella frontalis and bottlenose dolphins, Tursiops truncatus, on Great Bahama Bank, Bahamas. *Aquat. Mamm*. **29**: 335–341.
209. Pitman, R. L., O’Sullivan, S., & Mase, B. 2003. Killer whales (Orcinus orca) attack a school of pantropical spotted dolphins (*Stenella attenuata*) in the Gulf of Mexico. *Aquat. Mamm.* **29**: 321–324.
210. Psarakos, S., Herzing, D. L., & Marten, K. 2003. Mixed-species associations between Pantropical Spotted dolphins (*Stenella attenuata*) and Hawaiian Spinner dolphins (*Stenella longirostris*) off Oahu, Hawaii. *Aquat. Mamm.* **29**: 390–395.
211. Herzing, D. L., & Johnson, C. M. 1997. Interspecific interactions between Atlantic Spotted dolphins (*Stenella frontalis*) and Bottlenose dolphins (*Tursiops truncatus*) in the Bahamas, 1985–1995. *Aquat. Mamm.* **23**: 85–99.
212. Moreno, I. B., Zerbini, A. N., Danilewicz, D., Santos, M. C. D. O., Simões-Lopes, P. C., Lailson-Brito Jr., J., *et al.* 2005. Distribution and habitat characteristics of dolphins of the genus *Stenella* (Cetacea: Delphinidae) in the southwest Atlantic Ocean. *Mar. Ecol. Prog. Ser.* **300**: 229–240.
213. Silva Jr, J. M., Lima Silva, F. J. De, Sazima, C., & Sazima, I. 2007. Trophic relationships of the spinner dolphin at Fernando de Noronha Archipelago, SW Atlantic. *Sci. Mar.* **71**: 505–511.
214. Flores, P. A., & Silva, V. M. Da. 2002. Tucuxi and Guiana Dolphin *Sotalia fluviatilis* and *S. guianensis*. In W. F. Perrin, B. Würsig, & J. G. M. Thewissen (Eds.), *Encyclopedia of Marine Mammals*. San Francisco, CA: Academic Press Inc. (pp. 1188–1192).
215. Wedekin, L. L., Daura-Jorge, F. G., & Simões-Lopes, P. C. 2004. An aggressive interaction between Bottlenose Dolphins (*Tursiops truncatus*) and estuarine Dolphins (*Sotalia guianensis*) in southern Brazil. *Aquat. Mamm.* **30**: 391–397.
216. Acevedo-Gutiérrez, A., DiBerardinis, A., Larkin, S., Larkin, K., & Forestell, P. 2005. Social interactions between Tucuxis and Bottlenose dolphins in Gandoca-Manzanillo, Costa Rica. *LAJAM* **4**: 49–54.
217. Jefferson, T. A., & Curry, B. E. 2003. *Stenella clymene*. *Mamm. Species* **726**: 1–5.
218. Frost, K. J., Russell, R. B., & Lowery, L. F. 1992. Killer Whales, *Orcinus orca*, in the southeastern Berring Sea: recent sightings and predation on ther marine mammals. *Mar. Mamm. Sci.* **8**: 110–119.
219. Thiemann, G. W., Iverson, S. J., & Stirling, I. 2008. Polar Bear diets and arctic marine food webs: insights from fatty acid analysis. *Ecol. Monogr.* **78**: 591–613.
220. Orr, J. R., & Harwood, L. A. 1998. Possible aggressive behavior between a Narwhal (*Monodon monoceros*) and a Beluga (*Delphinapterus leucas*). *Mar. Mamm. Sci.* **14**: 182–185.
221. Smith, T. G., & Sjare, B. 1990. Prdation of Belugas and Narwhals by Polar Bears in nearshore areas of the Canadian High Arctic. *Arctic* **43**: 99–102.
222. Melinikov, V. V, & Zagrebin, I. A. 2005. Killer whale predation in coastal waters of the Chukotka peninsula. *Mar. Mamm. Sci.* **21**: 550–556.
223. Shelden, K. E., Rugh, D. J., Mahoney, B. A., & Dahlheim, M. E. 2003. Killer Whale predation on Belugas in Cook Inlet, Alaska: implications for a depleted population. *Mar. Mamm. Sci.* **19**: 529–544.
224. Taguchi, M., Ishikawa, H., & Matsuishi, T. 2010. Seasonal distribution of Harbour Porpoise (*Phocoena phocoena*) in Japanese waters inferred from stranding and bycatch records. *Mamm. Study* **35**: 133–138.
225. Visser, I. N., Zaeschmar, J., Halliday, J., Abraham, A., Ball, P., Bradley, R., *et al.* 2010. First record of predation on False Killer Whales (*Pseudorca crassidens*) by Killer Whales (*Orcinus orca*). *Aquat. Mamm.* **36**: 195–204.
226. Read, A. J. 1999. Harbour Porpoise *Phocoena phocoena* (Linnaeus, 1758). In S. H. Ridgway & R. G. Harrison (Eds.), *Handbook of Marine Mammals: Volume 6 The second book of dolphins and the porpoises*. San Diego, CA: Academic Press Inc. (pp. 323–378).
227. Watts, P., & Gaskin, D. E. 1985. Habitat index analysis of the Harbor Porpoise (*Phocoena phocoena*) in the southern Bay of Fundy, Canada. *J. Mammal.* **66**: 733–744.
228. Baird, R. W. 1998. An interaction between Pacific White-Sided Dolphins and a neonatal harbor porpoise. *Mammalia* **62**: 129–134.
229. Willis, P. M., Crespi, B. J., Dill, L. M., Baird, R. W., & Hanson, M. B. 2004. Natural hybridization between Dall’s porpoises (*Phocoenoides dalli*) and harbour porpoises (*Phocoena phocoena*). *Can. J. Zool.* **82**: 828–834.
230. Ross, H. M., & Wilson, B. 1996. Violent interactions between bottlenose dolphins and harbour porpoises. *Proc. Roy. Soc. Lond. Lond. B.* **263**: 283–286.
231. Haelters, J., & Everaarts, E. 2011. Two cases of physical interaction between White-Beaked Dolphins (*Lagenorhynchus albirostris*) and juvenile Harbour Porpoises (*Phocoena phocoena*) in the southern North Sea. *Aquat. Mamm.* **37**: 198–201.
232. Dahlheim, M. E., & White, P. A. 2010. Ecological aspects of transient killer whales *Orcinus orca* as predators in southeastern Alaska. *Wildl. Biol.* **16**: 308–322.
233. Arnold, P. W. 1972. Predation on Harbour Porpoise, Phocoena phocoena, by a White Shark, Carcharodon carcharias. *J. Fish. Res. Board Can.* **29**: 1213–1214.
234. Williamson, G. 1963. Common Porpoise from the Stomach of a Greenland Shark. *J. Fish. Res. Board Can.* **20**: 1085–1087.
235. Jefferson, T. A. 1991. Observations on the distribution and behaviour of Dall’s porpoise (*Phocoenoides dalli*) in Monteray Bay, California. *Aquat. Mamm.* **17**: 12–19.
236. Walker, W. A. 2001. Geographical variation of the parasite, *Phyllobothrium delphini* (Cestoda), in Dall’s Porpoise, *Phocoenoides dalli*, in the northern North Pacific, Berring Sea, and Sea of Okhotsk. *Mar. Mamm. Sci.* **17**: 264–275.
237. Conlogue, G. J., Ogden, J. A., & Foreyt, W. J. 1985. Parasites of the Dall’s Porpoise (*Phocoenoides dalli* True). *J. Wildl. Dis.* **21**: 160–166.
238. Brownell Jr., R. L. 1975. *Phocoena dioprica*. *Mamm. Species* **66**: 1–3.
239. Pinedo, M. C., Barreto, A. S., Lammardo, M. P., Andrade, A. L. V, & Geracitano, L. 2002. Northernmost records of the Spectacled Porpoise, Layard’s Beaked Whale, and Peale's Dolphin in the southwestern Atlantic Ocean. *Aquat. Mamm.* **28**: 32–37.
240. Gao, A., & Zhou, K. 1993. Growth and reproduction of three populations of finless porpoise, *Neophocaena phocaenoides*, in Chinese waters. *Aquat. Mamm.* **19**: 3–12.
241. Yoshida, H., Higashi, N., Ono, H., & Uchida, S. 2010. Finless Porpoise (*Neophocaena phocaenoides*) discovered at Okinawa Island, Japan, with the source population inferred from mitochondrial DNA. *Aquat. Mamm.* **36**: 278–283.
242. Parsons, E., & Jefferson, T. A. 2000. Post-mortem investigations on stranded dolphins and porpoises from Hong Kong waters. *J. Wildl. Dis.* **36**: 342–356.
243. Silber, G. K., & Norris, K. S. 1991. Geographic and seasonal distribution of the Vaquita, *Phocoena sinus*. *An. Inst. Biol.Uni. Nat. Autón. Méx. Ser. Zool.* **62**: 263–268.
244. Silber, G. K. 1990. Occurrence and distribution of the Vaquita *Phocoena sinus* in the Northern Gulf of California. *Fish. Bull.* **88**: 339–346.
245. Silber, G. K. 1988. Recent sightings of the Gulf of California Harbour Porpoise, *Phocoena sinus*. *J. Mammal.* **69**: 430–433.
246. Silber, G. K., Newcomer, M. W., Silber, P. C., Pérez-Cortés M., H., & Ellis, G. M. 1994. Cetaceans of the Northern Gulf of California: distribution, occurence and relative abundance. *Mar. Mamm. Sci.* **10**: 283–298.
247. Silber, G. K., Newcomer, M. W., & Pérez-Cortés M., H. 1990. Killer whales (*Orcinus orca*) attack and kill a Bryde’s whale (*Balaenoptera edeni*). *Can. J. Zool.* **68**: 1603–1606.
248. Santos, M. C. de O, Faria Oshima, J. E. de, & da Silva, E. 2009. Sightings of Franciscana dolphins (*Pontporia blainvillei*): the discovery of a population in the Paranaguá Estuarine Complex, southern Brazil. *Braz. J. Oceano.* **57**: 57–63.
249. Ott, P. H., & Danilewicz, D. 1998. Presence of franciscana dolphins (*Pontporia blainvillei*) in the stomach of a killer whale (*Orcinus orca*) stranded in southern Brazil. *Mammalia* **62**: 605–609.
250. Beneditto, A. P. M. Di. 2004. Presence of Franciscana Dolphin (*Pontoporia blainvillei*) remains in the stomach of a Tiger Shark (*Galeocerdo cuvieri*) captured in southeastern Brazil. *Aquat. Mamm.* **30**: 311–314.
251. Lucifora, L. O., Menni, R. C., & Escalante, A. H. 2005. Reproduction, abundance and feeding habits of the broadnose sevengill shark *Notorynchus cepedianus* in north Patagonia, Argentina. *Mar. Ecol. Prog. Ser.* **289**: 237–244.
252. Santos, M., Oshima, J., Pacífico, E., & Silva, E. 2010. Group size and composition of Guiana dolphins (*Sotalia guianensis*) (Van Bénèden, 1864) in the Paranaguá Estuarine Complex, Brazil. *Braz. J. Biol.* **70**: 111–20.
253. Kaschner, K., Watson, R., Trites, A., & Pauly, D. 2006. Mapping world-wide distributions of marine mammal species using a relative environmental suitability (RES) model. *Mar. Ecol. Prog. Ser.* **316**: 285–310.
254. Dietz, R., & Heide-Jørgensen, M. P. 1995. Movements and swimming speed of Narwhals, *Monodon monoceros*, equipped with satellite transmitters in Melville Bay, northwast Greenland. *Can. J. Zool.* **73**: 2106–2119.
255. Rako, N., Draško, H., & Fortuna, C. M. 2009. Long-term inshore observation of a solitary Striped Dolphin, *Stenella coeruleoalba*, in the Vinodol Channel, Northern Adriatic Sea (Croatia). *Nat. Croat.***18**: 427–436.
256. Frantzis, A., & Herzing, D. L. 2002. Mixed-species associations of Striped dolphins (*Stenella coeruleoalba*), Short-beaked Common dolphins (*Delphinus delphis*), and Risso’s dolphins (*Grampus griseus*) in the Gulf of Corinth (Greece, Mediterranean Sea). *Aquat. Mamm.* **28**: 188–197.
257. Rosas, F. C. W., Monteiro-Filho, E. L. A., Marigo, J., Santos, R. A., Andrade, A. L. V, Rautenberg, M., *et al.* 2002. The striped dolphin, *Stenella coeruleoalba* (Cetacea: Delphinidae), on the coast of São Paulo State, southeastern Brazil. *Aquat. Mamm.* **28**: 60–66.
258. Dwyer, S. L., & Visser, I. N. 2011. Cookie Cutter Shark (*Isistius sp*.) bites on cetaceans, with particular reference to Killer Whales (Orca) (*Orcinus orca*). *Aquat. Mamm.* **37**: 111–138.
259. Crovetto, A., Lamilla, J., & Pequeño, G. 1992. *Lissodelphis peronii*, Lacépède 1804 (Delphinidae, Cetacea) within the stomach contents of a sleeping shark, *Somniosus CF. pacificus*, Bigelow and Schroeder 1944, in Chilean Waters. *Mar. Mamm. Sci.* **8**: 312–314.
260. Migura, K. A., & Meadows, D. W. 2002. Short-finned Pilot whales (*Globicephala macrorhynchus*) interact with Melon-Headed whales (*Peponocephala electra*) in Hawaii. *Aquat. Mamm.* **28**: 294–297.
261. Steiger, G. H., Calambokidis, J., Straley, J. M., Herman, L. M., Cerchio, S., Salden, D. R., *et al.* 2008. Geographic variation in killer whale attacks on humpback whales in the North Pacific: implications for predation pressure. *Endanger. Species Res.* **4**: 247–256.
262. Weir, C. R. 2010. First description of Atlantic Humpback Dolphin *Sousa Teuszii* whistles, recorded off Angola. *Bioacoustics* **19**: 211–224.
263. Perrin, W. F., & Walker, W. A. 1975. The Rough-Toothed Porpoise, *Steno bredanensis*, in the eastern tropical Pacific. *J. Mammal.* **56**: 905–907.
264. Ritter, F. 2002. Behavioural observation of rough-toother dolphins (*Steno bredanensis*) off La Gomera, Canery Islands (1995-2000), with special reference to their interactions with humans. *Aquat. Mamm.* **28**: 46–59.
265. Celona, A., Maddalena, A. De, & Comparetto, G. 2006. Evidence of predatory attack on a Bottlenose Dolphin *Tursiops truncatus* by a Great White shark *Carcharodon carcharias* in the Mediterranean Sea. *Annales Ser. Hist. Nat.* **16**: 159–164.
266. Bruce, B. D. 1992. Preliminary observations on the biology of the White Shark, *Carcharodon carcharias*, in South Australian waters. *Aust. J. Mar. Freshw. Res.* **43**: 1–11.
267. Rossi-Santos, M. R., Santos-Neto, E., & Baracho, C. G. 2009. Interspecific cetacean interactions during the breeding season of humpback whale (*Megaptera novaeangliae*) on the north coast of Bahia State, Brazil. *J. Mar. Biol. Assoc. UK* **89**: 961–966.
268. Gannier, A. 2002. Cetaceans of the Marquesas Islands (French Polynesia): distribution and relative abundance as obtained from a small boat dedicated survey. *Aquat. Mamm.* **28**: 198–210.
269. Ciano, J., & Jørgensen, R. 2000. Observations on an interaction between a Humpback Whale (*Megaptera novaeangliae*) and Pilot Whales (*Globicephala melas*). *Mar. Mamm. Sci.* **16**: 245–248.
270. Williams, A. D., Williams, R., & Brereton, T. 2002. The sighting of pygmy killer whales (*Feresa attenuata*) in the southern Bay of Biscay and their association with cetacean calves. *J. Mar. Biol. Assoc. UK* **82**: 509–511.
271. Reyes, L. M., & García-Borboroglu, P. 2004. Killer Whale (*Orcinus orca*) predation on sharks in Patagonia, Argentina: a first report. *Aquat. Mamm.* **30**: 376–379.
272. Perryman, W. L., Au, D. W. K., Leatherwood, S., & Jefferson, T. A. 1994. Melon-headed Whale *Peponocephala electra* Gray, 1846. In R. G. Harrison & S. H. Ridgway (Eds.), *Handbook of Marine Mammals: Volume 5 The first book of dolphins*. San Diego, CA: Academic Press Inc. (pp. 363–386).
273. Wang, J. Y., Yang, S. C., Hung, S., & Jefferson, T. A. 2007. Distribution, abundance and conservation status of the eastern Taiwan Strait population of Indo-Pacific humpback dolphins, *Sousa chinensis*. *Mammalia*: 157–165.
274. Goodall, R. N. P. 1994. Commerson’s Dolphin *Cephalorhynchus commersonii* (Lacépède 1804). In S. H. Ridgway & R. G. Harrison (Eds.), *Handbook of Marine Mammals: Volume 5 The first book of dolphins*. San Diego, CA: Academic Press Inc. (pp. 241–267).
275. Santora, J. A. 2012. Habitat use of hourglass dolphins near the South Shetland Islands, Antarctica. *Polar Biol.* **35**: 801–806.
276. Hale, P. T., Barreto, A. S., & Ross, G. J. B. 2000. Comparative morphology and distribution of the *aduncus* and *truncatus* forms of bottlenose dolphin *Tursiops* in the Indian and Western Pacific Oceans. *Aquat. Mamm.* **26**: 101–110.
277. Dulau-Drouot, V., Boucaud, V., & Rota, B. 2008. Cetacean diversity off La Réunion Island (France). *J. Mar. Biol. Assoc. UK* **88**: 1263–1272.
278. Matsuoka, K., Kiwada, H., Fujise, Y., & Miyashita, T. 2007. Distribution of blue (Balaenoptera musculus), fin (B. physalus), humpback (Megaptera novaeangliae) and north pacific right (Eubalaena japonica) whales in the western North Pacific based on JARPN and JARPN II sighting surveys (1994 to 2007). *Inst. Cet. Res.* SC J09/JR35
279. Tynan, C. T., DeMaster, D. P., & Peterson, W. T. 2001. Endangered right whales on the southeastern Bering Sea shelf. *Science* **294**: 1894.
280. Borstad, G. A. 1985. Water colour and temperature in the Southern Beaufort Sea: Remote sensing in support of ecological studies of the Bowhead Whale. *DFO Can. Tech. Rep. Fish. Aquat. Sci.*
281. Laidre, K. L., Heide-Jørgensen, M. P., Logsdon, M. L., Delwiche, L., & Nielsen, T. G. 2010. A whale of an opportunity: Examining the vertical structure of chlorophyll-a in high Arctic waters using instrumented marine predators. *Mar. Biol. Res.* **6**: 519–529.
282. Jayasankar, P., Krishnan, A. A., Rajagopalan, M., & Krishnakumar, P. K. 2007. A note on observations on cetaceans in the western Indian sector of the Southern Ocean (20-56°S and 45-57°30’E ), January to March 2004. *J. Biogeogr.* **9**: 263–267.
283. Watanabe, H., Okazaki, M., Tamura, T., Konishi, K., Inagake, D., Bando, T., *et al.* 2012. Habitat and prey selection of common minke, sei, and Bryde’s whales in mesoscale during summer in the subarctic and transition regions of the western North Pacific. *Fish. Sci.* **78**: 557–567.
284. Molina-Schiller, D., Rosales, S. A., & Freitas, T. R. O. de. 2005. Oceanographic conditions off coastal South America in relation to the distribution of Burmeister’s porpoise, *Phocoena spinipinnis*. *LAJAM* **4**: 141–156.
285. Weir, C. R. 2009. Distribution, behaviour and photo-identification of Atlantic humpback dolphins *Sousa teuszi*i off Flamingos, Angola. *Afr. J. Mar. Sci.* **31**: 319–331.
286. Baldwin, R. M., Collins, M., Van Waerebeek, K., & Minton, G. 2004. The Indo-Pacific Humpback Dolphin of the Arabian Region: A status review. *Aquat. Mamm.* **30**: 111–124.
287. Cribb, N., Miller, C., & Seuront, L. 2008. Assessment of bottlenose dolphin (*Tursiops aduncus*) habitat characteristics in the estuarine waters of the Adelaide Dolphin Sanctuary, South Australia. *JMATE* **1**: 6–8.
288. Baumgartner, M. F., Van Parijs, S. M., Wenzel, F. W., Tremblay, C. J., Esch, H. C., & Warde, A. M. 2008. Low frequency vocalizations attributed to sei whales (*Balaenoptera borealis*). *J. Acoust. Soc. Am.* **124**: 1339–1349.
289. Erbe, C. 2004. The acoustic repertoire of odontecetes as a basis for developing automatic detectors and classifiers. *DRDC Atl CR 2004-071*
290. Goodall, R. N. P. 2002. Peale’s Dolphin *Lagenorhynchus australis*. In W. F. Perrin, B. Würsig, & J. Thewissen (Eds.), *Encyclopedia of Marine Mammals*. San Francisco, CA: Academic Press Inc. (pp. 844–847).
291. Penry, G. S., Cockcroft, V. G., & Hammond, P. S. 2011. Seasonal fluctuations in occurrence of inshore Bryde’s whales in Plettenberg Bay, South Africa, with notes on feeding and multispecies associations. *Afr. J. Mar. Sci.* **33**: 403–414.
292. Smultea, M. A., Douglas, A. B., Bacon, C. E., Jefferson, T. A., & Mazzuca, L. 2012. Bryde’s Whale (*Balaenoptera brydei/edeni*) sightings in the southern California bight. *Aquat. Mamm.* **38**: 92–97.
293. Stephanis, R. de, Cornulier, T., Verborgh, P., Salazar Sierra, J., Gimeno, N. P., & Guinet, C. 2008. Summer spatial distribution of cetaceans in the Strait of Gibraltar in relation to the oceanographic context. *Mar. Ecol. Prog. Ser.* **353**: 275–288.
294. Lachmuth, C. L., Alava, J. J., Hickie, B. E., Johannessen, S. C., Macdonald, R. W., Ford, J. K. B., *et al.* 2010. Ocean disposal in resident killer whale (Orcinus orca) Critical Habitat: Science in support if risk management. *Canadian Science Advisory Secretariat Research Document 2010/116*
295. Kreb, D., & Budiono. 2005. Cetacean diversity and habitat preferences in tropical waters of east Kalimantan, Indonesia. *Raff. Bull. Zool.* **53**: 149–155.
296. Sciara, G. N. Di, Venturino, M. C., Zanardelli, M., Bearzi, G., Borsani, F. J., & Cavalloni, B. 2009. Cetaceans in the central Mediterranean Sea: Distribution and sighting frequencies. *Boll. Zool.* **60**: 37–41.
297. Elwen, S. H., Thornton, M., Reeb, D., & Best, P. B. 2010. Near-Shore Distribution of Heaviside’s (*Cephalorhynchus heavisidii*) and Dusky Dolphins (*Lagenorhynchus obscurus*) at the Southern Limit of their Range in South Africa. *Afr. Zool.* **45**: 78–91.
298. Frantzis, A., Alexiadou, P., Paximadis, G., Politi, E., Gannier, A., & Corsini-Foka, M. 2003. Current knowledge of the cetacean fauna of the Greek Seas. *J. Cetacean Res. Manag.* **5**: 219–232.
299. Frazer, J. F. D. 1976. Herd structure and behaviour in cetaceans. *Mamm. Rev.* **6**: 55–59.
300. Hooker, S. K., Whitehead, H., & Gowans, S. 1999. Marine protected area design and the spatial and temporal distribution of cetaceans in a submarine canyon. *Conserv. Biol.* **13**: 592–602.
301. Moore, M. J., Berrow, S. D., Jensen, B. A., Carr, P., Sears, R., Rowntree, V. J., *et al.* 1999. Relative abundance of large whales around South Georgia (1979-1998). *Mar. Mamm. Sci.* **15**: 1287–1302.
302. Best, P. B. 1974. Two allopatric forms of Bryde’s whales off South Africa. *Rep. Int. Whal. Commn.* **Special Is**: 10–38.
303. Dahlheim, M. E., & Heyning, J. E. 1999. Killer Whale *Orcinus orca* (Linnaeus, 1758). In S. H. Ridgway & R. Harrison (Eds.), *Handbook of Marine Mammals: Volume 6 The second book of dolphins and the porpoises*. Toronto, ON: Academic Press Inc. (pp. 281–322).
304. Jefferson, T. A., Stacey, P. J., & Baird, R. W. 1991. A review of Killer Whale interactions with other marine mammals: predation to co-existence. *Mamm. Rev.* **21**: 151–180.
305. Visser, I. N., Berghan, J., Meurs, R. Van, & Fertl, D. 2000. Killer whale (*Orcinus orca*) predation on a shortfin mako shark (*Isurus oxyrinchus*) in New Zealand waters. *Aquat. Mamm.* **26**: 229–231.
306. Ford, J. J. B., & Ellis, G. M. 2006. Selective foraging by fish-eating killer whales *Orcinus orca* in British Columbia. *Mar. Ecol. Prog. Ser.* **316**: 185–199.
307. Ross, G. J. B., Heinsohn, G. E., & Cockcroft, V. G. 1994. Humpback Dolphins *Sousa chinensis* (Osbeck,1765), *Sousa plumbea* (G. Cuvier, 1829) and *Sousa teuszii* (Kukenthal, 1892). In S. H. Ridgway & R. Harrison (Eds.), *Handbook of Marine Mammals: Volume 5 The first book of dolphins*. Toronto, ON: Academic Press Inc. (pp. 23–42).
308. Pitman, R. L., Helden, A. L. van, Best, P. B., & Pym, A. T. 2006. Shepherd’s Beaked Whale (*Tasmacetus shepherdi*): information on appearance and biology based on strandings and at-sea observations. *Mar. Mamm. Sci.* **22**: 744–755.
309. Yatabe, A., Kubo, N., Otsuka, M., Shima, S., Kubodera, T., & Yamada, T. K. 2010. Stomach contents and structure of a Longman’s Beaked Whale (*Indopacetus pacificus*) stranded in Kyushu, Japan. *Aquat. Mamm.* **36**: 172–177.
310. Loughlin, T., & Perez, M. A. 1985. *Mesoplodon stejnegeri*. *Mamm. Species* **250**: 1–6.
311. Jefferson, T. A., & Newcomer, M. W. 1993. *Lissodelphis borealis*. *Mamm. Species* **425**: 1–6.
312. Jackson, A., Gerrodette, T., Chivers, S., Lynn, M., Rankin, S., & Mesnick, S. 2006. Marine mammal data collected during a survery in the eastern tropical Pacific Ocean aboard NOAA ships David Starr Jordan and McArthur II, July 28-December 7, 2006. *NOAA Tech. Mem.* NMFS-SWFSC-421
313. Reyes, J. C., Mead, J. G., & Waerebeek, K. Van. 1991. A new species of beaked whale *Mesoplodon peruvianus sp. N*. (Cetacea: Ziphiidae) from Peru. *Mar. Mamm. Sci.* **7**: 1–24.
314. Carrillo, M. 2003. Presence and distribution of the Ziphiidae family in the southwest coast of Tenerife. Canary Islands. In *17th Conference of the European Cetacean Society.* (pp. 1–5).
315. Moore, J. C., & Wood Jr, F. 1957. Differences between the beaked whales Mesoplodon mirus and *Mesoplodon gervaisi*. *Am. Mus. Novit.* **1831**: 1–25.
316. Würsig, B., Jefferson, T. A., & Schmidly, D. J. 2000. *The Marine Mammals of the Gulf of Mexico*. College Station: Texas A&M University Press.
317. Stacey, P. J., & Baird, R. W. 1991. Status of the Pacific White-sided dolphin, *Lagenorhynchus obliquidens*, in Canada. *Can. Field Nat.* **105**: 219–232.
318. Stacey, P. J., & Baird, R. W. 1991. Status of the False Killer Whale, *Pseudorca crassidens*, in Canada. *Canadian Field-Naturalist* **105**: 189–197.
319. Heithaus, M. R. 2001. Predator-prey and competitive interactions between sharks (order Selachii) and dolphins (suborder Odontoceti): a review. *J. Zool., Lond.* **253**: 53–68.
320. Campbell, R. R., Yurik, D. B., & Snow, N. B. 1988. Predation on Narwhals, *Monodon monoceros*, by Killer Whales, *Orcinus orca*, in the eastern Canadian Arctic. *Can. Field Nat.* **102**: 689–696.
321. Khan, M., Panda, S., Pattnaik, A. K., Guru, B. C., Kar, C., Subudhi, M., *et al.* 2011. Shark attacks on Irrawaddy dolphin in Chilika lagoon, India. *J. Mar. Biol. Assoc. Ind.* **53**: 27–34.
322. Abrantes, K., & Barnett, A. 2011. Intrapopulation variations in diet and habitat use in a marine apex predator, the broadnose sevengill shark *Notorynchus cepedianus*. *Mar. Ecol. Prog. Ser.* **442**: 133–148.
323. Weller, D. W. 2002. Predation on Marine Mammals. In W. F. Perrin, B. Würsig, & J. G. M. Thewissen (Eds.), *Encyclopedia of Marine Mammals*. San Francisco, CA: Academic Press Inc. (pp. 985–994).
324. Kemper, C. M. 2002. Pygmy Right Whale, *Caperea marginata*. In W. F. Perrin, B. Würsig, & J. G. M. Thewissen (Eds.), *Encyclopedia of Marine Mammals*. San Francisco, CA: Academic Press Inc. (pp. 1010–1012).
325. Watts, P. D., Draper, B. A., & Henrico, J. 1991. Preferential use of warm water habitat by adult Beluga Whales. *J. Therm. Biol.* **16**: 57–60.
326. Lopez, S., Meléndez, R., & Barría, P. 2009. Alimentación del tiburón marrajo *Isurus oxyrinchus* Rafinesque, 1810 (Lamniformes : Lamnidae) en el Pacífico suroriental. *Rev. Biol. Mar. Oceanogr.* **44**: 439–451.
327. Rice, D. W. 1989. Sperm Whale *Physeter macrocephalus* Linnaeus, 1758. In S. H. Ridgway & R. Harrison (Eds.), *Handbook of Marine Mammals: Volume 4 River dolphins and the larger toothed whales*. Toronto, ON: Academic Press Inc. (pp. 177–233).
328. Caldwell, D. K., & Caldwell, M. C. 1989. Pygmy Sperm Whale *Kogia breviceps* (de Blainville, 1838): Dwraf Sperm Whale Kogia simus Owen, 1866. In S. H. Ridgway & R. Harrison (Eds.), *Handbook of Marine Mammals: Volume 4 River dolphins and the larger toothed whales*. Toronto, ON: Academic Press Inc. (pp. 235–260).
329. Nagorsen, D. 1985. *Kogia Simus*. *Mamm. Species* **239**: 1–6.
330. Bloodworth, B. E., & Odell, D. K. 2008. *Kogia Breviceps* (Cetacea: Kogiidae). *Mamm. Species* **819**: 1–12.
331. Hohn, A. A., Rotstein, D. S., Harms, C. A., & Southall, B. L. 2006. Report on Marine Mammal Unusual Mortality Event UMESE0501Sp: Multispecies Mass Stranding of Pilot Whales (Globicephala macrorhynchus), Minke Whale (Balaenoptera acutorostrata), and Dwarf Sperm Whales (Kogia sima) in North Carolina on 15-16 January 2005. *NOAA Tech. Mem.* NMFS-SEFSC-537
332. Jaquet, N., & Gendron, D. 2002. Distribution and relative abundance of sperm whales in relation to key environmental features, squid landings and the distribution of other cetacean species in the Gulf of California, Mexico. *Mar. Biol.* **141**: 591–601.
333. Cañadas, A., Sagarminaga, R., Stephanis, R. De, Urquiola, E., & Hammond, P. S. 2005. Habitat preference modelling as a conservation tool: proposals for marine protected areas for cetaceans in southern Spanish waters. *Aquat. Conserv.: Mar. Freshwat. Ecosyst.* **15**: 495–521.
334. Whitehead, H. 2002. Sperm Whale *Physeter macrocephalus*. In W. F. Perrin, B. Würsig, & J. G. M. Thewissen (Eds.), *Encyclopedia of Marine Mammals*. San Francisco, CA: Academic Press Inc. (pp. 1165–1172).
335. Taylor, B. L., Baird, R. W., Barlow, J., Dawson, S. M., Ford, J., Mead, J. G., *et al.* 2008. *Kogia sima*. In *IUCN 2012. IUCN Red List of Threatened Species*.
336. Mead, J. G. 1989. Bottlenose Whales *Hyperoodon ampullatus* (Forster, 1770) and Hyperoodon planifrons Flower, 1882. In S. Ridgway & R. G. Harrison (Eds.), *Handbook of Marine Mammals: Volume 4 River dolphins and the larger toothed whales*. Toronto, ON: Academic Press Inc. (pp. 321–349).
337. Vidal, O., Brownell Jr., R. L., & Findley, L. T. 1999. Vaquita *Phocoena sinus* Norris and McFarland, 1958. In S. H. Ridgway & R. G. Harrison (Eds.), *Handbook of Marine Mammals: Volume 6 The second book of dolphins and the porpoises*. Toronto, ON: Academic Press Inc. (pp. 357–378).
338. Frantzis, A., Goold, J. C., Skarsoulis, E. K., Taroudakis, M. I., & Kandia, V. 2002. Clicks from Cuvier’s beaked whales, *Ziphius cavirostris* (L). *J. Acoust. Soc. Am.* **112**: 34–37.
339. Dawson, S. M., Barlow, J., & Ljungblad, D. 1998. Sounds recorded from Baird’s beaked whale, *Berardius bardii*. *Mar. Mamm. Sci.* **14**: 335–344.
340. May-Collado, L. J., Agnarsson, I., & Wartzok, D. 2007. Reexamining the relationship between body size and tonal signals frequency in whales: a comparative approach using a novel phylogeny. *Mar. Mamm. Sci.* **23**: 524–552.
341. Oswald, J. N., Barlow, J., & Norris, T. F. 2003. Acoustic identification of nine delphinid species in the eastern tropical Pacific Ocean. *Mar. Mamm. Sci.* **19**: 20–37.
342. Watkins, W. A., Schevill, W. E., & Best, P. B. 1977. Underwater sounds of *Cephalorhynchus heavisidii* (Mammalia: Cetacea). *J. Mammal.* **58**: 316–320.
343. Rendell, L. E., Matthews, J. N., Gill, A., Gordon, J. C. D., & Macdonald, D. W. 1999. Quantitative analysis of tonal calls from five odontocete species, examining interspecific and intraspecific variation. *J. Zool., Lond.* **249**: 403–410.
344. Leatherwood, S., Jefferson, T. A., Norris, J. C., Stevens, W. E., Hansen, L. J., & Mullin, K. D. 1993. Occurrence and sounds of Fraser’s dolphins (Lagenodelphis hosei) in the Gulf of Mexico. *The Texas Journal of Science* **45**: 349–354.
345. Herzing, D. L. 1996. Vocalizations and associated underwater behavior of free-ranging Atlantic Spotted dolphins, *Stenella frontalis* and Bottlenose dolphins, *Tursiops truncatus*. *Aquat. Mamm.* **22**: 61–79.
346. May-Collado, L. J., Agnarsson, I., & Wartzok, D. 2007. Phylogenetic review of tonal sound production in whales in relation to sociality. *BMC Evol. Biol.* **7**: 136.
347. Rankin, S., Oswald, J., Barlow, J., & Lammers, M. 2007. Patterned burst-pulse vocalizations of the northern right whale dolphin, *Lissodelphis borealis*. *J. Acoust. Soc. Am.* **121**: 1213.

Appendix S2 – Survey Template for Professional Opinion of Strength of Driving Factors

Thanks for Helping! As part of my Master’s I am investigating whether cetacean species that share particular evolutionary or ecological traits might be more likely to hybridize with one another. Part of doing this requires weighting different traits according to their likelihood of influencing interspecific mating. In order to come up with such a weighting, I am looking for the support of fellow biologists to provide their own opinion on the relative importance of these traits in promoting hybridization. I'm asking you to rate on a scale from 0-10 how important each trait might be in influencing hybridization. Species Traits:

|  | Less Important  1 | 2 | 3 | 4 | 5 | 6 | 7 | 8 | 9 | Very Important  10 | No Influence  0 |
| --- | --- | --- | --- | --- | --- | --- | --- | --- | --- | --- | --- |
| Male Body Length (at physical maturity) |  |  |  |  |  |  |  |  |  |  |  |
| Female Body Length (at physical maturity) |  |  |  |  |  |  |  |  |  |  |  |
| Sexual Dimorphism (in colour or size) |  |  |  |  |  |  |  |  |  |  |  |
| Preferred Water Depth |  |  |  |  |  |  |  |  |  |  |  |
| Preferred Water Temperature |  |  |  |  |  |  |  |  |  |  |  |
| Prey Species |  |  |  |  |  |  |  |  |  |  |  |
| Predator Species |  |  |  |  |  |  |  |  |  |  |  |
| Parasite Species |  |  |  |  |  |  |  |  |  |  |  |
| Mean Group Size |  |  |  |  |  |  |  |  |  |  |  |
| Species Range Size |  |  |  |  |  |  |  |  |  |  |  |
| Cetacean Species Known to Interact With |  |  |  |  |  |  |  |  |  |  |  |
| Shared Range Overlap |  |  |  |  |  |  |  |  |  |  |  |
| Vocalization Frequency |  |  |  |  |  |  |  |  |  |  |  |

Table S3. Eigenvectors of the first four principal components of variation in similarity of traits for all cetacean species comparisons by taking the absolute value of the eigenvectors averaged across 10,000 subsampled principal component analyses where each species was only represented once. Variables that are more important for each principal component have larger values (+ or -). (N = 78 species represented in each iteration)

| Trait (All species) | PC1 | PC2 | PC3 | PC4 |
| --- | --- | --- | --- | --- |
| Male Body Length | 0.1953 | 0.1842 | 0.1753 | 0.1671 |
| Female Body Length | 0.2086 | 0.1961 | 0.1807 | 0.1670 |
| Sexual Dimorphism | 0.4623 | 0.2998 | 0.2266 | 0.2005 |
| Range Size | 0.3079 | 0.2069 | 0.1714 | 0.1664 |
| Water Depth | 0.2081 | 0.2537 | 0.2943 | 0.3212 |
| Water Temperature | 0.3076 | 0.3398 | 0.3357 | 0.3063 |
| Prey Species | 0.0368 | 0.0464 | 0.0606 | 0.0817 |
| Predator Species | 0.1227 | 0.1518 | 0.1834 | 0.2290 |
| Parasite Species | 0.0730 | 0.0876 | 0.1084 | 0.1376 |
| Average Group Size | 0.2023 | 0.2361 | 0.2664 | 0.2902 |
| Known Associate Species | 0.0394 | 0.0505 | 0.0653 | 0.0882 |
| Natural Range Overlap | 0.3101 | 0.3261 | 0.2976 | 0.2616 |
| Vocalization Frequency | 0.2572 | 0.2963 | 0.3123 | 0.2981 |

Table S4. Eigenvectors of the first four principal components of variation in similarity of traits for cetacean species with 44 chromosomes by taking the absolute value of the eigenvectors averaged across 10,000 subsampled principal component analyses where each species was only represented once. Variables that are more important for each principal component have larger values (+ or -). (N = 52 species represented in each iteration)

| Trait (2n=44) | PC1 | PC2 | PC3 | PC4 |
| --- | --- | --- | --- | --- |
| Male Body Length | 0.2315 | 0.2118 | 0.2023 | 0.2009 |
| Female Body Length | 0.2468 | 0.2224 | 0.2047 | 0.1999 |
| Sexual Dimorphism | 0.4584 | 0.3169 | 0.2447 | 0.2038 |
| Range Size | 0.3048 | 0.2159 | 0.1755 | 0.1591 |
| Water Depth | 0.1879 | 0.2254 | 0.2693 | 0.3208 |
| Water Temperature | 0.2838 | 0.3328 | 0.3507 | 0.3237 |
| Prey Species | 0.0347 | 0.0419 | 0.0533 | 0.0696 |
| Predator Species | 0.1192 | 0.1422 | 0.1695 | 0.2065 |
| Parasite Species | 0.0748 | 0.0845 | 0.0999 | 0.1217 |
| Average Group Size | 0.1960 | 0.2207 | 0.2417 | 0.2746 |
| Known Associate Species | 0.0377 | 0.0466 | 0.0567 | 0.0721 |
| Natural Range Overlap | 0.2900 | 0.3215 | 0.3167 | 0.2866 |
| Vocalization Frequency | 0.2980 | 0.3243 | 0.3194 | 0.2955 |


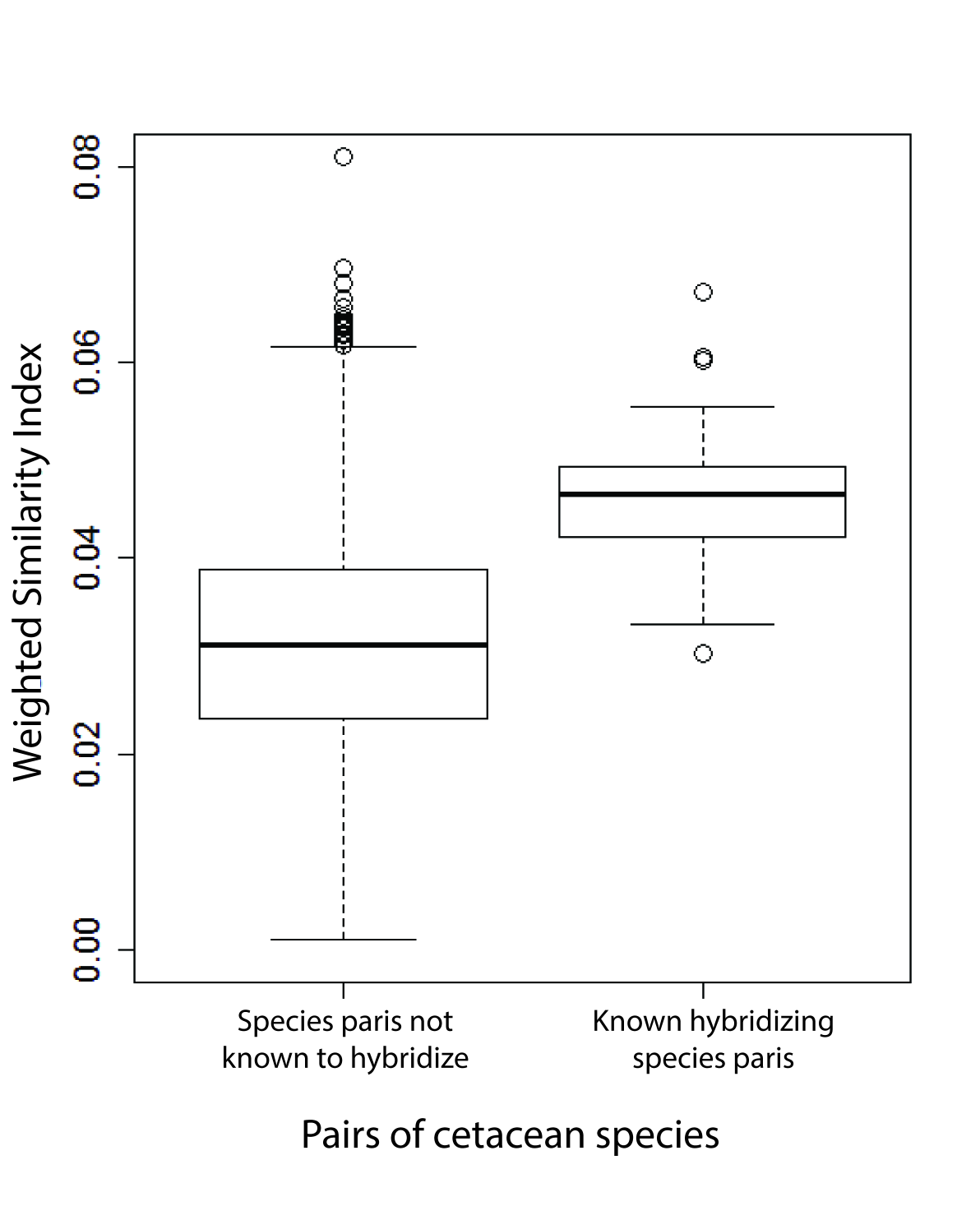


Non-hybridizing
species pairs

Hybridizing
species pairs

Fig. S5. Weighted similarity index of non-hybridizing species pairs (n = 6048) and hybridizing species pairs (n = 36) for all species comparisons. Each pair of species is represented twice, allowing each species to be both Species 1 and Species 2 in building the similarity index.

Table S6. Eigenvectors of the first four principal components of variation in the weighted similarity of traits for all cetacean species comparisons (N = 78). Variables that are more important for each principal component have larger values (+ or -).

| Trait (ALL) | PC1 | PC2 | PC3 | PC4 |
| --- | --- | --- | --- | --- |
| Male Body Length | -0.066 | -0.326 | 0.331 | -0.289 |
| Female Body Length | -0.019 | -0.299 | 0.349 | -0.279 |
| Sexual Dimorphism | 0.254 | -0.647 | -0.527 | -0.059 |
| Range Size | 0.138 | -0.314 | -0.258 | -0.027 |
| Water Depth | -0.005 | -0.197 | -0.080 | 0.239 |
| Water Temperature | -0.306 | -0.247 | 0.130 | 0.831 |
| Prey Species | -0.013 | -0.091 | 0.036 | -0.010 |
| Predator Species | -0.042 | -0.047 | -0.009 | 0.011 |
| Parasite Species | -0.010 | -0.066 | 0.026 | -0.023 |
| Average Group Size | -0.043 | -0.137 | 0.147 | 0.072 |
| Known Associate Species | -0.048 | -0.182 | 0.065 | 0.024 |
| Natural Range Overlap | -0.900 | -0.109 | -0.249 | -0.283 |
| Vocalization Frequency | 0.040 | -0.329 | 0.558 | -0.028 |
| Proportion of Variation Accounted For | 26.01% | 21.77% | 16.64% | 10.65% |

Table S7. Eigenvectors of the first four principal components of variation in the weighted similarity of traits for cetacean species comparisons with 44 chromosomes (N = 52). Variables that are more important for each principal component have larger values (+ or -).

| Trait (2n = 44) | PC1 | PC2 | PC3 | PC4 |
| --- | --- | --- | --- | --- |
| Male Body Length | -0.075 | 0.376 | 0.245 | 0.404 |
| Female Body Length | -0.009 | 0.364 | 0.256 | 0.379 |
| Sexual Dimorphism | 0.169 | 0.518 | -0.681 | 0.020 |
| Range Size | 0.092 | 0.253 | -0.333 | 0.018 |
| Water Depth | -0.007 | 0.157 | -0.128 | -0.171 |
| Water Temperature | -0.319 | 0.223 | 0.072 | -0.749 |
| Prey Species | -0.019 | 0.096 | 0.007 | 0.009 |
| Predator Species | -0.055 | 0.041 | -0.021 | -0.010 |
| Parasite Species | -0.012 | 0.072 | 0.008 | 0.005 |
| Average Group Size | -0.051 | 0.167 | 0.105 | -0.121 |
| Known Associate Species | -0.074 | 0.185 | 0.014 | -0.013 |
| Natural Range Overlap | -0.915 | 0.022 | -0.169 | 0.223 |
| Vocalization Frequency | 0.082 | 0.491 | 0.487 | -0.193 |
| Proportion of Variation Accounted For | 24.49% | 22.84% | 17.63% | 10.72% |
